# Supplementary material for: Associations between active travel and diet: cross-sectional evidence on healthy, low-carbon behaviours from UK Biobank
Source: BMJ Open. 2019 Sep 3;9(8):e030741. doi: 10.1136/bmjopen-2019-030741 (PMC6731823; doi:10.1136/bmjopen-2019-030741)
Supplement: Supplementary data [file bmjopen-2019-030741supp001.pdf]

Figure S1 – Putative relationships between active travel, dietary consumption, physical activity, energy intake and other covariates

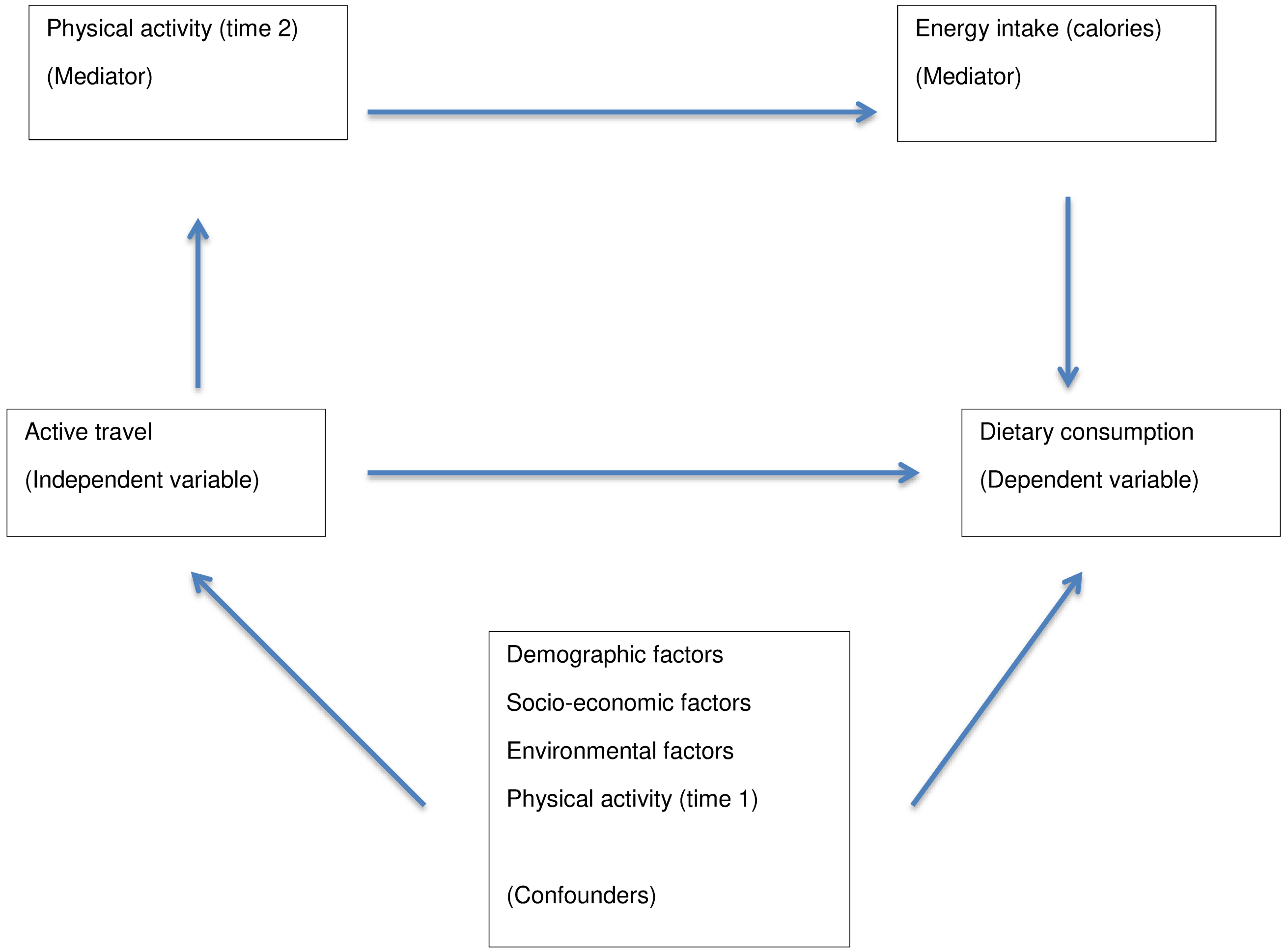

Figure S1 provides an overview of the putative relationships between active travel, dietary consumption, physical activity, energy intake and other covariates in this study.

People who are more physically active tend to have healthier diets [1] and may be more likely to engage in active travel [2] – this means that one's initial physical activity level (time 1) may be a confounder of the relationship between active travel and dietary consumption, in the same way that demographic, socio-economic, and environmental factors may also act as confounders.

At the same time, we also know that those who engage in active travel may accumulate additional physical activity [3], which may ultimately lead them to consume more food and have a higher energy intake (time 2). In this way, it is possible for physical activity and energy intake to act as mediators of the relationship between active travel and dietary consumption.

It is not possible to tease out these distinctions with cross-sectional data, where all of these variables have been measured at the same point in time. To account for this limitation, we have presented three models with different levels of covariate adjustment: Model 1 is unadjusted, Model 2 is adjusted for demographic, socio-economic and environmental factors, and Model 3 (sensitivity analysis) is adjusted for physical activity level and energy intake.

References

1. Noble N, Paul C, Turon H, et al. Which modifiable health risk behaviours are related? A systematic review of the clustering of Smoking, Nutrition, Alcohol and Physical activity ('SNAP') health risk factors. Prev Med 2015;**81**:16-41 doi: 10.1016/j.ypmed.2015.07.003[published Online First: Epub Date]|.

2. Hutchinson J, Prady SL, Smith MA, et al. A Scoping Review of Observational Studies Examining Relationships between Environmental Behaviors and Health Behaviors. Int J Environ Res Public Health 2015;**12**(5):4833-58

3. Sahlqvist S, Goodman A, Cooper AR, et al. Change in active travel and changes in recreational and total physical activity in adults: longitudinal findings from the iConnect study. International Journal of Behavioral Nutrition and Physical Activity 2013;**10**(1):28 doi: 10.1186/1479-5868-10-28[published Online First: Epub Date]|.

Table S1 – Results of ordinal logistic models between any active travel and fruit and vegetable (FV) consumption among females in UKB (n=217,168)

VARIABLES	Model 1	Model 2
Any active travel (ref: None)	1.42***	1.43***
	(1.40 - 1.44)	(1.40 - 1.45)
Age at baseline		1.04***
		(1.04 - 1.05)
Ethnic group (ref: White British)		
Other white		1.38***
		(1.33 - 1.42)
South Asian		2.19***
		(2.04 - 2.37)
Black		1.63***
		(1.52 - 1.74)
Chinese		1.67***
		(1.45 - 1.93)
Mixed		1.16**
		(1.05 - 1.28)
Other		2.29***
		(2.08 - 2.53)
Highest qualification (ref: Degree level)		
A levels/AS levels or equivalent		0.86***
		(0.83 - 0.88)
O levels/GCSEs or equivalent		0.77***
		(0.75 - 0.79)
CSEs or equivalent		0.72***
		(0.70 - 0.75)
NVQ or HND or HNC or equivalent		0.79***
		(0.76 - 0.82)
Other professional qualifications		0.89***
		(0.86 - 0.92)
No qualifications		0.63***
		(0.62 - 0.65)
Occupation class (ref: Higher man / prof)		
Lower managerial / professional		1.08***
		(1.05 - 1.11)
Intermediate occupations		1.02
		(0.99 - 1.06)
Small employers & own accounts		1.11***
		(1.04 - 1.17)
Lower supervisory & technical		1.16*
		(1.03 - 1.31)
Semi-routine occupations		1.03
		(0.99 - 1.07)
Routine occupations		0.98
		(0.93 - 1.04)
Not classified		1.03
		(1.00 - 1.06)
Household income (ref: £<18 000)		
£18,000 to 30,999		1.10***
		(1.07 - 1.13)
£31,000 to 51,999		1.21***
		(1.17 - 1.24)
£52,000 to 100,000		1.29***
		(1.25 - 1.33)
£Greater than 100,000		1.32***
		(1.26 - 1.38)

Household size (ref: One)		
2		0.95***
		(0.93 - 0.97)
3		0.88***
		(0.86 - 0.91)
4		0.85***
		(0.82 - 0.87)
5+		0.87***
		(0.83 - 0.91)
Region (ref: London)		
North East England		1.07***
		(1.03 - 1.11)
Yorkshire and the Humber		0.99
		(0.96 - 1.02)
West Midlands		1.06***
		(1.03 - 1.10)
East Midlands		1.08***
		(1.04 - 1.12)
South East England		1.12***
		(1.08 - 1.16)
South West England		1.14***
		(1.10 - 1.18)
North West England		0.99
		(0.96 - 1.02)
Wales		1.12***
		(1.07 - 1.17)
Scotland		0.95**
		(0.91 - 0.98)
Townsend deprivation		0.98***
		(0.98 - 0.99)
Urban (ref: Rural)		0.94***
		(0.92 - 0.96)
Cars per household (ref: None)		
One		1.12***
		(1.08 - 1.16)
Two		1.10***
		(1.06 - 1.14)
Three		1.07**
		(1.02 - 1.12)
Four or more		1.09**
		(1.03 - 1.17)
Observations	217,168	217,168

*** p<0.001, ** p<0.01, * p<0.05

Table S2 – Results of ordinal logistic models between any active travel and fruit and vegetable (FV) consumption among males in UKB (n=195,131)

VARIABLES	Model 1	Model 2
Any active travel (ref: None)	1.37***	1.35***
	(1.34 - 1.39)	(1.33 - 1.37)
Age at baseline		1.03***
		(1.03 - 1.03)
Ethnic group (ref: White British)		
Other white		1.32***
		(1.27 - 1.36)
South Asian		2.24***
		(2.10 - 2.38)
Black		1.50***
		(1.39 - 1.63)
Chinese		1.68***
		(1.41 - 2.00)
Mixed		1.11
		(0.98 - 1.26)
Other		2.23***
		(2.02 - 2.47)
Highest qualification (ref: Degree level)		
A levels/AS levels or equivalent		0.79***
		(0.76 - 0.81)
O levels/GCSEs or equivalent		0.75***
		(0.73 - 0.77)
CSEs or equivalent		0.75***
		(0.72 - 0.78)
NVQ or HND or HNC or equivalent		0.83***
		(0.80 - 0.85)
Other professional qualifications		0.82***
		(0.79 - 0.86)
No qualifications		0.75***
		(0.73 - 0.77)
Occupation class (ref: Higher man / prof)		
Lower managerial / professional		1.03*
		(1.00 - 1.05)
Intermediate occupations		1.05**
		(1.01 - 1.09)
Small employers & own accounts		1.04
		(1.00 - 1.09)
Lower supervisory & technical		1.08***
		(1.03 - 1.12)
Semi-routine occupations		1.00
		(0.96 - 1.04)
Routine occupations		1.04
		(0.99 - 1.08)
Not classified		0.99
		(0.96 - 1.02)
Household income (ref: £<18 000)		
£18,000 to 30,999		1.11***
		(1.08 - 1.14)
£31,000 to 51,999		1.18***
		(1.15 - 1.22)
£52,000 to 100,000		1.26***
		(1.22 - 1.31)
£Greater than 100,000		1.33***
		(1.27 - 1.39)

Household size (ref: One)		
2		1.12***
		(1.09 - 1.15)
3		1.04*
		(1.01 - 1.08)
4		1.03
		(1.00 - 1.07)
5+		1.01
		(0.96 - 1.05)
Region (ref: London)		
North East England		1.02
		(0.98 - 1.05)
Yorkshire and the Humber		0.97
		(0.94 - 1.01)
West Midlands		1.00
		(0.96 - 1.04)
East Midlands		1.03
		(0.99 - 1.07)
South East England		1.00
		(0.96 - 1.04)
South West England		1.02
		(0.98 - 1.06)
North West England		0.94***
		(0.91 - 0.97)
Wales		1.05*
		(1.00 - 1.10)
Scotland		0.83***
		(0.80 - 0.86)
Townsend deprivation		0.99***
		(0.98 - 0.99)
Urban (ref: Rural)		0.96**
		(0.94 - 0.98)
Cars per household (ref: None)		
One		1.11***
		(1.07 - 1.15)
Two		1.02
		(0.98 - 1.06)
Three		0.94**
		(0.89 - 0.98)
Four or more		0.92**
		(0.86 - 0.98)
Observations	195,131	195,131

*** p<0.001, ** p<0.01, * p<0.05

Table S3 – Results of ordinal logistic models between any active travel and red and processed meat (RPM) consumption among females in UKB (n=217,168)

VARIABLES	Model 1	Model 2
Any active travel (ref: None)	0.85***	0.88***
	(0.84 - 0.87)	(0.87 - 0.90)
Age at baseline		1.01***
		(1.01 - 1.01)
Ethnic group (ref: White British)		
Other white		0.99
		(0.95 - 1.02)
South Asian		0.27***
		(0.25 - 0.29)
Black		1.06
		(0.99 - 1.14)
Chinese		2.12***
		(1.83 - 2.45)
Mixed		0.97
		(0.88 - 1.08)
Other		0.91*
		(0.82 - 1.00)
Highest qualification (ref: Degree level)		
A levels/AS levels or equivalent		1.20***
		(1.17 - 1.24)
O levels/GCSEs or equivalent		1.27***
		(1.24 - 1.30)
CSEs or equivalent		1.30***
		(1.24 - 1.35)
NVQ or HND or HNC or equivalent		1.22***
		(1.17 - 1.28)
Other professional qualifications		1.16***
		(1.12 - 1.21)
No qualifications		1.35***
		(1.31 - 1.39)
Occupation class (ref: Higher man / prof)		
Lower managerial / professional		0.96**
		(0.93 - 0.99)
Intermediate occupations		1.06**
		(1.02 - 1.09)
Small employers & own accounts		1.00
		(0.94 - 1.07)
Lower supervisory & technical		1.07
		(0.94 - 1.21)
Semi-routine occupations		1.08***
		(1.04 - 1.13)
Routine occupations		1.20***
		(1.13 - 1.27)
Not classified		1.21***
		(1.17 - 1.25)
Household income (ref: £<18 000)		
£18,000 to 30,999		1.00
		(0.98 - 1.03)
£31,000 to 51,999		0.94***
		(0.92 - 0.97)
£52,000 to 100,000		0.93***
		(0.90 - 0.96)
£Greater than 100,000		0.93**
		(0.88 - 0.97)

Household size (ref: One)		
2		1.45***
		(1.41 - 1.48)
3		1.57***
		(1.52 - 1.63)
4		1.79***
		(1.73 - 1.86)
5+		1.92***
		(1.83 - 2.01)
Region (ref: London)		
North East England		0.97
		(0.94 - 1.01)
Yorkshire and the Humber		1.01
		(0.98 - 1.05)
West Midlands		0.97
		(0.93 - 1.00)
East Midlands		1.00
		(0.96 - 1.04)
South East England		1.03
		(0.99 - 1.07)
South West England		0.94***
		(0.90 - 0.97)
North West England		1.15***
		(1.11 - 1.19)
Wales		0.89***
		(0.85 - 0.94)
Scotland		1.20***
		(1.15 - 1.24)
Townsend deprivation		1.00
		(1.00 - 1.00)
Urban (ref: Rural)		0.96**
		(0.94 - 0.98)
Cars per household (ref: None)		
One		1.04*
		(1.01 - 1.08)
Two		1.17***
		(1.12 - 1.21)
Three		1.24***
		(1.18 - 1.30)
Four or more		1.29***
		(1.20 - 1.38)
Observations	217,168	217,168

*** p<0.001, ** p<0.01, * p<0.05

Table S4 – Results of ordinal logistic models between any active travel and red and processed meat (RPM) consumption among males in UKB (n=195,131)

VARIABLES	Model 1	Model 2
Any active travel (ref: None)	0.87***	0.89***
	(0.85 - 0.88)	(0.87 - 0.91)
Age at baseline		1.00
		(1.00 - 1.00)
Ethnic group (ref: White British)		
Other white		1.01
		(0.97 - 1.05)
South Asian		0.26***
		(0.25 - 0.28)
Black		0.82***
		(0.76 - 0.90)
Chinese		1.33**
		(1.09 - 1.61)
Mixed		1.08
		(0.94 - 1.23)
Other		0.74***
		(0.66 - 0.82)
Highest qualification (ref: Degree level)		
A levels/AS levels or equivalent		1.20***
		(1.16 - 1.23)
O levels/GCSEs or equivalent		1.22***
		(1.19 - 1.26)
CSEs or equivalent		1.22***
		(1.17 - 1.27)
NVQ or HND or HNC or equivalent		1.20***
		(1.16 - 1.24)
Other professional qualifications		1.09***
		(1.04 - 1.14)
No qualifications		1.16***
		(1.13 - 1.20)
Occupation class (ref: Higher man / prof)		
Lower managerial / professional		0.95***
		(0.92 - 0.98)
Intermediate occupations		1.01
		(0.97 - 1.05)
Small employers & own accounts		1.19***
		(1.14 - 1.25)
Lower supervisory & technical		1.18***
		(1.13 - 1.23)
Semi-routine occupations		1.20***
		(1.15 - 1.26)
Routine occupations		1.28***
		(1.22 - 1.34)
Not classified		1.14***
		(1.11 - 1.18)
Household income (ref: £<18 000)		
£18,000 to 30,999		0.96*
		(0.94 - 0.99)
£31,000 to 51,999		0.97
		(0.94 - 1.01)
£52,000 to 100,000		0.91***
		(0.88 - 0.95)
£Greater than 100,000		0.89***
		(0.85 - 0.94)

Household size (ref: One)		
2		1.06***
		(1.03 - 1.09)
3		1.17***
		(1.13 - 1.21)
4		1.25***
		(1.20 - 1.29)
5+		1.35***
		(1.29 - 1.42)
Region (ref: London)		
North East England		0.99
		(0.95 - 1.03)
Yorkshire and the Humber		1.02
		(0.98 - 1.06)
West Midlands		1.02
		(0.98 - 1.07)
East Midlands		0.97
		(0.93 - 1.01)
South East England		1.03
		(0.99 - 1.07)
South West England		0.96*
		(0.92 - 1.00)
North West England		1.15***
		(1.11 - 1.19)
Wales		0.91***
		(0.87 - 0.96)
Scotland		1.16***
		(1.11 - 1.21)
Townsend deprivation		1.01***
		(1.01 - 1.01)
Urban (ref: Rural)		0.99
		(0.97 - 1.02)
Cars per household (ref: None)		
One		0.98
		(0.94 - 1.02)
Two		1.08***
		(1.04 - 1.13)
Three		1.23***
		(1.17 - 1.30)
Four or more		1.30***
		(1.21 - 1.39)
Observations	195,131	195,131

*** p<0.001, ** p<0.01, * p<0.05

Figure S2 – Associations between travel and FV consumption (Model 2, Table 4)

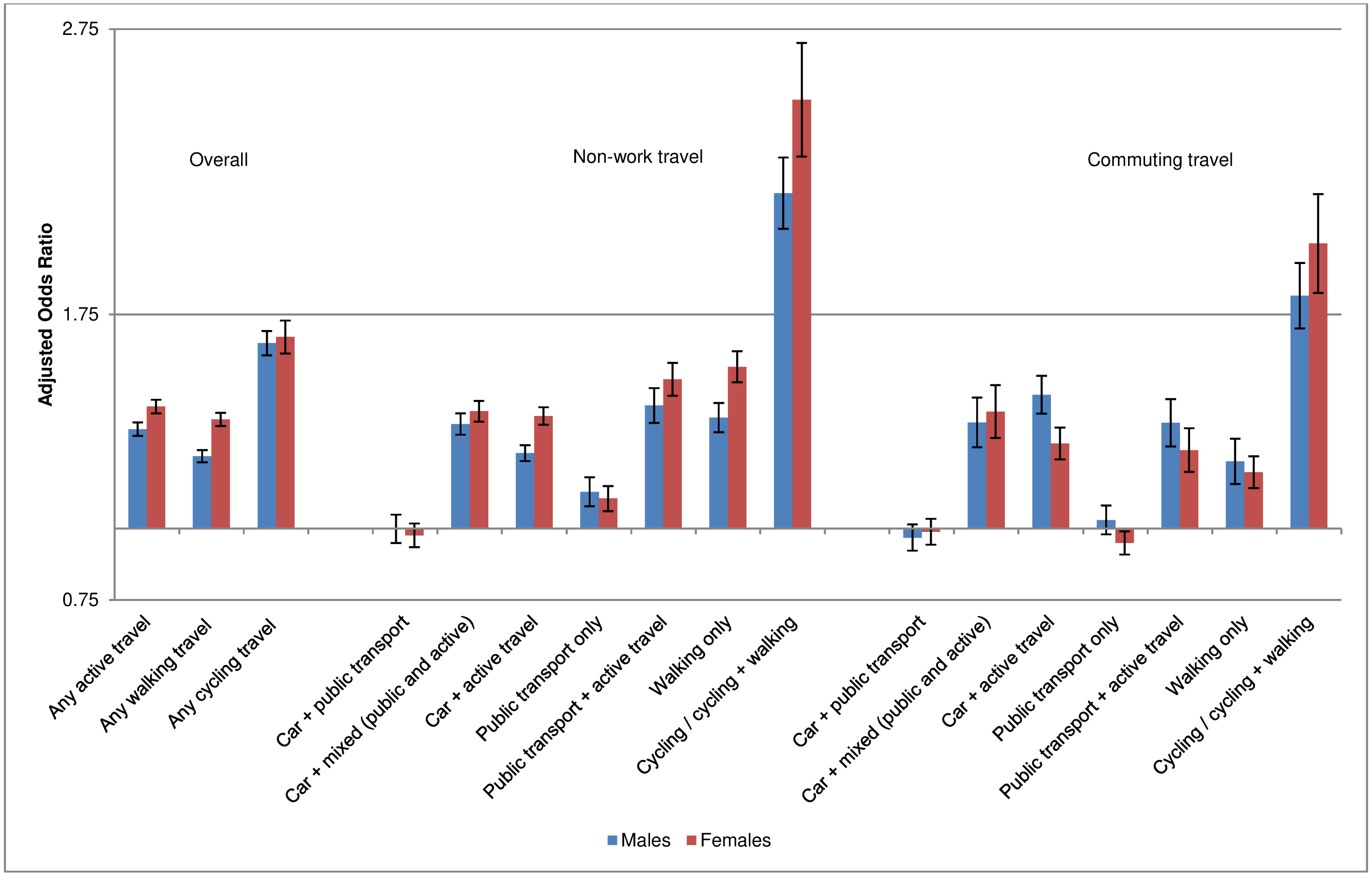

Whiskers = 95% confidence interval

Figure S3 – Associations between travel and RPM consumption (Model 2, Table 5)

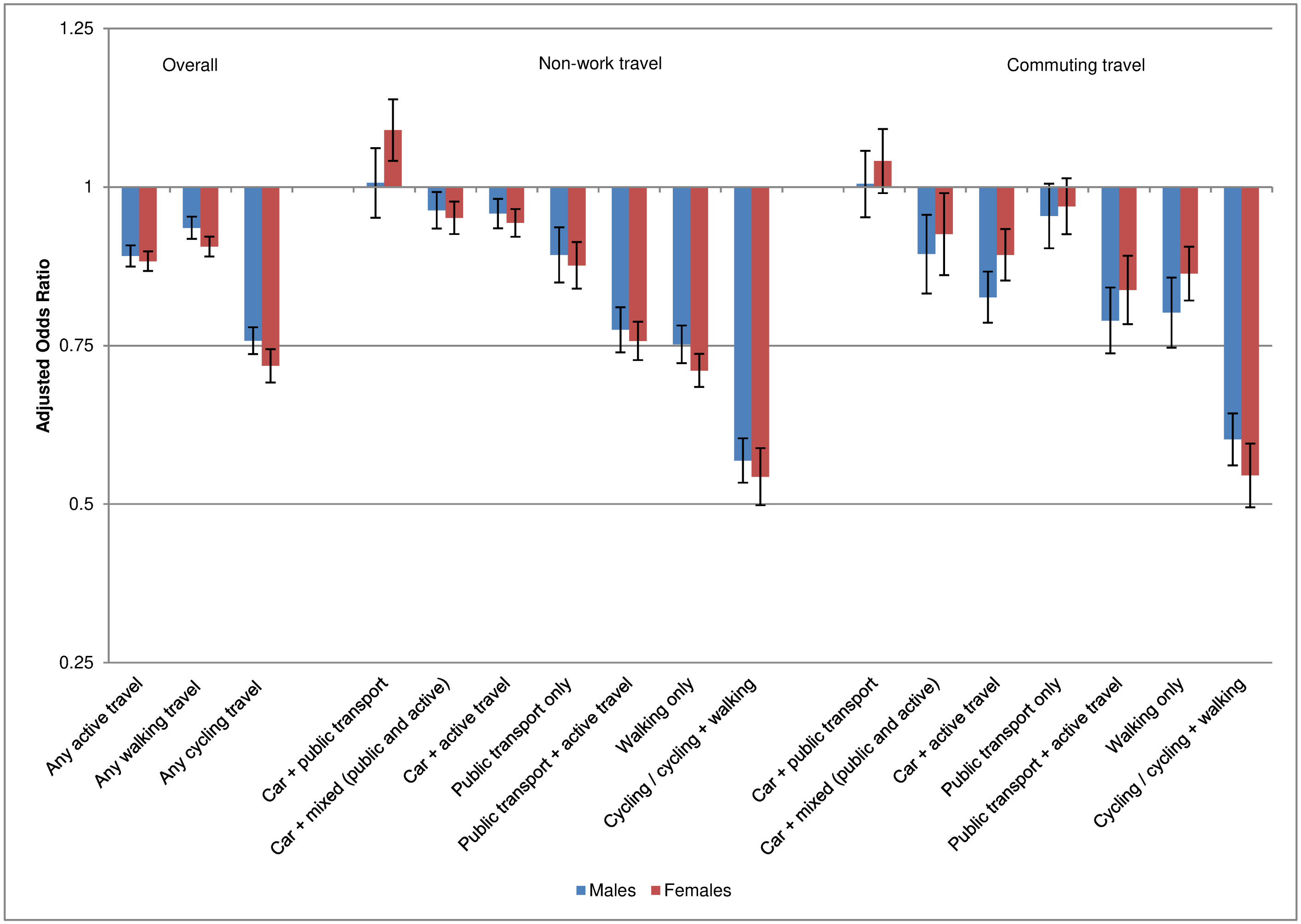

Whiskers = 95% confidence interval

Table S5 – Results of generalized ordered logit models between measures of active travel and FV consumption, stratified by gender in UKB (n=412,299)

TRAVEL VARIABLES	Males (n=195,131) Model 2 [a] 1 v. 2 + 3 [b]	Males (n=195,131) Model 2 [a] 1 + 2 v. 3	Females (n=217,168) Model 2 [a] 1 v. 2 + 3 [b]	Females (n=217,168) Model 2 [a] 1 + 2 v. 3
Any active travel (ref: No)	1.37*** (1.35 - 1.40)	1.32*** (1.30 - 1.35)	1.53*** (1.49 - 1.56)	1.38*** (1.35 - 1.40)
Any walking (ref: No)	1.28*** (1.26 - 1.31)	1.23*** (1.20 - 1.25)	1.47*** (1.44 - 1.51)	1.34*** (1.32 - 1.36)
Any cycling (ref: No)	1.71*** (1.66 - 1.76)	1.61*** (1.57 - 1.66)	1.93*** (1.84 - 2.04)	1.60*** (1.55 - 1.66)
Non-work travel [b] (ref: Car only)				
Car + public transport	1.00 (0.95 - 1.05)	1.00 (0.95 - 1.05)	0.98 (0.94 - 1.02)	0.98 (0.94 - 1.02)
Car + mixed (public and active)	1.42*** (1.38 - 1.47)	1.32*** (1.28 - 1.36)	1.55*** (1.50 - 1.61)	1.36*** (1.32 - 1.39)
Car + active travel	1.29*** (1.26 - 1.32)	1.24*** (1.21 - 1.27)	1.49*** (1.45 - 1.54)	1.35*** (1.32 - 1.38)
Public transport only	1.14*** (1.09 - 1.19)	1.14*** (1.09 - 1.19)	1.11*** (1.07 - 1.16)	1.11*** (1.07 - 1.16)
Public transport + active travel	1.47*** (1.40 - 1.54)	1.39*** (1.33 - 1.46)	1.66*** (1.58 - 1.74)	1.45*** (1.40 - 1.51)
Walking only	1.39*** (1.34 - 1.44)	1.39*** (1.34 - 1.44)	1.62*** (1.55 - 1.69)	1.53*** (1.48 - 1.59)
Cycling / cycling + walking	2.27*** (2.11 - 2.44)	2.10*** (1.97 - 2.23)	2.84*** (2.50 - 3.21)	2.39*** (2.21 - 2.60)
Commuting travel [c] (ref: Car only)				
Car + public transport	0.97 (0.92 - 1.02)	0.97 (0.92 - 1.02)	0.99 (0.95 - 1.03)	0.99 (0.95 - 1.03)
Car + mixed (public and active)	1.38*** (1.29 - 1.47)	1.38*** (1.29 - 1.47)	1.53*** (1.39 - 1.67)	1.37*** (1.28 - 1.47)
Car + active travel	1.47*** (1.41 - 1.54)	1.47*** (1.41 - 1.54)	1.35*** (1.28 - 1.43)	1.28*** (1.22 - 1.33)
Public transport only	1.03 (0.99 - 1.09)	1.03 (0.99 - 1.09)	0.95* (0.91 - 0.99)	0.95* (0.91 - 0.99)
Public transport + active travel	1.37*** (1.29 - 1.46)	1.37*** (1.29 - 1.46)	1.27*** (1.20 - 1.35)	1.27*** (1.20 - 1.35)
Walking only	1.19*** (1.11 - 1.28)	1.29*** (1.20 - 1.38)	1.20*** (1.14 - 1.25)	1.20*** (1.14 - 1.25)
Cycling / cycling + walking	1.82*** (1.71 - 1.94)	1.82*** (1.71 - 1.94)	2.00*** (1.84 - 2.18)	2.00*** (1.84 - 2.18)

*** p<0.001, ** p<0.01, * p<0.05

a) Adjusted for: age, ethnic group, education, occupational class, household income, household size, number of cars, assessment centre location, population density, Townsend score

b) Shading and boxes indicate variables with different relationships across the levels of the outcome variable: 1 = <3 portions FV, 2 = 3-<5 portions FV, 3 = 5+ portions FV

Table S6 – Results of generalized ordered logit models between measures of active travel and RPM consumption, stratified by gender in UKB (n=412,299)

TRAVEL VARIABLES	Males (n=195,131)		Females (n=217,168)	
	Model 2 [a]		Model 2 [a]	
	1 v. 2 + 3 [b]	1 + 2 v. 3	1 v. 2 + 3 [b]	1 + 2 v. 3
Any active travel (ref: No)	0.72*** (0.68 - 0.76)	0.90*** (0.89 - 0.92)	0.79*** (0.76 - 0.81)	0.90*** (0.89 - 0.92)
Any walking (ref: No)	0.86*** (0.81 - 0.90)	0.94*** (0.92 - 0.96)	0.83*** (0.80 - 0.86)	0.92*** (0.91 - 0.94)
Any cycling (ref: No)	0.56*** (0.52 - 0.59)	0.78*** (0.76 - 0.81)	0.63*** (0.60 - 0.67)	0.77*** (0.75 - 0.80)
Non-work travel [b] (ref: Car only)				
Car + public transport	1.01 (0.95 - 1.06)	1.01 (0.95 - 1.06)	1.10*** (1.05 - 1.15)	1.10*** (1.05 - 1.15)
Car + mixed (public and active)	0.78*** (0.72 - 0.84)	0.97 (0.94 - 1.00)	0.87*** (0.83 - 0.92)	0.97* (0.94 - 0.99)
Car + active travel	0.83*** (0.77 - 0.89)	0.96** (0.94 - 0.99)	0.86*** (0.82 - 0.90)	0.96*** (0.93 - 0.98)
Public transport only	0.75*** (0.67 - 0.84)	0.89*** (0.85 - 0.94)	0.87*** (0.83 - 0.90)	0.87*** (0.83 - 0.90)
Public transport + active travel	0.60*** (0.54 - 0.66)	0.79*** (0.75 - 0.82)	0.67*** (0.62 - 0.71)	0.79*** (0.75 - 0.82)
Walking only	0.62*** (0.56 - 0.68)	0.76*** (0.73 - 0.79)	0.64*** (0.60 - 0.68)	0.73*** (0.70 - 0.76)
Cycling / cycling + walking	0.37*** (0.33 - 0.41)	0.61*** (0.58 - 0.65)	0.49*** (0.44 - 0.55)	0.60*** (0.55 - 0.66)
Commuting travel [c] (ref: Car only)				
Car + public transport	1.00 (0.95 - 1.05)	1.00 (0.95 - 1.05)	0.95 (0.87 - 1.04)	1.06* (1.01 - 1.11)
Car + mixed (public and active)	0.58*** (0.51 - 0.67)	0.93* (0.87 - 0.99)	0.77*** (0.69 - 0.85)	0.98 (0.91 - 1.06)
Car + active travel	0.65*** (0.58 - 0.73)	0.84*** (0.80 - 0.88)	0.81*** (0.75 - 0.87)	0.92*** (0.88 - 0.96)
Public transport only	0.82*** (0.73 - 0.92)	0.96 (0.91 - 1.01)	0.96 (0.92 - 1.01)	0.96 (0.92 - 1.01)
Public transport + active travel	0.57*** (0.50 - 0.65)	0.82*** (0.77 - 0.88)	0.73*** (0.66 - 0.80)	0.89*** (0.83 - 0.95)
Walking only	0.65*** (0.56 - 0.75)	0.82*** (0.76 - 0.87)	0.79*** (0.73 - 0.86)	0.88*** (0.84 - 0.93)
Cycling / cycling + walking	0.39*** (0.35 - 0.44)	0.65*** (0.61 - 0.70)	0.46*** (0.41 - 0.52)	0.65*** (0.59 - 0.71)

*** p<0.001, ** p<0.01, * p<0.05

a) Adjusted for: age, ethnic group, education, occupational class, household income, household size, number of cars, assessment centre location, population density, Townsend score

b) Shading and boxes indicate variables with different relationships across the levels of the outcome variable: 1 = 0 g RPM per day; 2 = >0-70 g RPM per day; 3 = >70 g RPM per day

Table S7 – Sensitivity analysis: results of ordinal logistic models between any active travel and FV consumption among females in UKB (n=95,475)

VARIABLES	Model 1	Model 2	Model 3
Any active travel (ref: None)	1.42***	1.42***	1.35***
	(1.38 - 1.45)	(1.38 - 1.45)	(1.32 - 1.39)
Age at baseline		1.05***	1.05***
		(1.04 - 1.05)	(1.04 - 1.05)
Ethnic group (ref: White British)			
Other white		1.39***	1.38***
		(1.32 - 1.46)	(1.31 - 1.45)
South Asian		2.05***	2.09***
		(1.81 - 2.33)	(1.84 - 2.37)
Black		1.60***	1.58***
		(1.43 - 1.80)	(1.41 - 1.76)
Chinese		1.54***	1.55***
		(1.23 - 1.94)	(1.23 - 1.95)
Mixed		1.06	1.05
		(0.91 - 1.22)	(0.90 - 1.21)
Other		2.13***	2.16***
		(1.82 - 2.48)	(1.85 - 2.53)
Highest qualification (ref: Degree level)			
A levels/AS levels or equivalent		0.87***	0.87***
		(0.84 - 0.90)	(0.84 - 0.91)
O levels/GCSEs or equivalent		0.78***	0.79***
		(0.75 - 0.81)	(0.76 - 0.81)
CSEs or equivalent		0.71***	0.71***
		(0.66 - 0.75)	(0.67 - 0.76)
NVQ or HND or HNC or equivalent		0.82***	0.82***
		(0.77 - 0.88)	(0.76 - 0.88)
Other professional qualifications		0.92**	0.91**
		(0.87 - 0.97)	(0.86 - 0.96)
No qualifications		0.68***	0.68***
		(0.64 - 0.71)	(0.64 - 0.72)
Occupation class (ref: Higher man / prof)			
Lower managerial / professional		1.07***	1.05**
		(1.03 - 1.11)	(1.01 - 1.10)
Intermediate occupations		1.01	1.00
		(0.97 - 1.06)	(0.96 - 1.05)
Small employers & own accounts		1.12**	1.08
		(1.04 - 1.22)	(0.99 - 1.17)
Lower supervisory & technical		1.14	1.04
		(0.96 - 1.37)	(0.87 - 1.25)
Semi-routine occupations		1.10**	1.06
		(1.04 - 1.16)	(1.00 - 1.12)
Routine occupations		0.96	0.89*
		(0.86 - 1.07)	(0.80 - 0.99)
Not classified		1.04	0.99
		(0.99 - 1.09)	(0.95 - 1.04)
Household income (ref: £<18 000)			
£18,000 to 30,999		1.11***	1.11***
		(1.06 - 1.15)	(1.07 - 1.16)
£31,000 to 51,999		1.23***	1.25***
		(1.18 - 1.28)	(1.19 - 1.30)
£52,000 to 100,000		1.31***	1.32***
		(1.25 - 1.37)	(1.26 - 1.39)
£Greater than 100,000		1.34***	1.33***
		(1.25 - 1.43)	(1.24 - 1.42)

Household size (ref: One)			
2		0.94***	0.93***
		(0.90 - 0.97)	(0.90 - 0.97)
3		0.90***	0.90***
		(0.86 - 0.94)	(0.86 - 0.94)
4		0.84***	0.84***
		(0.80 - 0.89)	(0.80 - 0.88)
5+		0.84***	0.82***
		(0.79 - 0.90)	(0.77 - 0.88)
Region (ref: London)			
North East England		1.11***	1.14***
		(1.05 - 1.16)	(1.08 - 1.20)
Yorkshire and the Humber		1.06**	1.07**
		(1.01 - 1.11)	(1.02 - 1.12)
West Midlands		1.05	1.07**
		(1.00 - 1.11)	(1.02 - 1.13)
East Midlands		1.15***	1.17***
		(1.09 - 1.22)	(1.11 - 1.25)
South East England		1.18***	1.21***
		(1.12 - 1.24)	(1.15 - 1.27)
South West England		1.19***	1.21***
		(1.14 - 1.26)	(1.16 - 1.28)
North West England		1.07**	1.09***
		(1.02 - 1.12)	(1.04 - 1.14)
Wales		1.16***	1.20***
		(1.08 - 1.26)	(1.11 - 1.29)
Scotland		1.09**	1.12***
		(1.03 - 1.16)	(1.05 - 1.19)
Townsend deprivation		0.99***	0.99***
		(0.99 - 0.99)	(0.99 - 1.00)
Urban (ref: Rural)		0.93***	0.95**
		(0.90 - 0.97)	(0.91 - 0.98)
Cars per household (ref: None)			
One		1.00	1.01
		(0.95 - 1.06)	(0.95 - 1.06)
Two		0.96	0.95
		(0.90 - 1.02)	(0.90 - 1.01)
Three		0.92*	0.91*
		(0.86 - 0.99)	(0.85 - 0.98)
Four or more		0.96	0.95
		(0.87 - 1.06)	(0.86 - 1.05)
Meets physical activity guideline (ref: No)			1.65***
			(1.61 - 1.69)
Total energy intake (kcal)			1.00***
			(1.00 - 1.00)
Observations	95,475	95,475	95,475

*** p<0.001, ** p<0.01, * p<0.05

Table S8 – Sensitivity analysis: results of ordinal logistic models between any active travel and FV consumption among males in UKB (n=83,213)

VARIABLES	Model 1	Model 2	Model 3
Any active travel (ref: None)	1.38***	1.35***	1.28***
	(1.34 - 1.41)	(1.32 - 1.39)	(1.24 - 1.31)
Age at baseline		1.03***	1.03***
		(1.03 - 1.03)	(1.03 - 1.03)
Ethnic group (ref: White British)			
Other white		1.28***	1.28***
		(1.21 - 1.35)	(1.21 - 1.35)
South Asian		2.02***	2.13***
		(1.81 - 2.25)	(1.91 - 2.38)
Black		1.42***	1.44***
		(1.25 - 1.62)	(1.26 - 1.64)
Chinese		1.59**	1.65***
		(1.20 - 2.12)	(1.24 - 2.20)
Mixed		1.14	1.12
		(0.94 - 1.37)	(0.93 - 1.35)
Other		2.04***	2.10***
		(1.72 - 2.43)	(1.76 - 2.49)
Highest qualification (ref: Degree level)			
A levels/AS levels or equivalent		0.78***	0.79***
		(0.75 - 0.82)	(0.76 - 0.82)
O levels/GCSEs or equivalent		0.76***	0.76***
		(0.73 - 0.79)	(0.73 - 0.79)
CSEs or equivalent		0.74***	0.73***
		(0.69 - 0.79)	(0.68 - 0.78)
NVQ or HND or HNC or equivalent		0.84***	0.83***
		(0.80 - 0.89)	(0.79 - 0.87)
Other professional qualifications		0.87***	0.86***
		(0.82 - 0.93)	(0.80 - 0.92)
No qualifications		0.79***	0.78***
		(0.75 - 0.84)	(0.74 - 0.83)
Occupation class (ref: Higher man / prof)			
Lower managerial / professional		1.03	1.02
		(1.00 - 1.07)	(0.99 - 1.06)
Intermediate occupations		1.06*	1.05
		(1.01 - 1.12)	(1.00 - 1.11)
Small employers & own accounts		1.08	0.99
		(1.00 - 1.16)	(0.92 - 1.07)
Lower supervisory & technical		1.13***	1.02
		(1.06 - 1.21)	(0.95 - 1.09)
Semi-routine occupations		0.99	0.92*
		(0.92 - 1.06)	(0.86 - 0.98)
Routine occupations		1.04	0.94
		(0.97 - 1.13)	(0.87 - 1.01)
Not classified		0.96*	0.92***
		(0.92 - 1.00)	(0.88 - 0.95)
Household income (ref: £<18 000)			
£18,000 to 30,999		1.09***	1.08**
		(1.04 - 1.14)	(1.03 - 1.13)
£31,000 to 51,999		1.20***	1.20***
		(1.15 - 1.26)	(1.14 - 1.25)
£52,000 to 100,000		1.28***	1.29***
		(1.21 - 1.35)	(1.23 - 1.37)
£Greater than 100,000		1.35***	1.35***
		(1.26 - 1.45)	(1.27 - 1.45)

Household size (ref: One)			
2		1.13***	1.12***
		(1.08 - 1.18)	(1.07 - 1.17)
3		1.02	1.02
		(0.97 - 1.07)	(0.97 - 1.07)
4		1.02	1.01
		(0.96 - 1.07)	(0.96 - 1.06)
5+		0.99	0.97
		(0.93 - 1.06)	(0.91 - 1.04)
Region (ref: London)			
North East England		1.05	1.06*
		(1.00 - 1.11)	(1.01 - 1.12)
Yorkshire and the Humber		1.03	1.03
		(0.99 - 1.08)	(0.99 - 1.08)
West Midlands		0.98	0.98
		(0.93 - 1.03)	(0.93 - 1.04)
East Midlands		1.11**	1.11**
		(1.04 - 1.18)	(1.04 - 1.18)
South East England		1.04	1.06*
		(0.98 - 1.10)	(1.01 - 1.12)
South West England		1.07*	1.08**
		(1.02 - 1.13)	(1.02 - 1.13)
North West England		1.00	1.00
		(0.95 - 1.05)	(0.96 - 1.05)
Wales		1.12**	1.14**
		(1.03 - 1.21)	(1.05 - 1.23)
Scotland		0.91**	0.93*
		(0.85 - 0.97)	(0.87 - 0.99)
Townsend deprivation		1.00	1.00
		(0.99 - 1.00)	(0.99 - 1.00)
Urban (ref: Rural)		0.95**	0.96*
		(0.92 - 0.99)	(0.93 - 1.00)
Cars per household (ref: None)			
One		1.01	1.00
		(0.95 - 1.07)	(0.94 - 1.06)
Two		0.92**	0.89***
		(0.86 - 0.98)	(0.83 - 0.95)
Three		0.84***	0.81***
		(0.78 - 0.91)	(0.75 - 0.87)
Four or more		0.81***	0.78***
		(0.73 - 0.90)	(0.71 - 0.87)
Meets physical activity guideline (ref: No)			1.69***
			(1.64 - 1.73)
Total energy intake (kcal)			1.00***
			(1.00 - 1.00)
Observations	83,213	83,213	83,213

*** p<0.001, ** p<0.01, * p<0.05

Table S9 – Sensitivity analysis: results of ordinal logistic models between any active travel and RPM consumption among females in UKB (n=95,475)

VARIABLES	Model 1	Model 2	Model 3
Any active travel (ref: None)	0.86***	0.89***	0.90***
	(0.83 - 0.88)	(0.87 - 0.91)	(0.88 - 0.92)
Age at baseline		1.01***	1.01***
		(1.01 - 1.01)	(1.01 - 1.01)
Ethnic group (ref: White British)			
Other white		1.02	1.02
		(0.97 - 1.07)	(0.97 - 1.07)
South Asian		0.32***	0.33***
		(0.28 - 0.36)	(0.29 - 0.37)
Black		1.09	1.09
		(0.98 - 1.23)	(0.97 - 1.22)
Chinese		1.83***	1.87***
		(1.46 - 2.30)	(1.49 - 2.35)
Mixed		1.00	1.01
		(0.86 - 1.17)	(0.86 - 1.18)
Other		0.75***	0.75***
		(0.65 - 0.88)	(0.65 - 0.87)
Highest qualification (ref: Degree level)			
A levels/AS levels or equivalent		1.21***	1.22***
		(1.16 - 1.26)	(1.18 - 1.27)
O levels/GCSEs or equivalent		1.28***	1.30***
		(1.23 - 1.32)	(1.26 - 1.35)
CSEs or equivalent		1.29***	1.33***
		(1.20 - 1.38)	(1.24 - 1.42)
NVQ or HND or HNC or equivalent		1.20***	1.24***
		(1.11 - 1.29)	(1.15 - 1.33)
Other professional qualifications		1.15***	1.16***
		(1.08 - 1.21)	(1.10 - 1.23)
No qualifications		1.29***	1.34***
		(1.22 - 1.37)	(1.27 - 1.42)
Occupation class (ref: Higher man / prof)			
Lower managerial / professional		0.96	0.96*
		(0.92 - 1.00)	(0.92 - 1.00)
Intermediate occupations		1.06*	1.06*
		(1.01 - 1.11)	(1.01 - 1.11)
Small employers & own accounts		1.00	1.01
		(0.92 - 1.09)	(0.93 - 1.10)
Lower supervisory & technical		1.04	1.07
		(0.86 - 1.26)	(0.89 - 1.29)
Semi-routine occupations		1.07*	1.07*
		(1.01 - 1.14)	(1.01 - 1.14)
Routine occupations		1.19**	1.19**
		(1.06 - 1.33)	(1.07 - 1.34)
Not classified		1.16***	1.16***
		(1.11 - 1.21)	(1.11 - 1.22)
Household income (ref: £<18 000)			
£18,000 to 30,999		1.04	1.05*
		(1.00 - 1.08)	(1.01 - 1.09)
£31,000 to 51,999		0.99	1.00
		(0.95 - 1.03)	(0.96 - 1.05)
£52,000 to 100,000		0.99	1.01
		(0.94 - 1.04)	(0.96 - 1.06)
£Greater than 100,000		0.99	1.04
		(0.93 - 1.06)	(0.97 - 1.11)

Household size (ref: One)			
2		1.41***	1.41***
		(1.36 - 1.47)	(1.35 - 1.46)
3		1.52***	1.50***
		(1.45 - 1.60)	(1.43 - 1.58)
4		1.77***	1.74***
		(1.68 - 1.87)	(1.65 - 1.83)
5+		1.91***	1.86***
		(1.78 - 2.05)	(1.74 - 2.00)
Region (ref: London)			
North East England		0.95*	0.94*
		(0.90 - 1.00)	(0.89 - 0.99)
Yorkshire and the Humber		0.99	0.98
		(0.95 - 1.04)	(0.94 - 1.03)
West Midlands		0.93**	0.92**
		(0.88 - 0.98)	(0.87 - 0.97)
East Midlands		0.97	0.95
		(0.91 - 1.03)	(0.90 - 1.02)
South East England		1.03	1.02
		(0.98 - 1.09)	(0.96 - 1.08)
South West England		0.91***	0.90***
		(0.87 - 0.96)	(0.86 - 0.95)
North West England		1.07**	1.06*
		(1.02 - 1.12)	(1.01 - 1.11)
Wales		0.85***	0.84***
		(0.78 - 0.92)	(0.77 - 0.91)
Scotland		1.08*	1.06
		(1.02 - 1.15)	(1.00 - 1.13)
Townsend deprivation		0.99***	0.99***
		(0.98 - 0.99)	(0.98 - 0.99)
Urban (ref: Rural)		0.97	0.96*
		(0.93 - 1.01)	(0.93 - 1.00)
Cars per household (ref: None)			
One		1.19***	1.19***
		(1.12 - 1.26)	(1.13 - 1.26)
Two		1.34***	1.36***
		(1.26 - 1.43)	(1.27 - 1.45)
Three		1.42***	1.45***
		(1.32 - 1.54)	(1.35 - 1.57)
Four or more		1.36***	1.40***
		(1.23 - 1.51)	(1.26 - 1.55)
Meets physical activity guideline (ref: No)			0.81***
			(0.79 - 0.83)
Total energy intake (kcal)			1.00***
			(1.00 - 1.00)
Observations	95,475	95,475	95,475

*** p<0.001, ** p<0.01, * p<0.05

Table S10 – Sensitivity analysis: results of ordinal logistic models between any active travel and RPM consumption among males in UKB (n=83,213)

VARIABLES	Model 1	Model 2	Model 3
Any active travel (ref: None)	0.86***	0.89***	0.89***
	(0.84 - 0.88)	(0.86 - 0.91)	(0.87 - 0.92)
Age at baseline		1.00*	1.00***
		(1.00 - 1.00)	(1.00 - 1.01)
Ethnic group (ref: White British)			
Other white		0.99	0.99
		(0.93 - 1.05)	(0.94 - 1.05)
South Asian		0.27***	0.28***
		(0.24 - 0.30)	(0.25 - 0.31)
Black		0.95	0.99
		(0.82 - 1.09)	(0.86 - 1.15)
Chinese		1.36	1.42*
		(1.00 - 1.86)	(1.03 - 1.94)
Mixed		1.17	1.19
		(0.96 - 1.44)	(0.97 - 1.46)
Other		0.59***	0.60***
		(0.49 - 0.70)	(0.50 - 0.71)
Highest qualification (ref: Degree level)			
A levels/AS levels or equivalent		1.16***	1.17***
		(1.11 - 1.22)	(1.11 - 1.22)
O levels/GCSEs or equivalent		1.20***	1.22***
		(1.15 - 1.25)	(1.17 - 1.27)
CSEs or equivalent		1.19***	1.22***
		(1.11 - 1.28)	(1.14 - 1.32)
NVQ or HND or HNC or equivalent		1.20***	1.23***
		(1.13 - 1.27)	(1.16 - 1.30)
Other professional qualifications		1.01	1.03
		(0.94 - 1.09)	(0.96 - 1.11)
No qualifications		1.10**	1.13***
		(1.04 - 1.17)	(1.07 - 1.20)
Occupation class (ref: Higher man / prof)			
Lower managerial / professional		0.97	0.96*
		(0.93 - 1.01)	(0.92 - 1.00)
Intermediate occupations		1.02	1.02
		(0.97 - 1.08)	(0.97 - 1.08)
Small employers & own accounts		1.07	1.06
		(0.99 - 1.16)	(0.98 - 1.15)
Lower supervisory & technical		1.13**	1.14***
		(1.05 - 1.22)	(1.05 - 1.22)
Semi-routine occupations		1.19***	1.18***
		(1.10 - 1.28)	(1.10 - 1.27)
Routine occupations		1.22***	1.23***
		(1.12 - 1.33)	(1.13 - 1.33)
Not classified		1.13***	1.13***
		(1.08 - 1.18)	(1.08 - 1.18)
Household income (ref: £<18 000)			
£18,000 to 30,999		0.91***	0.92***
		(0.87 - 0.96)	(0.87 - 0.96)
£31,000 to 51,999		0.96	0.97
		(0.91 - 1.01)	(0.92 - 1.02)
£52,000 to 100,000		0.90***	0.92**
		(0.85 - 0.96)	(0.87 - 0.98)
£Greater than 100,000		0.85***	0.88***
		(0.79 - 0.91)	(0.82 - 0.95)

Household size (ref: One)			
2		1.04	1.05*
		(1.00 - 1.09)	(1.00 - 1.09)
3		1.16***	1.15***
		(1.10 - 1.22)	(1.09 - 1.22)
4		1.27***	1.26***
		(1.21 - 1.35)	(1.19 - 1.33)
5+		1.31***	1.28***
		(1.22 - 1.41)	(1.19 - 1.38)
Region (ref: London)			
North East England		0.94*	0.94*
		(0.89 - 1.00)	(0.89 - 1.00)
Yorkshire and the Humber		0.94**	0.94*
		(0.89 - 0.98)	(0.90 - 0.99)
West Midlands		0.91**	0.91**
		(0.86 - 0.97)	(0.86 - 0.97)
East Midlands		0.86***	0.86***
		(0.80 - 0.92)	(0.80 - 0.92)
South East England		1.00	1.00
		(0.94 - 1.06)	(0.94 - 1.06)
South West England		0.91**	0.91**
		(0.86 - 0.97)	(0.86 - 0.96)
North West England		1.06*	1.07*
		(1.01 - 1.12)	(1.01 - 1.12)
Wales		0.80***	0.80***
		(0.73 - 0.87)	(0.73 - 0.87)
Scotland		1.05	1.04
		(0.98 - 1.12)	(0.97 - 1.12)
Townsend deprivation		1.00	1.00
		(0.99 - 1.00)	(0.99 - 1.00)
Urban (ref: Rural)		0.97	0.97
		(0.94 - 1.01)	(0.93 - 1.01)
Cars per household (ref: None)			
One		1.11**	1.11**
		(1.04 - 1.18)	(1.04 - 1.19)
Two		1.27***	1.28***
		(1.18 - 1.36)	(1.19 - 1.37)
Three		1.44***	1.46***
		(1.33 - 1.57)	(1.34 - 1.59)
Four or more		1.40***	1.43***
		(1.26 - 1.57)	(1.28 - 1.60)
Meets physical activity guideline (ref: No)			0.84***
			(0.82 - 0.87)
Total energy intake (kcal)			1.00***
			(1.00 - 1.00)
Observations	83,213	83,213	83,213

*** p<0.001, ** p<0.01, * p<0.05
