# Supplementary material for: Associations between active travel and diet: cross-sectional evidence on healthy, low-carbon behaviours from UK Biobank
Source: BMJ Open. 2019 Sep 3;9(8):e030741. doi: 10.1136/bmjopen-2019-030741 (PMC6731823; doi:10.1136/bmjopen-2019-030741)
Supplement: Author's manuscript [file bmjopen-2019-030741.draft_revisions.pdf]

BMJ Open

BMJ Open is committed to open peer review. As part of this commitment we make the peer review history of every article we publish publicly available.

When an article is published we post the peer reviewers' comments and the authors' responses online. We also post the versions of the paper that were used during peer review. These are the versions that the peer review comments apply to.

The versions of the paper that follow are the versions that were submitted during the peer review process. They are not the versions of record or the final published versions. They should not be cited or distributed as the published version of this manuscript.

BMJ Open is an open access journal and the full, final, typeset and author-corrected version of record of the manuscript is available on our site with no access controls, subscription charges or pay-per-view fees (http://bmjopen.bmj.com).

If you have any questions on BMJ Open's open peer review process please email info.bmjopen@bmj.com

BMJ Open

Associations between active travel and diet: cross-sectional evidence on healthy, low-carbon behaviours from UK Biobank

Journal:	*BMJ Open*
Manuscript ID	bmjopen-2019-030741
Article Type:	Research
Date Submitted by the Author:	29-Mar-2019

Complete List of Authors:	Smith, Michaela; University of York<br>Boehnke, Jan Rasmus; University of Dundee, School of Nursing and Health Sciences<br>Graham, Hilary; University of York<br>White, Piran; University of York<br>Prady, Stephanie; University of York

Keywords:	PUBLIC HEALTH, PREVENTIVE MEDICINE, EPIDEMIOLOGY, ACTIVE TRAVEL, DIET

SCHOLARONE™
Manuscripts

Associations between active travel and diet: cross-sectional evidence on healthy, low-carbon behaviours from UK Biobank

Michaela A. Smith*[1], Jan R. Böhnke[2], Hilary Graham[1], Piran C. L. White[3], Stephanie L. Prady[1]

1. Department of Health Sciences, University of York, York, YO10 5DD, UK
2. Dundee Centre for Health And Related Research, School of Nursing and Health Sciences (SNHS), University of Dundee, Dundee DD1 4HJ, UK
3. Department of Environment and Geography, University of York, York, YO10 5NG, UK

Word Count: 3,833

Abstract: 288

Number of tables: 5

Number of figures: 0

* Corresponding author:

Michaela Smith

Department of Health Sciences

Seebohm Rowntree Building

University of York

Heslington, York

YO10 5DD, UK

michaela.smith@alumni.york.ac.uk

For peer review only - http://bmjopen.bmj.com/site/about/guidelines.xhtml

ABSTRACT

Objectives To examine whether there are associations between active travel and markers of a healthy, low-carbon diet (increased consumption of fruit and vegetables, reduced consumption of red and processed meat).

Design Cross-sectional analysis of a cohort study.

Settings Population cohort of over 500,000 people recruited from 22 centres across the United Kingdom. Participants aged between 40 and 69 years were recruited between 2006 and 2010.

Participants 412,299 adults with complete data on travel mode use, consumption of fruit and vegetables and red and processed meat, and socio-demographic covariates were included in the analysis.

Exposure measures Mutually exclusive mode or mode combinations of travel for non-work and commuting journeys.

Outcome measures Consumption of fruit and vegetables (FV) measured as portions per day and red and processed meat (RPM) measured as frequency per week.

Results Engaging in all types of active travel was positively associated with higher FV consumption and negatively associated with more frequent RPM consumption. Cycling exclusively or in combination with walking was most strongly associated with increased dietary consumption of FV and reduced consumption of RPM for both non-work and commuting journeys. Overall, the strongest associations were between non-work cycling and FV consumption (males: adjusted odds ratio=2.18, 95% confidence interval=2.06, 2.30; females: 2.50, 2.31, 2.71) and non-work cycling and RPM consumption (males: 0.57, 0.54, 0.60; females: 0.54, 0.50, 0.59). Associations were generally similar for both commuting and non-work travel, and were robust to adjustment with socio-demographic and behavioural factors.

Conclusions There are strong associations between engaging in active travel, particularly cycling, and healthy, low-carbon (HLC) dietary consumption, suggesting that these HLC behaviours are related. Further research is needed to better understand the drivers and dynamics between these behaviours within individuals, and whether they share common underlying causes.

For peer review only - http://bmjopen.bmj.com/site/about/guidelines.xhtml

Article summary

Strengths and limitations of this study

- This study uses the UK Biobank to examine associations between active travel and diet in order to better understand the patterning of healthy, low-carbon lifestyles.
- UK Biobank is a large cohort with very rich data, which enabled assessment of relationships using several measures of travel and dietary behaviour and adjustment for a wide range of socio-demographic, environmental, and behavioural covariates.
- The analysis used in this study is cross-sectional and therefore we cannot infer causality between these behaviours.
- This study is further limited by the use of self-reported measures of active travel and dietary consumption.

For peer review only

INTRODUCTION

Increasing concerns about global climate change combined with rising rates of chronic disease have led to greater policy attention on behaviours and lifestyles that are beneficial for both human health and the natural environment [1-3]. From this perspective, two priority behaviours that have been identified are engaging in healthy, low-carbon travel (e.g. walking and cycling for transport) and consuming healthy, low-carbon diets (e.g. reduced consumption of meat, increased consumption of fruit and vegetables) [4-8]. Modelling studies have shown that a population shift toward these behaviours would lead to a range of health and environmental co-benefits: increased life expectancies, decreased rates of Type 2 diabetes, cardiovascular diseases and cancer, as well as large reductions in transport and food-related greenhouse gas emissions [3 6 7 9-12]. These shifts are also in line with national health guidelines. In the UK, for example, walking and cycling for transport is widely recommended as a key strategy to increase population physical activity [13 14], and adults are advised to base at least two-thirds of their diet on plant-sourced foods, specifically consuming at least 5 portions of fruit and vegetables (FV) and less than 70 grams (g) of red and processed meat (RPM) per day to prevent chronic disease outcomes [15 16]. These dietary principles are also in alignment with the recently published 'planetary health diet' which argues that huge changes in consumption of fruits, vegetables, and red meat are needed on a global scale if we are to stay within safe planetary boundaries [17].

In the UK, studies that have examined these travel and dietary behaviours at the population level have found that they are strongly patterned by socio-demographic factors [18-22], which suggests that healthy, low-carbon (HLC) behaviours may overlap among certain population groups and/or within specific environments. Nevertheless, it remains unclear whether these behaviours actually co-occur within the same individuals, as there are very few studies that have examined HLC travel and dietary behaviours in relation to each other. For example, evidence from surveys and psychological research has shown that people who are willing to drive less (or drive more efficiently) are also more willing to

For peer review only - http://bmjopen.bmj.com/site/about/guidelines.xhtml

reduce their meat consumption, but these associations have been limited to behavioural intentions rather than actual travel behaviour and food consumption [23-25]. At the same time, there is considerable evidence of positive associations between physical activity and consuming more healthful diets [26-29], but it is not known whether this relationship also extends to forms of physically active *travel* or to diets that are both healthy and low-carbon. Based on this evidence, it has been proposed that strategies to promote active travel could also offer additional population health benefits through indirect dietary outcomes [30], but these relationships are poorly understood and have not yet been tested empirically. Determining whether behaviours co-occur is important because if behaviours are related, engaging in one behaviour may modify the likelihood of engaging in another [31-34]. This means that strategies which target multiple behaviours together may have additional benefits over the sum of individual interventions [35], and therefore have the potential to produce synergistic outcomes.

In light of these gaps, the objective of this study was to explore relationships between HLC behaviours in the travel and dietary domains, by examining associations between engaging in active travel and consumption of two food groups (FV, RPM) that have contrasting implications for human health and carbon emissions. Our choice of measures was based on the behaviours for which there are UK government recommendations and for which there is the greatest evidence of combined public health and environment benefits. As far as we are aware, there has been no prior research that has explicitly examined the relationships between these combinations of diet and travel behaviour.

METHODS

Study design and sample

We used baseline data from UK Biobank (project 14840) to assess cross-sectional relationships between use of different travel modes and dietary consumption. The scientific rationale, study design, and survey methods for UK Biobank (UKB) have been described elsewhere [36]. Briefly, data were collected from 502,616 individuals aged 40–69 years recruited between 2006 and 2010. Participants

For peer review only - http://bmjopen.bmj.com/site/about/guidelines.xhtml

were identified from National Health Service patient registers and invited to attend one of 22 assessment centres located throughout the UK. At each assessment centre, participants completed a touchscreen questionnaire that collected information on socio-demographic characteristics and diet, lifestyle, and environmental factors. UKB received ethics approval from the National Information Governance Board for Health and Social Care and the National Health Service North West Centre for Research Ethics Committee (Ref: 11/NW/0382).

In this study, participants who did not provide any information on travel mode use (n=7,272) or dietary consumption (FV or RPM, n=1,820) were excluded, yielding an initial sample size of 493,524. This number was then further restricted to participants who had complete data on all analytical covariates (n=412,299 for all journeys, n=234,148 for commuting journeys). Sensitivity analyses were conducted with a further subsample that had complete data on weekly physical activity (PA) and total energy intake (95,475 females and 83,213 males).

Measures

Travel mode use

Data on travel behaviour were collected on the touchscreen questionnaire. Participants were asked to report which travel mode(s) they used for non-work journeys (*In the last 4 weeks, which forms of transport have you used most often to get about?*) and for their travel to work (commuting journeys), if they were currently employed and did not always work from home (*What types of transport do you use to get to and from work?*). Both questions had the same response options (car/motor vehicle, public transport, walking, cycling), and allowed participants to select multiple modes for each type of journey.

Using these two questions, we categorised travel behaviour in several ways. First, to create an overall measure of active travel for each participant, we combined the responses from the two travel questions into one binary variable which included those who reported any walking or any cycling for

For peer review only - http://bmjopen.bmj.com/site/about/guidelines.xhtml

either non-work or commuting journeys. Similar binary variables were also created for any walking and any cycling across the two types of journeys. Second, to account for all possible combinations of travel, a 15-category travel mode variable was derived for each type of journey (non-work, commuting) in order to organize the modal combinations from those producing the most carbon emissions and requiring the least physical exertion (car use only), to those producing the least emissions and requiring the most physical exertion (cycling only or cycling + walking). This was then collapsed into an eight-category variable for each type of journey: (1) car only, (2) car + public transport only, (3) car + public and active transport, (4) car + active transport only, (5) public transport only, (6) public + active transport, (7) walking only, (8) cycling only or cycling + walking. This approach is similar to that used previously by Flint and Cummins [37].

Dietary consumption

Data on FV and RPM consumption also came from the touchscreen questionnaire. Participants were asked to report their FV consumption via four open-ended questions that asked about the average number of tablespoons of vegetables and pieces of fruit consumed each day. These responses were then recoded into standard '5-a-day' portions [38] that resulted in an overall measure of average portions of FV consumed for each participant. To assess whether each participant's consumption was in line with the recommended guideline, this variable was also recoded into a three-level ordinal measure: <3, 3 to <5, and 5+ portions/day.

Participants were asked five questions about their average weekly intake of different types of meat. To create an overall measure of RPM consumption, we combined the four questions involving RPM (beef, lamb, pork, processed meat) into a composite index, based on the number of times each type of meat was consumed on a weekly basis [39]. For each meat type, the responses were coded as follows: Never = 0, Less than once a week = 0.5, Once a week = 1, 2-4 times a week = 3, 5-6 times a week = 5.5, once or more daily = 7. This index variable ranged from 0 to 28, where 0 indicated that

For peer review only - http://bmjopen.bmj.com/site/about/guidelines.xhtml

participants never consumed any RPM and 28 indicated that participants consumed all four types of RPM on a daily basis. Based on the distribution of the resulting index variable, RPM consumption was then grouped into three categories: (1) non-consumers, and consumers spilt at the median frequency: (2) up to 3 times per week, (3) >3 times per week. This approach was used by Bradbury, et al. [39], who showed that those who consume RPM most frequently (>3 times per week) in the UKB sample also consume the largest quantities per day.

Covariates

Various demographic, socioeconomic, and environmental factors were hypothesised as possible confounders to relationships between travel behaviour and dietary consumption. Demographic covariates were age at baseline, sex, ethnic origin, and household size. Socioeconomic covariates were gross annual household income, number of cars per household, highest educational qualification, and occupational class. We used the National Statistics Socioeconomic Classification for occupation class by converting codes from Standard Occupational Classification (SOC) 2000. Environmental covariates were residential area classification, Townsend deprivation score, and region of UK. Weekly PA (meeting or not meeting PA guideline) and total energy intake (kcal) were used in sensitivity analyses. Those who reported 150 minutes of moderate PA or 75 minutes of vigorous PA per week were considered to meet the current PA guideline [40]. Data on total energy intake came from a 24-h dietary recall questionnaire which was completed at the assessment centre by the last 70,000 participants and up to four times by email in the rest of the cohort [41]. For respondents who completed multiple dietary recall questionnaires, we used the average value.

Covariates were mostly self-reported on the touchscreen questionnaire, with the exception of occupational class (verbal interview), residential area classification (census), Townsend deprivation score (census), region of UK (assessment centre location), and average energy intake (24-h dietary assessment).

For peer review only - http://bmjopen.bmj.com/site/about/guidelines.xhtml

Statistical Analysis

Associations between each measure of travel behaviour and each dietary outcome were examined using multivariate ordinal regression models in Stata/SE 14.0 [42]. We used ordinal logistic regression in order to model the trends in dietary consumption while keeping the 'extremes' as useful categories (e.g. non-consumers of RPM, and those who met or exceeded consumption guidelines). This enabled meaningful interpretation of the relationships with a view to national dietary recommendations and potentially discontinuous changes in the associations between travel and dietary behaviour. Though these relationships could plausibly go in either direction, we modelled them in this way based on previous hypotheses [30] as well as neurocognitive research which suggests that physical activity may be more likely to lead to dietary changes than vice versa [43 44].

In Model 1 we examined the bivariate association between each travel variable and each dietary outcome and in Model 2 we adjusted for socio-demographic and environmental covariates. As a sensitivity analysis we further adjusted for physical activity and energy intake (Model 3) in the subsample with complete data on these factors (for comparison purposes Models 1 and 2 were re-run in this subsample as well). This sensitivity analysis was only conducted for the any active travel variable since this contained all of the other active travel combinations.

In each regression model, we tested the proportional odds assumption using the Stata *oparallel* post-estimation command [45]. Where this assumption was not met ($p<0.05$), we re-ran each model as a generalised ordered logit model (Stata extension *gologit2*) which relaxes the proportional odds assumption for some predictor variables while maintaining it for others [46]. This approach has the advantages of being more parsimonious and interpretable than those estimated by a non-ordinal method and may also give added insights (e.g. discontinuous changes) into the data that would be lost by ignoring the differences and continuing to use the fully ordinal model [47]. We present odds ratios (OR) or adjusted odds ratios (aOR) with 95% confidence intervals (CI) and set a threshold of

For peer review only - http://bmjopen.bmj.com/site/about/guidelines.xhtml

alpha=0.05 for statistical significance. All analyses were stratified by sex due to established gender differences in the patterning of travel behaviour and dietary consumption in the UK population [19 21 48 49].

Patient and Public Involvement

This study was conducted using the UK Biobank resource. Details of patient and public involvement in the UK Biobank are available online (https://www.ukbiobank.ac.uk/public-consultation/). No patients were specifically involved in setting the research question or the outcome measures, nor were they involved in developing plans for recruitment, design or implementation of this study. No patients were asked to advise on interpretation or writing up of results. There are no specific plans to disseminate the results of the research to study participants, but the UK Biobank disseminates key findings from projects on its website (https://www.ukbiobank.ac.uk/participant-events/).

RESULTS

Descriptive characteristics of the sample are presented in Table 1. As well as being older, UK Biobank participants are more socioeconomically advantaged, and more health-conscious in comparison with the UK general population [50]. In this study 54.5% of the sample reported walking or cycling for either type of journey (any active travel), and walking was much more common than cycling (51.6% vs. 9.4%) (Table 2). Car only travel was much higher for commuting journeys (63.1%) than for non-work journeys (39.5%), indicating that people were more likely to use multiple modes and more active modes for non-work travel. For example, 22.0% of the sample mixed car use with active modes, and 14.0% mixed car, active modes and public transport use for non-work journeys. For diet, 58.3% of males and 36.7% of females reported consuming RPM more than three times per week and only 5.3% of the sample reported never consuming any RPM (3.4% among males, 7.0% among females) (Table 3). Nearly 39% reported consuming 5+ portions of FV per day on average (31.4% among males, 43.3% among females).

For peer review only - http://bmjopen.bmj.com/site/about/guidelines.xhtml

Associations between travel modes and FV consumption

Across all models, there were positive associations between all types of HLC travel and FV consumption among both males and females, with very little change even after adjustment for demographic, socioeconomic, and environmental factors (Table 4). Associations were generally much stronger for cycling than for other travel modes. For example, in the fully adjusted models (Model 2), men and women who engaged in any cycling travel were nearly twice as likely to consume higher amounts of FV than those who did not cycle for transport (males: aOR=1.65, 95%CI 1.61, 1.69; females: aOR=1.67, 95%CI 1.62, 1.73). Across the more detailed travel classifications of non-work and commuting journeys, associations were generally weaker or non-significant for travel that did not involve any walking or cycling (e.g. car + public transport, public transport only). Comparing across the two types of journeys, the associations were fairly similar in magnitude, though they were slightly stronger for non-work travel, and particularly for non-work cycling. Based on the confidence intervals, women who engaged in any active travel (aOR=1.43, 95%CI 1.40, 1.45) or any walking travel (aOR=1.38, 95%CI 1.36, 1.41) were more likely to consume higher amounts of FV compared with males (aOR=1.35, 95%CI 1.33, 1.37 and aOR=1.25, 95%CI 1.23, 1.27, respectively). For non-work journeys, the same was also true for women who used car + active travel, walking only, and cycling / cycling + walking, compared with their male counterparts.

Associations between travel modes and RPM consumption

Overall, the associations between HLC travel and RPM consumption were nearly all negative; the only exception was for car + public transport (versus car only travel) among females for non-work journeys (Table 5). Among both males and females, associations were only slightly attenuated with adjustment for demographic, socioeconomic, and environmental factors. As with FV consumption, these associations were strongest for cycling, overall and across both types of journeys. Moreover, there was a clear gradient of effect for non-work travel, such that the more active the travel mode(s),

For peer review only - http://bmjopen.bmj.com/site/about/guidelines.xhtml

the more negative the association with RPM consumption frequency. For example, in the fully adjusted models (Model 2), men and women who cycled for non-work journeys were nearly half as likely to consume RPM more frequently than those who travelled by car (males: aOR=0.57; 95%CI 0.54, 0.60; females: aOR=0.54, 95%CI 0.50, 0.59).

Proportional odds assumption

Due to the very large sample size in UKB, we were able to detect very minor variations in the data, and this meant that all of the models in Tables 4 and 5 had violations of the proportional odds assumption. To assess whether these differences were meaningful for the key variables of interest (travel variables), all of the models were re-run using a generalised ordered logit model (Supplementary Appendix). Here the associations were generally of similar magnitude and in the same direction to the fully ordinal models, but where differences were present, the associations tended to be slightly stronger for the two highest categories versus the lowest category of the outcome variables, for example, 3+ portions of FV versus <3, and RPM consumers versus never consumers. This relatively trivial difference does not alter the directions of the associations (positive and negative) in our main findings.

Sensitivity analyses

In the subset of the sample with full data on energy intake and physical activity (n=95,475 females, n=83,213 males), adjusting for these variables in addition to the other socio-demographic and environmental factors slightly attenuated the associations between any active travel and FV consumption, but the relationship was still independent and highly significant among both males and females (males: aOR=1.28; 95%CI 1.24, 1.31 and females: aOR=1.35, 95%CI 1.32, 1.39) (Supplementary Appendix). Similarly, the associations between any active travel and RPM consumption were also very slightly attenuated, but even less so than for FV consumption (males: aOR= 0.89; 95%CI 0.87, 0.92 and females: aOR=0.90, 95%CI 0.88, 0.92) (Supplementary Appendix).

For peer review only - http://bmjopen.bmj.com/site/about/guidelines.xhtml

DISCUSSION

To our knowledge, this is the first analysis to explicitly examine relationships between engaging in active travel and HLC dietary consumption, thus beginning to clarify the patterning of HLC lifestyles. We have shown that engaging in active travel, and in particular cycling, is associated with increased consumption of FV and with reduced consumption of RPM in the UKB sample. In almost all cases, the associations were robust to adjustment by both socio-demographic and behavioural factors, suggesting that these factors do not explain the observed relationships. Using multiple measures of travel and dietary behaviour, we have assessed these relationships comprehensively across different travel modes, types of journeys, and relevant food groups, and also adjusted for a wide range of important covariates. This level of detail has allowed us to isolate and elucidate where the relationships between these HLC behaviours are strongest and weakest, which is an important contribution to understanding which elements of travel and dietary behaviour may share common underlying factors.

The major strength of this study is the large sample size and flexible measures of travel behaviour in the UKB dataset, both of which enabled the observation of relatively fine-grained differences in the data. Nevertheless, UKB is limited by its lack of representativeness, as it is based on a sample of 'healthy volunteers' [50] and excludes large segments of the population (e.g. those under age 40). The data were also collected between 2006 and 2010, and there have been some population changes in meat consumption since then, though less so among those in the UKB age range [51]. As a result, it is unclear whether these results are generalizable to the UK general population, however similar relationships were also found when this analysis was replicated in a nationally representative UK sample [52 53], which supports the external validity of these associations.

Other limitations include that the measures used were all self-reported and that the analyses are cross-sectional. If participants were more likely to report that they walked, cycled, ate more FV and ate less RPM, then this might partially explain the observed associations between these behaviours.

For peer review only - http://bmjopen.bmj.com/site/about/guidelines.xhtml

The cross-sectional nature of the data means we cannot establish causality between these behaviours in terms of whether active travel precedes higher FV and lower RPM consumption, vice versa, or whether change occurs in tandem, or when in the life course such patterns emerge or change. Future research with longitudinal data will help to confirm the direction of these relationships, as well as improve our understanding of behaviour dynamics over time.

Importantly, the findings of this study confirm much of the wider evidence on links between health and environmental behaviours, and represent some of the strongest evidence to date on this topic. Several studies have reported clustering between increased physical activity and more nutritious diets [26-29 54], but this study is the first to show that comparable associations exist for *physically active travel* and healthy diets, independent of overall physical activity. Our findings also build upon studies of environmental behaviours which have linked reduced car driving with reduced meat consumption, but which have only measured behavioural intentions [24 25]. More indirectly, there are also interesting parallels between this study and the growing body of evidence relating active travel, and particularly active commuting, to positive health outcomes like lower obesity and reduced mortality [19 37 55-58]. Two of these studies, also conducted using UKB but only examining active commuting, have found particularly strong effects for cycling to work and lower obesity [37] and reduced mortality [57], far over and above the effects found for walking. Combining these findings with our results on the dietary patterns of cyclists suggests that positive interactions between cycling travel and HLC diets could be one factor contributing to the enhanced health effects observed among individuals who cycle.

This study has several important implications. Firstly, the results suggest that active travel and HLC diets may be related and share similar determinants within individuals. Theoretical understandings of behavioural co-occurrence suggest that behaviours which cluster together share common causal pathways [31 32], and that the stronger the relationship between two behaviours, the more determinants they are likely to share [59]. In this study, strong relationships were seen most clearly

For peer review only - http://bmjopen.bmj.com/site/about/guidelines.xhtml

between cycling and FV consumption, even after adjusting for socio-demographic characteristics and behavioural factors like overall physical activity and energy intake. This suggests that these behaviours may be driven by common underlying factors, and supports the interpretation of both behaviours being related to health motivations, though there may also be other factors at play.

Identifying whether two behaviours are related is important because strongly associated behaviours may influence each other in different ways [31-34]. In the case of positive relationships, this could mean that related behaviours have the potential to produce synergistic outcomes, if strategies that target multiple HLC behaviours together have greater benefits than the sum of individual interventions [35]. Urgent changes in lifestyles are needed if we are to avoid catastrophic climate change [60 61]. Putting these changes into action requires that we have a complete understanding of people's behaviour patterns, including how different behaviours influence, interact and intersect with one another across the life course. Though relationships between active travel and diet still need to be examined longitudinally, this study suggests that these HLC behaviours may have the potential to positively influence one another, and that promotion of these behaviours could help foster enhanced benefits for both human health and the natural environment.

For peer review only

Contributors

All authors made substantial contributions to the conception and design of the study and interpretation of data. MAS undertook the statistical analysis with input from JRB and SLP. MAS drafted the article and all authors revised it critically for important intellectual content. All authors approved the final version of the manuscript to be published.

Funding

MAS was supported by a PhD studentship from the University of York as part of the Health of Populations and Ecosystems (HOPE) project, funded by the Economic and Social Research Council (grant number ES/L003015/1), awarded to HG and PCLW.

For peer review only - http://bmjopen.bmj.com/site/about/guidelines.xhtml

Acknowledgement

This research has been conducted using the UK Biobank Resource under Application Number 14840.

Competing interests

None declared.

Data sharing statement

Additional data available in Supplemental Appendix. Dataset can be obtained from UK Biobank.

Licence statement

I, the Submitting Author has the right to grant and does grant on behalf of all authors of the Work (as defined in the below author licence), an exclusive licence and/or a non-exclusive licence for contributions from authors who are: i) UK Crown employees; ii) where BMJ has agreed a CC-BY licence shall apply, and/or iii) in accordance with the terms applicable for US Federal Government officers or employees acting as part of their official duties; on a worldwide, perpetual, irrevocable, royalty-free basis to BMJ Publishing Group Ltd ("BMJ") its licensees and where the relevant Journal is co-owned by BMJ to the co-owners of the Journal, to publish the Work in BMJ Open and any other BMJ products and to exploit all rights, as set out in our licence.

The Submitting Author accepts and understands that any supply made under these terms is made by BMJ to the Submitting Author unless you are acting as an employee on behalf of your employer or a postgraduate student of an affiliated institution which is paying any applicable article publishing charge ("APC") for Open Access articles. Where the Submitting Author wishes to make the Work available on an Open Access basis (and intends to pay the relevant APC), the terms of reuse of such Open Access shall be governed by a Creative Commons licence – details of these licences and which Creative Commons licence will apply to this Work are set out in our licence referred to above.

For peer review only - http://bmjopen.bmj.com/site/about/guidelines.xhtml

Table 1 – Descriptive characteristics of sample (n=412,299)

	Males		Females		All	
	n	%	n	%	n	%
Total	195,131	47.3	217,168	52.7	412,299	100.0
Age at baseline (years) [a]						
38-44	20,476	10.5	23,892	11.0	44,368	10.8
45-49	25,246	12.9	31,543	14.5	56,789	13.8
50-54	28,821	14.8	36,394	16.8	65,215	15.8
55-59	34,774	17.8	40,910	18.8	75,684	18.4
60-64	46,955	24.1	50,174	23.1	97,129	23.6
65-73	38,859	19.9	34,255	15.8	73,114	17.7
Ethnic group						
White British	175,294	89.8	193,220	89.0	368,514	89.4
Other White	10,855	5.6	13,903	6.4	24,758	6.0
South Asian	3,835	2.0	2,870	1.3	6,705	1.6
Black	2,403	1.2	3,306	1.5	5,709	1.4
Chinese	450	0.2	720	0.3	1,170	0.3
Mixed	891	0.5	1,448	0.7	2,339	0.6
Other	1,403	0.7	1,701	0.8	3,104	0.8
Highest qualification [b]						
College or University degree	70,136	35.9	74,613	34.4	144,749	35.1
A levels or equivalent	20,898	10.7	27,183	12.5	48,081	11.7
GCSEs or equivalent	36,862	18.9	51,055	23.5	87,917	21.3
CSEs or equivalent	10,560	5.4	11,730	5.4	22,290	5.4
NVQ or HND or HNC or equivalent	17,732	9.1	9,607	4.4	27,339	6.6
Other professional qualifications	8,560	4.4	12,375	5.7	20,935	5.1
No qualifications	30,383	15.6	30,605	14.1	60,988	14.8
Occupational Class [c]						
Higher managerial & professional	48,981	25.1	25,058	11.5	74,039	18.0
Lower managerial & professional	34,686	17.8	54,458	25.1	89,144	21.6
Intermediate occupations	14,933	7.7	36,723	16.9	51,656	12.5
Small employers & own accounts	9,345	4.8	4,958	2.3	14,303	3.5
Lower supervisory & technical	10,702	5.5	1,019	0.5	11,721	2.8
Semi-routine occupations	10,986	5.6	20,181	9.3	31,167	7.6
Routine occupations	10,162	5.2	5,365	2.5	15,527	3.8
Not classified	55,336	28.4	69,406	32.0	124,742	30.3
Household income (before tax)						
Less than £18,000	39,184	20.1	52,863	24.3	92,047	22.3
£18,000 to 30,999	47,701	24.5	57,347	26.4	105,048	25.5
£31,000 to 51,999	52,674	27.0	55,578	25.6	108,252	26.3
£52,000 to 100,000	43,674	22.4	40,867	18.8	84,541	20.5
Greater than £100,000	11,898	6.1	10,513	4.8	22,411	5.4

For peer review only

For peer review only - http://bmjopen.bmj.com/site/about/guidelines.xhtml

Table 1 (continued)

	Males		Females		All	
	n	%	n	%	n	%
Household size						
1	33,345	17.1	45,334	20.9	78,679	19.1
2	90,130	46.2	98,297	45.3	188,427	45.7
3	30,803	15.8	33,989	15.7	64,792	15.7
4	29,408	15.1	28,809	13.3	58,217	14.1
5+	11,445	5.9	10,739	4.9	22,184	5.4
Number of cars per household						
0	14,877	7.6	19,055	8.8	33,932	8.2
1	77,536	39.7	95,160	43.8	172,696	41.9
2	79,161	40.6	79,599	36.7	158,760	38.5
3	17,829	9.1	17,994	8.3	35,823	8.7
4+	5,728	2.9	5,360	2.5	11,088	2.7
Region [d]						
London	25,333	13.0	30,273	13.9	55,606	13.5
South East England	17,007	8.7	19,402	8.9	36,409	8.8
South West England	16,764	8.6	19,613	9.0	36,377	8.8
East Midlands	13,120	6.7	14,559	6.7	27,679	6.7
West Midlands	18,383	9.4	18,020	8.3	36,403	8.8
Yorkshire and the Humber	29,615	15.2	32,479	15.0	62,094	15.1
North East England	23,110	11.8	25,606	11.8	48,716	11.8
North West England	29,599	15.2	31,717	14.6	61,316	14.9
Wales	8,265	4.2	9,048	4.2	17,313	4.2
Scotland	13,935	7.1	16,451	7.6	30,386	7.4
Urban residence	167,547	85.9	186,617	85.9	354,164	85.9
Townsend score (mean, sd) [e]	-1.37	3.1	-1.37	3.0	-1.37	3.0

[a] Continuous variable in models

[b] A levels: academic advanced-levels, post compulsory education; GCSEs: academic General Certificate of Secondary Education, formerly Ordinary Levels, taken at age 15–16 years and the end of compulsory education ; CSEs: vocational Certificate of Secondary Education, formerly taken at age 15–16 years; NVQ, HND, HNC: National Vocational Qualifications, Higher National Diploma, Higher National Certificate, all intermediate semi-vocational qualifications

[c] Based on National Statistics Socio-economic Classification (NS-SEC), where Not classified = those who were retired, unemployed, looking after home/family, unable to work because of sickness/disability, doing unpaid/voluntary work, or full-time students

[d] Grouped based on assessment centre: London = St Barts, Croydon, Hounslow; South East England = Oxford, Reading; South West England = Bristol; East Midlands = Nottingham; West Midlands = Birmingham; Yorkshire and the Humber = Leeds, Sheffield; North East England = Middlesbrough, Newcastle; North West England = Liverpool, Manchester, Bury; Wales = Cardiff, Swansea, Wrexham; Scotland = Glasgow, Edinburgh.

[e] Lower score = less deprived (min: -6.3; max: 11.0)

For peer review only - http://bmjopen.bmj.com/site/about/guidelines.xhtml

Table 2 – Descriptive overview of travel mode use (n=412,299)

	Males (n=195,131)		Females (n=217,168)		All (n=412,299)	
	n	%	n	%	n	%
Any active travel [a]	105,287	54.0	119,244	54.9	224,531	54.5
Any walking travel	96,976	49.7	115,573	53.2	212,549	51.6
Any cycling travel	24,806	12.7	13,877	6.4	38,683	9.4
Non-work journeys [b]						
Car only	79,582	40.9	82,980	38.3	162,562	39.5
Car + PT	6,058	3.1	8,822	4.1	14,880	3.6
Car + mixed (PT and AT)	25,683	13.2	32,024	14.8	57,707	14.0
Car + AT	44,488	22.8	46,117	21.3	90,605	22.0
PT only	9,957	5.1	13,277	6.1	23,234	5.6
PT + AT	11,793	6.1	16,020	7.4	27,813	6.8
Walking only	12,553	6.5	14,939	6.9	27,492	6.7
Cycling / cycling + walking	4,660	2.4	2,648	1.2	7,308	1.8
Missing	357		341		698	
Commuting journeys [c]						
Car only	74,043	65.5	73,736	60.9	147,779	63.1
Car + PT	6,735	6.0	7,519	6.2	14,254	6.1
Car + mixed (PT and AT)	3,649	3.2	3,578	3.0	7,227	3.1
Car + AT	7,573	6.7	8,727	7.2	16,300	7.0
PT only	8,383	7.4	12,042	10.0	20,425	8.7
PT + AT	4,861	4.3	5,081	4.2	9,942	4.3
Walking only	3,878	3.4	8,183	6.8	12,061	5.2
Cycling / cycling + walking	3,964	3.5	2,196	1.8	6,160	2.6
Missing / not applicable	82,045		96,106		178,151	

PT: public transport; AT: active travel

[a] Includes walking or cycling for non-work or commuting travel

[b] n=411,601 for non-work travel (0.2% missing), % are calculated from non-missing

[c] n=234,148 for commuting travel (43.2% missing/not applicable), % are calculated from non-missing

For peer review only - http://bmjopen.bmj.com/site/about/guidelines.xhtml

Table 3 – Descriptive overview of dietary consumption and physical activity (n=412,299)

	Males (n=195,131)		Females (n=217,168)		All (n=412,299)	
	n	%	n	%	n	%
FV consumption (portions/day)						
< 3	66,672	34.2	45,669	21.0	112,341	27.3
3 to < 5	67,263	34.5	77,583	35.7	144,846	35.1
5+	61,196	31.4	93,917	43.3	155,112	37.6
RPM consumption (frequency/week)						
Never	6,615	3.4	15,250	7.0	21,865	5.3
≤ 3 times	74,766	38.3	122,148	56.3	196,914	47.8
> 3 times	113,750	58.3	79,770	36.7	193,520	46.9
Meets physical activity guideline [a]						
Yes	101,323	54.1	103,804	50.0	205,127	52.0
No	86,112	45.9	103,996	50.0	189,108	48.0
Missing	7,696		10,368		18,064	
Total energy intake, kcal/day (mean, sd) [b]	2,299	685	1,971	575	2,123	649

[a] n=394,235 for physical activity guideline (4.4% missing), % are calculated from non-missing

[b] Based on n=98,853 females, n=85,392 males

For peer review only

Table 4 – Ordinal logistic models between HLC travel and FV consumption, stratified by gender (n=412,299)

TRAVEL VARIABLES	Males (n=195,131)		Females (n=217,168)	
	Model 1 [a]	Model 2 [b]	Model 1 [a]	Model 2 [b]
	OR (95% CI)			
Any active travel (ref: None)	1.37*** (1.34 - 1.39)	1.35*** (1.33 - 1.37)	1.42*** (1.40 - 1.44)	1.43*** (1.40 - 1.45)
Any walking (ref: None)	1.28*** (1.26 - 1.31)	1.25*** (1.23 - 1.27)	1.38*** (1.36 - 1.40)	1.38*** (1.36 - 1.41)
Any cycling (ref: None)	1.57*** (1.54 - 1.61)	1.65*** (1.61 - 1.69)	1.58*** (1.53 - 1.63)	1.67*** (1.62 - 1.73)
Non-work travel [c] (ref: Car only)				
Car + public transport	1.06* (1.01 - 1.11)	1.00 (0.95 - 1.05)	1.05* (1.01 - 1.09)	0.98 (0.94 - 1.02)
Car + mixed (public and active)	1.49*** (1.46 - 1.53)	1.37*** (1.33 - 1.40)	1.57*** (1.53 - 1.61)	1.41*** (1.38 - 1.45)
Car + active travel	1.27*** (1.24 - 1.29)	1.26*** (1.24 - 1.29)	1.37*** (1.34 - 1.40)	1.39*** (1.36 - 1.42)
Public transport only	1.03 (0.99 - 1.07)	1.13*** (1.08 - 1.18)	1.03 (0.99 - 1.06)	1.11*** (1.06 - 1.15)
Public transport + active travel	1.31*** (1.27 - 1.36)	1.43*** (1.37 - 1.49)	1.45*** (1.40 - 1.50)	1.52*** (1.47 - 1.58)
Walking only	1.34*** (1.29 - 1.38)	1.39*** (1.34 - 1.44)	1.47*** (1.42 - 1.52)	1.57*** (1.51 - 1.62)
Cycling / cycling + walking	2.06*** (1.95 - 2.17)	2.18*** (2.06 - 2.30)	2.34*** (2.17 - 2.52)	2.50*** (2.31 - 2.71)
Commuting travel [d] (ref: Car only)				
Car + public transport	1.03 (0.98 - 1.08)	0.97 (0.92 - 1.01)	1.02 (0.97 - 1.06)	0.99 (0.95 - 1.03)
Car + mixed (public and active)	1.44*** (1.36 - 1.53)	1.37*** (1.29 - 1.46)	1.45*** (1.36 - 1.54)	1.41*** (1.32 - 1.50)
Car + active travel	1.41*** (1.35 - 1.47)	1.47*** (1.41 - 1.54)	1.23*** (1.18 - 1.28)	1.30*** (1.24 - 1.35)
Public transport only	1.08*** (1.03 - 1.12)	1.03 (0.98 - 1.08)	0.91*** (0.88 - 0.95)	0.95* (0.91 - 0.99)
Public transport + active travel	1.41*** (1.33 - 1.48)	1.37*** (1.29 - 1.45)	1.26*** (1.19 - 1.32)	1.28*** (1.20 - 1.35)
Walking only	1.20*** (1.13 - 1.28)	1.24*** (1.16 - 1.32)	1.07*** (1.03 - 1.12)	1.20*** (1.14 - 1.25)
Cycling / cycling + walking	1.78*** (1.68 - 1.89)	1.82*** (1.71 - 1.93)	1.93*** (1.77 - 2.09)	2.00*** (1.84 - 2.18)

*** $p<0.001$, ** $p<0.01$, * $p<0.05$

[a] Model 1: unadjusted

[b] Model 2: adjusted for age, ethnic group, education, occupational class, household income, household size, number of cars, assessment centre location, population density, Townsend score (Full models in supplementary appendix)

[c] n=194,775 males, n=216,828 females

[d] n=113,087 males, n=121,063 females

For peer review only - http://bmjopen.bmj.com/site/about/guidelines.xhtml

Table 5 – Ordinal logistic models between HLC travel and RPM consumption, stratified by gender (n=412,299)

TRAVEL VARIABLES	Males (n=195,131)		Females (n=217,168)	
	Model 1 [a]	Model 2 [b]	Model 1 [a]	Model 2 [b]
	OR (95% CI)			
Any active travel (ref: None)	0.87*** (0.85 - 0.88)	0.89*** (0.87 - 0.91)	0.85*** (0.84 - 0.87)	0.88*** (0.87 - 0.90)
Any walking (ref: None)	0.91*** (0.89 - 0.93)	0.94*** (0.92 - 0.95)	0.88*** (0.86 - 0.89)	0.91*** (0.89 - 0.92)
Any cycling (ref: None)	0.75*** (0.73 - 0.77)	0.76*** (0.74 - 0.78)	0.67*** (0.65 - 0.69)	0.72*** (0.69 - 0.74)
Non-work travel [c] (ref: Car only)				
Car + public transport	0.99 (0.94 - 1.04)	1.01 (0.95 - 1.06)	1.12*** (1.07 - 1.17)	1.09*** (1.04 - 1.14)
Car + mixed (public and active)	0.92*** (0.89 - 0.94)	0.96* (0.94 - 0.99)	0.93*** (0.91 - 0.96)	0.95*** (0.93 - 0.98)
Car + active travel	0.95*** (0.93 - 0.98)	0.96*** (0.94 - 0.98)	0.94*** (0.92 - 0.96)	0.94*** (0.92 - 0.97)
Public transport only	0.91*** (0.87 - 0.95)	0.89*** (0.85 - 0.94)	0.87*** (0.84 - 0.90)	0.88*** (0.84 - 0.91)
Public transport + active travel	0.78*** (0.75 - 0.81)	0.77*** (0.74 - 0.81)	0.71*** (0.69 - 0.74)	0.76*** (0.73 - 0.79)
Walking only	0.76*** (0.73 - 0.78)	0.75*** (0.72 - 0.78)	0.70*** (0.68 - 0.72)	0.71*** (0.69 - 0.74)
Cycling / cycling + walking	0.56*** (0.53 - 0.59)	0.57*** (0.54 - 0.60)	0.50*** (0.46 - 0.54)	0.54*** (0.50 - 0.59)
Commuting travel [d] (ref: Car only)				
Car + public transport	0.94** (0.89 - 0.98)	1.00 (0.95 - 1.06)	1.00 (0.96 - 1.05)	1.04 (0.99 - 1.09)
Car + mixed (public and active)	0.82*** (0.76 - 0.87)	0.89** (0.84 - 0.96)	0.83*** (0.78 - 0.89)	0.93* (0.86 - 0.99)
Car + active travel	0.82*** (0.78 - 0.86)	0.83*** (0.79 - 0.87)	0.92*** (0.88 - 0.96)	0.89*** (0.85 - 0.93)
Public transport only	0.86*** (0.82 - 0.90)	0.95 (0.91 - 1.01)	0.89*** (0.85 - 0.92)	0.97 (0.93 - 1.01)
Public transport + active travel	0.70*** (0.66 - 0.74)	0.79*** (0.74 - 0.84)	0.71*** (0.67 - 0.75)	0.84*** (0.79 - 0.89)
Walking only	0.76*** (0.71 - 0.81)	0.80*** (0.75 - 0.86)	0.89*** (0.85 - 0.93)	0.86*** (0.82 - 0.91)
Cycling / cycling + walking	0.58*** (0.54 - 0.62)	0.60*** (0.56 - 0.64)	0.51*** (0.46 - 0.55)	0.55*** (0.50 - 0.60)

*** $p<0.001$, ** $p<0.01$, * $p<0.05$

[a] Model 1: unadjusted

[b] Model 2: adjusted for age, ethnic group, education, occupational class, household income, household size, number of cars, assessment centre location, population density, Townsend score (Full models in supplementary appendix)

[c] n=194,775 males, n=216,828 females

[d] n=113,087 males, n=121,063 females

For peer review only - http://bmjopen.bmj.com/site/about/guidelines.xhtml

References

1. Watts N, Adger WN, Agnolucci P, et al. Health and climate change: policy responses to protect public health. The Lancet 2015;**386** (10006):1861 - 914 doi: 10.1016/s0140-6736(15)60854-6[published Online First: Epub Date]|.
2. Whitmee S, Haines A, Beyrer C, et al. Safeguarding human health in the Anthropocene epoch: report of The Rockefeller Foundation–Lancet Commission on planetary health. The Lancet 2015;**386**:1973–2028 doi: 10.1016/s0140-6736(15)60901-1[published Online First: Epub Date]|.
3. Haines A, McMichael AJ, Smith KR, et al. Public health benefits of strategies to reduce greenhouse-gas emissions: overview and implications for policy makers. The Lancet 2009;**374**(9707):2104-14
4. Woodcock J, Edwards P, Tonne C, et al. Public health benefits of strategies to reduce greenhouse-gas emissions: urban land transport. The Lancet 2009;**374**(9705):1930-43
5. Capon AG, Synnott ES, Holliday S. Urbanism, climate change and health: systems approaches to governance. New South Wales public health bulletin 2009;**20**(1-2):24-8
6. Friel S, Dangour AD, Garnett T, et al. Public health benefits of strategies to reduce greenhouse-gas emissions: food and agriculture. The Lancet 2009;**374**(9706):2016-25 doi: 10.1016/s0140-6736(09)61753-0[published Online First: Epub Date]|.
7. Aston LM, Smith JN, Powles JW. Impact of a reduced red and processed meat dietary pattern on disease risks and greenhouse gas emissions in the UK: a modelling study. BMJ Open 2012;**2**(5) doi: 10.1136/bmjopen-2012-001072[published Online First: Epub Date]|.
8. Lindsay G, Macmillan A, Woodward A. Moving urban trips from cars to bicycles: impact on health and emissions. Aust N Z J Public Health 2011;**35**(1):54-60 doi: 10.1111/j.1753-6405.2010.00621.x[published Online First: Epub Date]|.
9. Jarrett J, Woodcock J, Griffiths UK, et al. Effect of increasing active travel in urban England and Wales on costs to the National Health Service. The Lancet 2012;**379**(9832):2198-205 doi: 10.1016/s0140-6736(12)60766-1[published Online First: Epub Date]|.
10. Green R, Milner J, Dangour AD, et al. The potential to reduce greenhouse gas emissions in the UK through healthy and realistic dietary change. Climatic Change 2015 doi: 10.1007/s10584-015-1329-y[published Online First: Epub Date]|.
11. Milner J, Green R, Dangour AD, et al. Health effects of adopting low greenhouse gas emission diets in the UK. BMJ Open 2015;**5**(4):e007364 doi: 10.1136/bmjopen-2014-007364[published Online First: Epub Date]|.
12. Tainio M, Monsivais P, Jones NRV, et al. Mortality, greenhouse gas emissions and consumer cost impacts of combined diet and physical activity scenarios: a health impact assessment study. BMJ Open 2017;**7**(2):e014199 doi: 10.1136/bmjopen-2016-014199[published Online First: Epub Date]|.
13. DfT/DH. Active Travel Strategy. London: Department for Transport/Department of Health, 2010.
14. NICE. Walking and cycling: local measures to promote walking and cycling as forms of travel or recreation: National Institute for Health and Clinical Excellence, 2012.
15. NHS. Meat in your diet. Secondary Meat in your diet 2018. https://http://www.nhs.uk/live-well/eat-well/meat-nutrition/.
16. NHS. The Eatwell Guide. Secondary The Eatwell Guide 2019. https://http://www.nhs.uk/live-well/eat-well/the-eatwell-guide/.
17. Willett W, Rockström J, Loken B, et al. Food in the Anthropocene: the EAT–Lancet Commission on healthy diets from sustainable food systems. The Lancet 2019;**393**(10170):447-92
18. Hutchinson J, White PL, Graham H. Differences in the social patterning of active travel between urban and rural populations: findings from a large UK household survey. International journal of public health 2014;**59**(6):993-98 doi: 10.1007/s00038-014-0578-2[published Online First: Epub Date]|.

For peer review only - http://bmjopen.bmj.com/site/about/guidelines.xhtml

19. Laverty AA, Mindell JS, Webb EA, et al. Active travel to work and cardiovascular risk factors in the United Kingdom. Am J Prev Med 2013;**45**(3):282-8 doi: 10.1016/j.amepre.2013.04.012[published Online First: Epub Date]|.
20. Maguire ER, Monsivais P. Socio-economic dietary inequalities in UK adults: an updated picture of key food groups and nutrients from national surveillance data. The British journal of nutrition 2014:1-9 doi: 10.1017/S0007114514002621[published Online First: Epub Date]|.
21. Aston LM, Smith JN, Powles JW. Meat intake in Britain in relation to other dietary components and to demographic and risk factor variables: analyses based on the National Diet and Nutrition Survey of 2000/2001. Journal of Human Nutrition and Dietetics 2013;**26**(1):96-106 doi: 10.1111/j.1365-277X.2012.01278.x[published Online First: Epub Date]|.
22. Leahy E, Lyons S, Tol RSJ. Determinants of vegetarianism and partial vegetarianism in the United Kingdom. Dublin: Economic and Social Research Institute (ESRI) working paper, 2010.
23. Van der Werff E, Steg L, Keizer K. I Am What I Am, by Looking Past the Present: The Influence of Biospheric Values and Past Behavior on Environmental Self-Identity. Environ Behav 2013;**46**(5):626-57 doi: 10.1177/0013916512475209[published Online First: Epub Date]|.
24. de Boer J, de Witt A, Aiking H. Help the climate, change your diet: A cross-sectional study on how to involve consumers in a transition to a low-carbon society. Appetite 2016;**98**:19-27 doi: 10.1016/j.appet.2015.12.001[published Online First: Epub Date]|.
25. Lee L, Simpson I. Are we eating less meat? A British Social Attitudes report. London: NatCen, 2016.
26. Noble N, Paul C, Turon H, et al. Which modifiable health risk behaviours are related? A systematic review of the clustering of Smoking, Nutrition, Alcohol and Physical activity ('SNAP') health risk factors. Prev Med 2015;**81**:16-41 doi: 10.1016/j.ypmed.2015.07.003[published Online First: Epub Date]|.
27. Poortinga W. The prevalence and clustering of four major lifestyle risk factors in an English adult population. Prev Med 2007;**44**(2):124-8 doi: 10.1016/j.ypmed.2006.10.006[published Online First: Epub Date]|.
28. Tormo MJ, Navarro C, Chirlaque M-D, et al. Physical sports activity during leisure time and dietary intake of foods and nutrients in a large Spanish cohort. Int J Sport Nutr Exerc Metab 2003;**13**:47-64
29. Parsons TJ, Power C, Manor O. Longitudinal physical activity and diet patterns in the 1958 British Birth Cohort. Med Sci Sports Exerc 2006;**38**(3):547-54 doi: 10.1249/01.mss.0000188446.65651.67[published Online First: Epub Date]|.
30. de Nazelle A, Nieuwenhuijsen MJ, Anto JM, et al. Improving health through policies that promote active travel: a review of evidence to support integrated health impact assessment. Environ Int 2011;**37**(4):766-77 doi: 10.1016/j.envint.2011.02.003[published Online First: Epub Date]|.
31. McAloney K, Graham H, Law C, et al. A scoping review of statistical approaches to the analysis of multiple health-related behaviours. Prev Med 2013;**56**(6):365-71 doi: 10.1016/j.ypmed.2013.03.002[published Online First: Epub Date]|.
32. Spring B, Moller AC, Coons MJ. Multiple health behaviours: overview and implications. J Public Health (Oxf) 2012;**34 Suppl 1**:i3-10 doi: 10.1093/pubmed/fdr111[published Online First: Epub Date]|.
33. Truelove HB, Carrico AR, Weber EU, et al. Positive and negative spillover of pro-environmental behavior: An integrative review and theoretical framework. Global Environmental Change 2014;**29**:127-38 doi: 10.1016/j.gloenvcha.2014.09.004[published Online First: Epub Date]|.
34. Dolan P, Galizzi MM. Like ripples on a pond: Behavioral spillovers and their implications for research and policy. J Econ Psychol 2015;**47**:1-16 doi: 10.1016/j.joep.2014.12.003[published Online First: Epub Date]|.
35. Spring B, Schneider K, McFadden HG, et al. Multiple behavior changes in diet and activity: a randomized controlled trial using mobile technology. Arch Intern Med 2012;**172**(10):789-96 doi: 10.1001/archinternmed.2012.1044[published Online First: Epub Date]|.

For peer review only

For peer review only - http://bmjopen.bmj.com/site/about/guidelines.xhtml

36. Sudlow C, Gallacher J, Allen N, et al. UK biobank: an open access resource for identifying the causes of a wide range of complex diseases of middle and old age. PLoS Med 2015;**12**(3):e1001779 doi: 10.1371/journal.pmed.1001779[published Online First: Epub Date]|.
37. Flint E, Cummins S. Active commuting and obesity in mid-life: cross-sectional, observational evidence from UK Biobank. The Lancet Diabetes & Endocrinology 2016;**4**(5):420-35 doi: 10.1016/s2213-8587(16)00053-x[published Online First: Epub Date]|.
38. NHS. 5 A Day portion sizes. Secondary 5 A Day portion sizes 2018. https://http://www.nhs.uk/live-well/eat-well/5-a-day-portion-sizes/.
39. Bradbury KE, Tong TYN, Key TJ. Dietary Intake of High-Protein Foods and Other Major Foods in Meat-Eaters, Poultry-Eaters, Fish-Eaters, Vegetarians, and Vegans in UK Biobank. Nutrients 2017;**9**(12):1317
40. NHS. Physical activity guidelines for adults. Secondary Physical activity guidelines for adults 2018. https://http://www.nhs.uk/live-well/exercise//.
41. Galante J, Adamska L, Young A, et al. The acceptability of repeat Internet-based hybrid diet assessment of previous 24-h dietary intake: administration of the Oxford WebQ in UK Biobank. The British journal of nutrition 2016;**115**(4):681-6 doi: 10.1017/s0007114515004821[published Online First: Epub Date]|.
42. Stata Statistical Software: Release 14 [program]. College Station, TX: StataCorp LP, 2015.
43. Joseph RJ, Alonso-Alonso M, Bond DS, et al. The neurocognitive connection between physical activity and eating behaviour. Obes Rev 2011;**12**(10):800-12 doi: 10.1111/j.1467-789X.2011.00893.x[published Online First: Epub Date]|.
44. Loprinzi PD. Physical activity is the best buy in medicine, but perhaps for less obvious reasons. Prev Med 2015;**75**:23-24 doi: 10.1016/j.ypmed.2015.01.033[published Online First: Epub Date]|.
45. oparallel: Stata module providing post-estimation command for testing the parallel regression assumption [program], 2013.
46. Williams R. Generalized ordered logit/partial proportional odds models for ordinal dependent variables. Stata Journal 2006;**6**(1):58
47. Williams R. Understanding and interpreting generalized ordered logit models. The Journal of Mathematical Sociology 2016;**40**(1):7-20
48. Bates B, Lennox A, Prentice A, et al. National Diet and Nutrition Survey: Results from Years 1–4 (combined) of the Rolling Programme (2008/2009–2011/12). Public Health England, and Food Standards Agency: London 2014
49. DfT. National Travel Survey: England 2016. London: Department for Transport, 2017.
50. Fry A, Littlejohns TJ, Sudlow C, et al. Comparison of Sociodemographic and Health-Related Characteristics of UK Biobank Participants with the General Population. Am J Epidemiol 2017 doi: 10.1093/aje/kwx246[published Online First: Epub Date]|.
51. Juliano L. The Grocer: Plant based food, Research on behalf of The Grocer – April 2018: Harris Interactive, 2018.
52. Smith MA, Böhnke JR, Graham H, et al. OP24 Associations between active travel and diet: An exploration of pro-health, low carbon behaviours in the National Diet and Nutrition Survey: BMJ Publishing Group Ltd, 2016.
53. Smith M. Prevalence, patterning, and predictors of health-and climate-relevant lifestyles in the UK: A cross-sectional study of travel and dietary behaviour in two national datasets. University of York, 2018.
54. Gillman MW, Pinto BM, Tennstedt S, et al. Relationships of physical activity with dietary behaviors among adults. Prev Med 2001;**32**(3):295-301 doi: 10.1006/pmed.2000.0812[published Online First: Epub Date]|.
55. Martin A, Panter J, Suhrcke M, et al. Impact of changes in mode of travel to work on changes in body mass index: evidence from the British Household Panel Survey. J Epidemiol Community Health 2015;**69**(8):753-61 doi: 10.1136/jech-2014-205211[published Online First: Epub Date]|.

For peer review only - http://bmjopen.bmj.com/site/about/guidelines.xhtml

56. Flint E, Cummins S, Sacker A. Associations between active commuting, body fat, and body mass index: population based, cross sectional study in the United Kingdom. BMJ 2014;**349** doi: 10.1136/bmj.g4887[published Online First: Epub Date]|.
57. Celis-Morales CA, Lyall DM, Welsh P, et al. Association between active commuting and incident cardiovascular disease, cancer, and mortality: prospective cohort study. BMJ 2017;**357**:j1456 doi: 10.1136/bmj.j1456[published Online First: Epub Date]|.
58. Panter J, Mytton O, Sharp S, et al. Using alternatives to the car and risk of all-cause, cardiovascular and cancer mortality. Heart 2018;**104**(21):1749-55 doi: 10.1136/heartjnl-2017-312699[published Online First: Epub Date]|.
59. Flay B, Petraitis J. The theory of triadic influence. Adv Med Sociol 1994;**4**:19-44
60. IPCC. *Global Warming of 1.5° C: An IPCC Special Report on the Impacts of Global Warming of 1.5° C Above Pre-industrial Levels and Related Global Greenhouse Gas Emission Pathways, in the Context of Strengthening the Global Response to the Threat of Climate Change, Sustainable Development, and Efforts to Eradicate Poverty*: Intergovernmental Panel on Climate Change, 2018.
61. Akenji L, Lettenmeier M, Koide R, et al. 1.5-Degree Lifestyles: Targets and options for reducing lifestyle carbon footprints: Institute for Global Environmental Strategies, Aalto University, and D-mat ltd. (Hayama, Japan) 2019.

For peer review only - http://bmjopen.bmj.com/site/about/guidelines.xhtml

Table 1 – Results of ordinal logistic models between any active travel and fruit and vegetable (FV) consumption among females in UKB (n=217,168)

VARIABLES	Unadjusted	Adjusted
Any active travel (ref: None)	1.42***	1.43***
	(1.40 - 1.44)	(1.40 - 1.45)
Age at baseline		1.04***
		(1.04 - 1.05)
Ethnic group (ref: White British)		
Other white		1.38***
		(1.33 - 1.42)
South Asian		2.19***
		(2.04 - 2.37)
Black		1.63***
		(1.52 - 1.74)
Chinese		1.67***
		(1.45 - 1.93)
Mixed		1.16**
		(1.05 - 1.28)
Other		2.29***
		(2.08 - 2.53)
Highest qualification (ref: Degree level)		
A levels/AS levels or equivalent		0.86***
		(0.83 - 0.88)
O levels/GCSEs or equivalent		0.77***
		(0.75 - 0.79)
CSEs or equivalent		0.72***
		(0.70 - 0.75)
NVQ or HND or HNC or equivalent		0.79***
		(0.76 - 0.82)
Other professional qualifications		0.89***
		(0.86 - 0.92)
No qualifications		0.63***
		(0.62 - 0.65)
Occupation class (ref: Higher man / prof)		
Lower managerial / professional		1.08***
		(1.05 - 1.11)
Intermediate occupations		1.02
		(0.99 - 1.06)
Small employers & own accounts		1.11***
		(1.04 - 1.17)
Lower supervisory & technical		1.16*
		(1.03 - 1.31)
Semi-routine occupations		1.03
		(0.99 - 1.07)
Routine occupations		0.98
		(0.93 - 1.04)
Not classified		1.03
		(1.00 - 1.06)
Household income (ref: £<18 000)		
£18,000 to 30,999		1.10***
		(1.07 - 1.13)
£31,000 to 51,999		1.21***
		(1.17 - 1.24)
£52,000 to 100,000		1.29***
		(1.25 - 1.33)
£Greater than 100,000		1.32***
		(1.26 - 1.38)

For peer review only

Household size (ref: One)		
2		0.95***
		(0.93 - 0.97)
3		0.88***
		(0.86 - 0.91)
4		0.85***
		(0.82 - 0.87)
5+		0.87***
		(0.83 - 0.91)
Region (ref: London)		
North East England		1.07***
		(1.03 - 1.11)
Yorkshire and the Humber		0.99
		(0.96 - 1.02)
West Midlands		1.06***
		(1.03 - 1.10)
East Midlands		1.08***
		(1.04 - 1.12)
South East England		1.12***
		(1.08 - 1.16)
South West England		1.14***
		(1.10 - 1.18)
North West England		0.99
		(0.96 - 1.02)
Wales		1.12***
		(1.07 - 1.17)
Scotland		0.95**
		(0.91 - 0.98)
Townsend deprivation		0.98***
		(0.98 - 0.99)
Urban (ref: Rural)		0.94***
		(0.92 - 0.96)
Cars per household (ref: None)		
One		1.12***
		(1.08 - 1.16)
Two		1.10***
		(1.06 - 1.14)
Three		1.07**
		(1.02 - 1.12)
Four or more		1.09**
		(1.03 - 1.17)
Observations	217,168	217,168

*** p<0.001, ** p<0.01, * p<0.05

For peer review only

Table 2 – Results of ordinal logistic models between any active travel and fruit and vegetable (FV) consumption among males in UKB (n=195,131)

VARIABLES	(1) Unadjusted	(2) Adjusted
Any active travel (ref: None)	1.37***	1.35***
	(1.34 - 1.39)	(1.33 - 1.37)
Age at baseline		1.03***
		(1.03 - 1.03)
Ethnic group (ref: White British)		
Other white		1.32***
		(1.27 - 1.36)
South Asian		2.24***
		(2.10 - 2.38)
Black		1.50***
		(1.39 - 1.63)
Chinese		1.68***
		(1.41 - 2.00)
Mixed		1.11
		(0.98 - 1.26)
Other		2.23***
		(2.02 - 2.47)
Highest qualification (ref: Degree level)		
A levels/AS levels or equivalent		0.79***
		(0.76 - 0.81)
O levels/GCSEs or equivalent		0.75***
		(0.73 - 0.77)
CSEs or equivalent		0.75***
		(0.72 - 0.78)
NVQ or HND or HNC or equivalent		0.83***
		(0.80 - 0.85)
Other professional qualifications		0.82***
		(0.79 - 0.86)
No qualifications		0.75***
		(0.73 - 0.77)
Occupation class (ref: Higher man / prof)		
Lower managerial / professional		1.03*
		(1.00 - 1.05)
Intermediate occupations		1.05**
		(1.01 - 1.09)
Small employers & own accounts		1.04
		(1.00 - 1.09)
Lower supervisory & technical		1.08***
		(1.03 - 1.12)
Semi-routine occupations		1.00
		(0.96 - 1.04)
Routine occupations		1.04
		(0.99 - 1.08)
Not classified		0.99
		(0.96 - 1.02)
Household income (ref: £<18 000)		
£18,000 to 30,999		1.11***
		(1.08 - 1.14)
£31,000 to 51,999		1.18***
		(1.15 - 1.22)
£52,000 to 100,000		1.26***
		(1.22 - 1.31)
£Greater than 100,000		1.33***
		(1.27 - 1.39)

For peer review only

Household size (ref: One)			
	2		1.12***
			(1.09 - 1.15)
	3		1.04*
			(1.01 - 1.08)
	4		1.03
			(1.00 - 1.07)
	5+		1.01
			(0.96 - 1.05)
Region (ref: London)			
	North East England		1.02
			(0.98 - 1.05)
	Yorkshire and the Humber		0.97
			(0.94 - 1.01)
	West Midlands		1.00
			(0.96 - 1.04)
	East Midlands		1.03
			(0.99 - 1.07)
	South East England		1.00
			(0.96 - 1.04)
	South West England		1.02
			(0.98 - 1.06)
	North West England		0.94***
			(0.91 - 0.97)
	Wales		1.05*
			(1.00 - 1.10)
	Scotland		0.83***
			(0.80 - 0.86)
Townsend deprivation			0.99***
			(0.98 - 0.99)
Urban (ref: Rural)			0.96**
			(0.94 - 0.98)
Cars per household (ref: None)			
	One		1.11***
			(1.07 - 1.15)
	Two		1.02
			(0.98 - 1.06)
	Three		0.94**
			(0.89 - 0.98)
	Four or more		0.92**
			(0.86 - 0.98)
Observations		195,131	195,131

*** p<0.001, ** p<0.01, * p<0.05

For peer review only

Table 3 – Results of ordinal logistic models between any active travel and red and processed meat (RPM) consumption among females in UKB (n=217,168)

VARIABLES	Unadjusted	Adjusted
Any active travel (ref: None)	0.85*** (0.84 - 0.87)	0.88*** (0.87 - 0.90)
Age at baseline		1.01*** (1.01 - 1.01)
Ethnic group (ref: White British)		
Other white		0.99 (0.95 - 1.02)
South Asian		0.27*** (0.25 - 0.29)
Black		1.06 (0.99 - 1.14)
Chinese		2.12*** (1.83 - 2.45)
Mixed		0.97 (0.88 - 1.08)
Other		0.91* (0.82 - 1.00)
Highest qualification (ref: Degree level)		
A levels/AS levels or equivalent		1.20*** (1.17 - 1.24)
O levels/GCSEs or equivalent		1.27*** (1.24 - 1.30)
CSEs or equivalent		1.30*** (1.24 - 1.35)
NVQ or HND or HNC or equivalent		1.22*** (1.17 - 1.28)
Other professional qualifications		1.16*** (1.12 - 1.21)
No qualifications		1.35*** (1.31 - 1.39)
Occupation class (ref: Higher man / prof)		
Lower managerial / professional		0.96** (0.93 - 0.99)
Intermediate occupations		1.06** (1.02 - 1.09)
Small employers & own accounts		1.00 (0.94 - 1.07)
Lower supervisory & technical		1.07 (0.94 - 1.21)
Semi-routine occupations		1.08*** (1.04 - 1.13)
Routine occupations		1.20*** (1.13 - 1.27)
Not classified		1.21*** (1.17 - 1.25)
Household income (ref: £<18 000)		
£18,000 to 30,999		1.00 (0.98 - 1.03)
£31,000 to 51,999		0.94*** (0.92 - 0.97)
£52,000 to 100,000		0.93*** (0.90 - 0.96)
£Greater than 100,000		0.93** (0.88 - 0.97)

For peer review only

Household size (ref: One)		
2		1.45***
		(1.41 - 1.48)
3		1.57***
		(1.52 - 1.63)
4		1.79***
		(1.73 - 1.86)
5+		1.92***
		(1.83 - 2.01)
Region (ref: London)		
North East England		0.97
		(0.94 - 1.01)
Yorkshire and the Humber		1.01
		(0.98 - 1.05)
West Midlands		0.97
		(0.93 - 1.00)
East Midlands		1.00
		(0.96 - 1.04)
South East England		1.03
		(0.99 - 1.07)
South West England		0.94***
		(0.90 - 0.97)
North West England		1.15***
		(1.11 - 1.19)
Wales		0.89***
		(0.85 - 0.94)
Scotland		1.20***
		(1.15 - 1.24)
Townsend deprivation		1.00
		(1.00 - 1.00)
Urban (ref: Rural)		0.96**
		(0.94 - 0.98)
Cars per household (ref: None)		
One		1.04*
		(1.01 - 1.08)
Two		1.17***
		(1.12 - 1.21)
Three		1.24***
		(1.18 - 1.30)
Four or more		1.29***
		(1.20 - 1.38)
Observations	217,168	217,168

*** p<0.001, ** p<0.01, * p<0.05

For peer review only

Table 4 – Results of ordinal logistic models between any active travel and red and processed meat (RPM) consumption among males in UKB (n=195,131)

VARIABLES		Unadjusted	Adjusted
Any active travel (ref: None)		0.87*** (0.85 - 0.88)	0.89*** (0.87 - 0.91)
Age at baseline			1.00 (1.00 - 1.00)
Ethnic group (ref: White British)			
	Other white		1.01 (0.97 - 1.05)
	South Asian		0.26*** (0.25 - 0.28)
	Black		0.82*** (0.76 - 0.90)
	Chinese		1.33** (1.09 - 1.61)
	Mixed		1.08 (0.94 - 1.23)
	Other		0.74*** (0.66 - 0.82)
Highest qualification (ref: Degree level)			
	A levels/AS levels or equivalent		1.20*** (1.16 - 1.23)
	O levels/GCSEs or equivalent		1.22*** (1.19 - 1.26)
	CSEs or equivalent		1.22*** (1.17 - 1.27)
	NVQ or HND or HNC or equivalent		1.20*** (1.16 - 1.24)
	Other professional qualifications		1.09*** (1.04 - 1.14)
	No qualifications		1.16*** (1.13 - 1.20)
Occupation class (ref: Higher man / prof)			
	Lower managerial / professional		0.95*** (0.92 - 0.98)
	Intermediate occupations		1.01 (0.97 - 1.05)
	Small employers & own accounts		1.19*** (1.14 - 1.25)
	Lower supervisory & technical		1.18*** (1.13 - 1.23)
	Semi-routine occupations		1.20*** (1.15 - 1.26)
	Routine occupations		1.28*** (1.22 - 1.34)
	Not classified		1.14*** (1.11 - 1.18)
Household income (ref: £<18 000)			
	£18,000 to 30,999		0.96* (0.94 - 0.99)
	£31,000 to 51,999		0.97 (0.94 - 1.01)
	£52,000 to 100,000		0.91*** (0.88 - 0.95)
	£Greater than 100,000		0.89*** (0.85 - 0.94)

For peer review only

Household size (ref: One)		
2		1.06***
		(1.03 - 1.09)
3		1.17***
		(1.13 - 1.21)
4		1.25***
		(1.20 - 1.29)
5+		1.35***
		(1.29 - 1.42)
Region (ref: London)		
North East England		0.99
		(0.95 - 1.03)
Yorkshire and the Humber		1.02
		(0.98 - 1.06)
West Midlands		1.02
		(0.98 - 1.07)
East Midlands		0.97
		(0.93 - 1.01)
South East England		1.03
		(0.99 - 1.07)
South West England		0.96*
		(0.92 - 1.00)
North West England		1.15***
		(1.11 - 1.19)
Wales		0.91***
		(0.87 - 0.96)
Scotland		1.16***
		(1.11 - 1.21)
Townsend deprivation		1.01***
		(1.01 - 1.01)
Urban (ref: Rural)		0.99
		(0.97 - 1.02)
Cars per household (ref: None)		
One		0.98
		(0.94 - 1.02)
Two		1.08***
		(1.04 - 1.13)
Three		1.23***
		(1.17 - 1.30)
Four or more		1.30***
		(1.21 - 1.39)
Observations	195,131	195,131

*** p<0.001, ** p<0.01, * p<0.05

For peer review only

Figure 1 – Associations between travel and FV consumption (Model 2, Table 4)

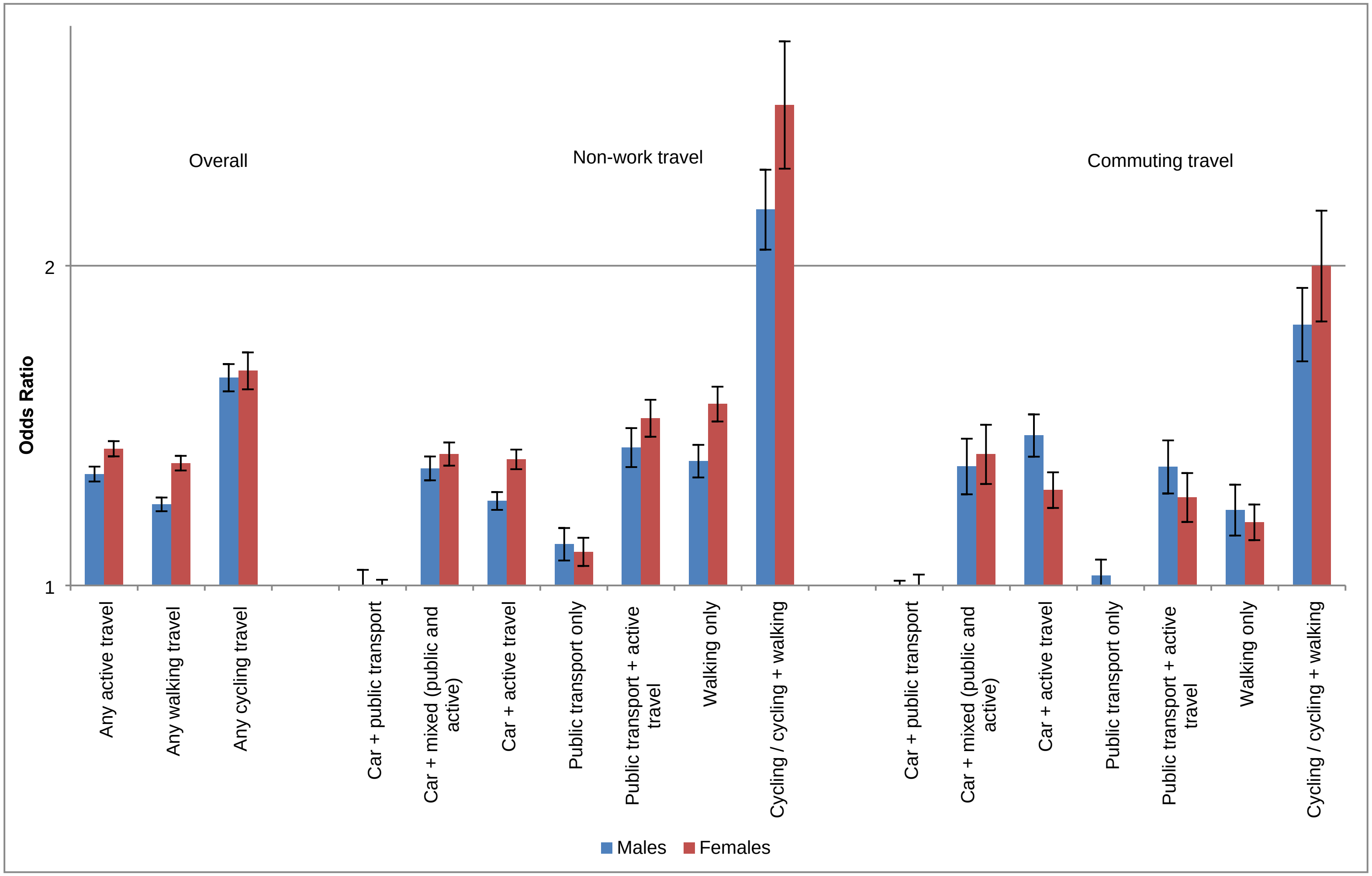

Whiskers = 95% confidence interval

Figure 2 – Associations between travel and RPM consumption (Model 2, Table 5)

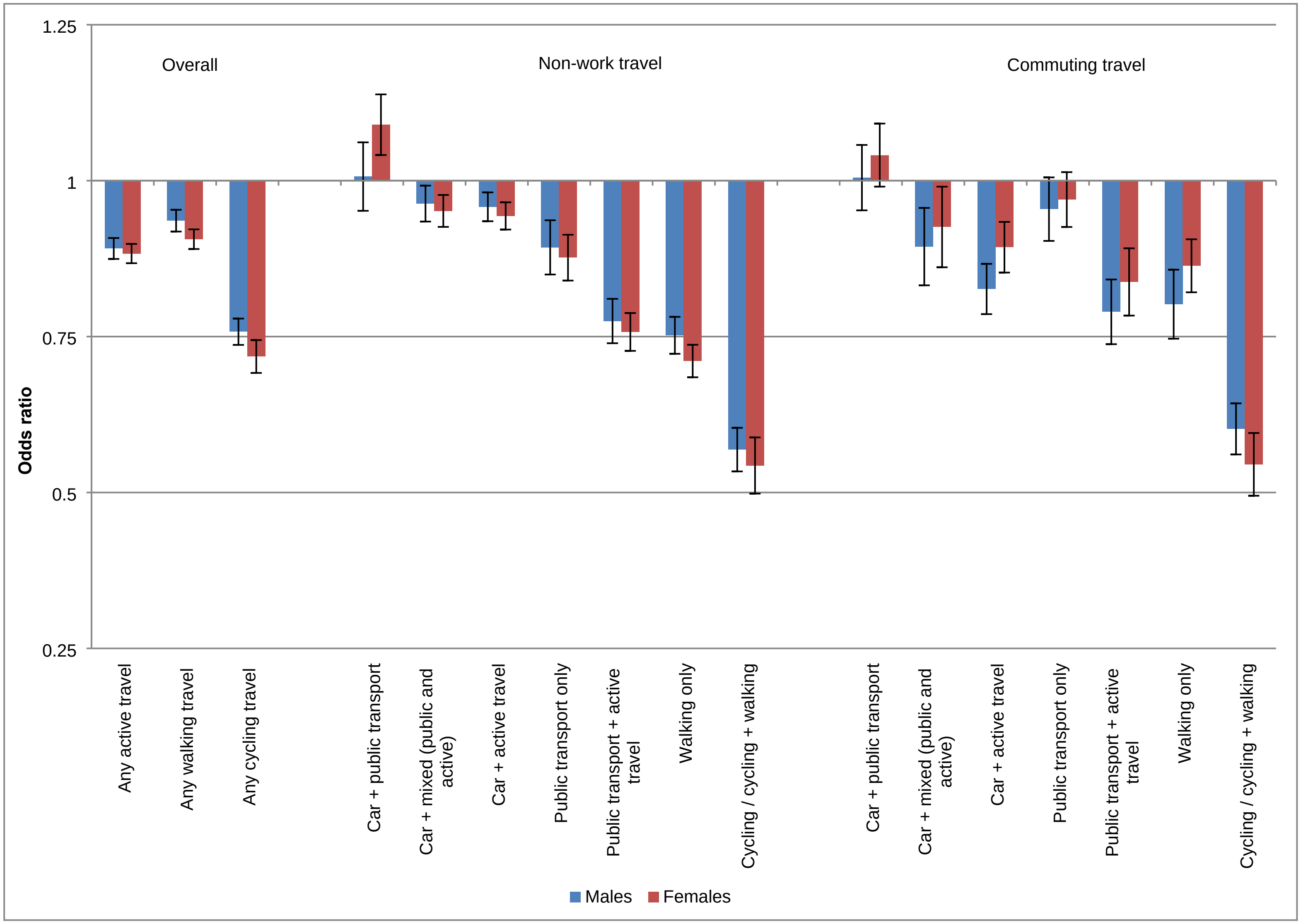

Whiskers = 95% confidence interval

Table 5 – Results of generalized ordered logit models between measures of active travel and FV consumption, stratified by gender in UKB (n=412,299)

TRAVEL VARIABLES	Males (n=195,131)		Females (n=217,168)	
	Model 2 [a]		Model 2 [a]	
	1 v. 2 + 3 [b]	1 + 2 v. 3	1 v. 2 + 3 [b]	1 + 2 v. 3
Any active travel (ref: No)	1.37*** (1.35 - 1.40)	1.32*** (1.30 - 1.35)	1.53*** (1.49 - 1.56)	1.38*** (1.35 - 1.40)
Any walking (ref: No)	1.28*** (1.26 - 1.31)	1.23*** (1.20 - 1.25)	1.47*** (1.44 - 1.51)	1.34*** (1.32 - 1.36)
Any cycling (ref: No)	1.71*** (1.66 - 1.76)	1.61*** (1.57 - 1.66)	1.93*** (1.84 - 2.04)	1.60*** (1.55 - 1.66)
Non-work travel [b] (ref: Car only)				
Car + public transport	1.00 (0.95 - 1.05)	1.00 (0.95 - 1.05)	0.98 (0.94 - 1.02)	0.98 (0.94 - 1.02)
Car + mixed (public and active)	1.42*** (1.38 - 1.47)	1.32*** (1.28 - 1.36)	1.55*** (1.50 - 1.61)	1.36*** (1.32 - 1.39)
Car + active travel	1.29*** (1.26 - 1.32)	1.24*** (1.21 - 1.27)	1.49*** (1.45 - 1.54)	1.35*** (1.32 - 1.38)
Public transport only	1.14*** (1.09 - 1.19)	1.14*** (1.09 - 1.19)	1.11*** (1.07 - 1.16)	1.11*** (1.07 - 1.16)
Public transport + active travel	1.47*** (1.40 - 1.54)	1.39*** (1.33 - 1.46)	1.66*** (1.58 - 1.74)	1.45*** (1.40 - 1.51)
Walking only	1.39*** (1.34 - 1.44)	1.39*** (1.34 - 1.44)	1.62*** (1.55 - 1.69)	1.53*** (1.48 - 1.59)
Cycling / cycling + walking	2.27*** (2.11 - 2.44)	2.10*** (1.97 - 2.23)	2.84*** (2.50 - 3.21)	2.39*** (2.21 - 2.60)
Commuting travel [c] (ref: Car only)				
Car + public transport	0.97 (0.92 - 1.02)	0.97 (0.92 - 1.02)	0.99 (0.95 - 1.03)	0.99 (0.95 - 1.03)
Car + mixed (public and active)	1.38*** (1.29 - 1.47)	1.38*** (1.29 - 1.47)	1.53*** (1.39 - 1.67)	1.37*** (1.28 - 1.47)
Car + active travel	1.47*** (1.41 - 1.54)	1.47*** (1.41 - 1.54)	1.35*** (1.28 - 1.43)	1.28*** (1.22 - 1.33)
Public transport only	1.03 (0.99 - 1.09)	1.03 (0.99 - 1.09)	0.95* (0.91 - 0.99)	0.95* (0.91 - 0.99)
Public transport + active travel	1.37*** (1.29 - 1.46)	1.37*** (1.29 - 1.46)	1.27*** (1.20 - 1.35)	1.27*** (1.20 - 1.35)
Walking only	1.19*** (1.11 - 1.28)	1.29*** (1.20 - 1.38)	1.20*** (1.14 - 1.25)	1.20*** (1.14 - 1.25)
Cycling / cycling + walking	1.82*** (1.71 - 1.94)	1.82*** (1.71 - 1.94)	2.00*** (1.84 - 2.18)	2.00*** (1.84 - 2.18)

*** p<0.001, ** p<0.01, * p<0.05

a) Adjusted for: age, ethnic group, education, occupational class, household income, household size, number of cars, assessment centre location, population density, Townsend score

b) Shading and boxes indicate variables with different relationships across the levels of the outcome variable: 1 = <3 portions FV, 2 = 3-<5 portions FV, 3 = 5+ portions FV

Table 6 – Results of generalized ordered logit models between measures of active travel and RPM consumption, stratified by gender in UKB (n=412,299)

TRAVEL VARIABLES	Males (n=195,131) Model 2 [a] 1 v. 2 + 3 [b]	Males (n=195,131) Model 2 [a] 1 + 2 v. 3	Females (n=217,168) Model 2 [a] 1 v. 2 + 3 [b]	Females (n=217,168) Model 2 [a] 1 + 2 v. 3
Any active travel (ref: No)	0.72*** (0.68 - 0.76)	0.90*** (0.89 - 0.92)	0.79*** (0.76 - 0.81)	0.90*** (0.89 - 0.92)
Any walking (ref: No)	0.86*** (0.81 - 0.90)	0.94*** (0.92 - 0.96)	0.83*** (0.80 - 0.86)	0.92*** (0.91 - 0.94)
Any cycling (ref: No)	0.56*** (0.52 - 0.59)	0.78*** (0.76 - 0.81)	0.63*** (0.60 - 0.67)	0.77*** (0.75 - 0.80)
Non-work travel [b] (ref: Car only)				
Car + public transport	1.01 (0.95 - 1.06)	1.01 (0.95 - 1.06)	1.10*** (1.05 - 1.15)	1.10*** (1.05 - 1.15)
Car + mixed (public and active)	0.78*** (0.72 - 0.84)	0.97 (0.94 - 1.00)	0.87*** (0.83 - 0.92)	0.97* (0.94 - 0.99)
Car + active travel	0.83*** (0.77 - 0.89)	0.96** (0.94 - 0.99)	0.86*** (0.82 - 0.90)	0.96*** (0.93 - 0.98)
Public transport only	0.75*** (0.67 - 0.84)	0.89*** (0.85 - 0.94)	0.87*** (0.83 - 0.90)	0.87*** (0.83 - 0.90)
Public transport + active travel	0.60*** (0.54 - 0.66)	0.79*** (0.75 - 0.82)	0.67*** (0.62 - 0.71)	0.79*** (0.75 - 0.82)
Walking only	0.62*** (0.56 - 0.68)	0.76*** (0.73 - 0.79)	0.64*** (0.60 - 0.68)	0.73*** (0.70 - 0.76)
Cycling / cycling + walking	0.37*** (0.33 - 0.41)	0.61*** (0.58 - 0.65)	0.49*** (0.44 - 0.55)	0.60*** (0.55 - 0.66)
Commuting travel [c] (ref: Car only)				
Car + public transport	1.00 (0.95 - 1.05)	1.00 (0.95 - 1.05)	0.95 (0.87 - 1.04)	1.06* (1.01 - 1.11)
Car + mixed (public and active)	0.58*** (0.51 - 0.67)	0.93* (0.87 - 0.99)	0.77*** (0.69 - 0.85)	0.98 (0.91 - 1.06)
Car + active travel	0.65*** (0.58 - 0.73)	0.84*** (0.80 - 0.88)	0.81*** (0.75 - 0.87)	0.92*** (0.88 - 0.96)
Public transport only	0.82*** (0.73 - 0.92)	0.96 (0.91 - 1.01)	0.96 (0.92 - 1.01)	0.96 (0.92 - 1.01)
Public transport + active travel	0.57*** (0.50 - 0.65)	0.82*** (0.77 - 0.88)	0.73*** (0.66 - 0.80)	0.89*** (0.83 - 0.95)
Walking only	0.65*** (0.56 - 0.75)	0.82*** (0.76 - 0.87)	0.79*** (0.73 - 0.86)	0.88*** (0.84 - 0.93)
Cycling / cycling + walking	0.39*** (0.35 - 0.44)	0.65*** (0.61 - 0.70)	0.46*** (0.41 - 0.52)	0.65*** (0.59 - 0.71)

*** p<0.001, ** p<0.01, * p<0.05

a) Adjusted for: age, ethnic group, education, occupational class, household income, household size, number of cars, assessment centre location, population density, Townsend score

b) Shading and boxes indicate variables with different relationships across the levels of the outcome variable: 1 = 0 g RPM per day; 2 = >0-70 g RPM per day; 3 = >70 g RPM per day

For peer review only - http://bmjopen.bmj.com/site/about/guidelines.xhtml

Table 7 – Sensitivity analysis: results of ordinal logistic models between any active travel and FV consumption among females in UKB (n=95,475)

VARIABLES	Unadjusted	Model 1	Model 2
Any active travel (ref: None)	1.42*** (1.38 - 1.45)	1.42*** (1.38 - 1.45)	1.35*** (1.32 - 1.39)
Age at baseline		1.05*** (1.04 - 1.05)	1.05*** (1.04 - 1.05)
Ethnic group (ref: White British)			
Other white		1.39*** (1.32 - 1.46)	1.38*** (1.31 - 1.45)
South Asian		2.05*** (1.81 - 2.33)	2.09*** (1.84 - 2.37)
Black		1.60*** (1.43 - 1.80)	1.58*** (1.41 - 1.76)
Chinese		1.54*** (1.23 - 1.94)	1.55*** (1.23 - 1.95)
Mixed		1.06 (0.91 - 1.22)	1.05 (0.90 - 1.21)
Other		2.13*** (1.82 - 2.48)	2.16*** (1.85 - 2.53)
Highest qualification (ref: Degree level)			
A levels/AS levels or equivalent		0.87*** (0.84 - 0.90)	0.87*** (0.84 - 0.91)
O levels/GCSEs or equivalent		0.78*** (0.75 - 0.81)	0.79*** (0.76 - 0.81)
CSEs or equivalent		0.71*** (0.66 - 0.75)	0.71*** (0.67 - 0.76)
NVQ or HND or HNC or equivalent		0.82*** (0.77 - 0.88)	0.82*** (0.76 - 0.88)
Other professional qualifications		0.92** (0.87 - 0.97)	0.91** (0.86 - 0.96)
No qualifications		0.68*** (0.64 - 0.71)	0.68*** (0.64 - 0.72)
Occupation class (ref: Higher man / prof)			
Lower managerial / professional		1.07*** (1.03 - 1.11)	1.05** (1.01 - 1.10)
Intermediate occupations		1.01 (0.97 - 1.06)	1.00 (0.96 - 1.05)
Small employers & own accounts		1.12** (1.04 - 1.22)	1.08 (0.99 - 1.17)
Lower supervisory & technical		1.14 (0.96 - 1.37)	1.04 (0.87 - 1.25)
Semi-routine occupations		1.10** (1.04 - 1.16)	1.06 (1.00 - 1.12)
Routine occupations		0.96 (0.86 - 1.07)	0.89* (0.80 - 0.99)
Not classified		1.04 (0.99 - 1.09)	0.99 (0.95 - 1.04)
Household income (ref: £<18 000)			
£18,000 to 30,999		1.11*** (1.06 - 1.15)	1.11*** (1.07 - 1.16)
£31,000 to 51,999		1.23*** (1.18 - 1.28)	1.25*** (1.19 - 1.30)
£52,000 to 100,000		1.31*** (1.25 - 1.37)	1.32*** (1.26 - 1.39)
£Greater than 100,000		1.34*** (1.25 - 1.43)	1.33*** (1.24 - 1.42)

For peer review only

Household size (ref: One)			
2		0.94***	0.93***
		(0.90 - 0.97)	(0.90 - 0.97)
3		0.90***	0.90***
		(0.86 - 0.94)	(0.86 - 0.94)
4		0.84***	0.84***
		(0.80 - 0.89)	(0.80 - 0.88)
5+		0.84***	0.82***
		(0.79 - 0.90)	(0.77 - 0.88)
Region (ref: London)			
North East England		1.11***	1.14***
		(1.05 - 1.16)	(1.08 - 1.20)
Yorkshire and the Humber		1.06**	1.07**
		(1.01 - 1.11)	(1.02 - 1.12)
West Midlands		1.05	1.07**
		(1.00 - 1.11)	(1.02 - 1.13)
East Midlands		1.15***	1.17***
		(1.09 - 1.22)	(1.11 - 1.25)
South East England		1.18***	1.21***
		(1.12 - 1.24)	(1.15 - 1.27)
South West England		1.19***	1.21***
		(1.14 - 1.26)	(1.16 - 1.28)
North West England		1.07**	1.09***
		(1.02 - 1.12)	(1.04 - 1.14)
Wales		1.16***	1.20***
		(1.08 - 1.26)	(1.11 - 1.29)
Scotland		1.09**	1.12***
		(1.03 - 1.16)	(1.05 - 1.19)
Townsend deprivation		0.99***	0.99***
		(0.99 - 0.99)	(0.99 - 1.00)
Urban (ref: Rural)		0.93***	0.95**
		(0.90 - 0.97)	(0.91 - 0.98)
Cars per household (ref: None)			
One		1.00	1.01
		(0.95 - 1.06)	(0.95 - 1.06)
Two		0.96	0.95
		(0.90 - 1.02)	(0.90 - 1.01)
Three		0.92*	0.91*
		(0.86 - 0.99)	(0.85 - 0.98)
Four or more		0.96	0.95
		(0.87 - 1.06)	(0.86 - 1.05)
Meets physical activity guideline (ref: No)			1.65***
			(1.61 - 1.69)
Total energy intake (kcal)			1.00***
			(1.00 - 1.00)
Observations	95,475	95,475	95,475

*** p<0.001, ** p<0.01, * p<0.05

For peer review only

Table 8 – Sensitivity analysis: results of ordinal logistic models between any active travel and FV consumption among males in UKB (n=83,213)

VARIABLES	Unadjusted	Model 1	Model 2
Any active travel (ref: None)	1.38*** (1.34 - 1.41)	1.35*** (1.32 - 1.39)	1.28*** (1.24 - 1.31)
Age at baseline		1.03*** (1.03 - 1.03)	1.03*** (1.03 - 1.03)
Ethnic group (ref: White British)			
Other white		1.28*** (1.21 - 1.35)	1.28*** (1.21 - 1.35)
South Asian		2.02*** (1.81 - 2.25)	2.13*** (1.91 - 2.38)
Black		1.42*** (1.25 - 1.62)	1.44*** (1.26 - 1.64)
Chinese		1.59** (1.20 - 2.12)	1.65*** (1.24 - 2.20)
Mixed		1.14 (0.94 - 1.37)	1.12 (0.93 - 1.35)
Other		2.04*** (1.72 - 2.43)	2.10*** (1.76 - 2.49)
Highest qualification (ref: Degree level)			
A levels/AS levels or equivalent		0.78*** (0.75 - 0.82)	0.79*** (0.76 - 0.82)
O levels/GCSEs or equivalent		0.76*** (0.73 - 0.79)	0.76*** (0.73 - 0.79)
CSEs or equivalent		0.74*** (0.69 - 0.79)	0.73*** (0.68 - 0.78)
NVQ or HND or HNC or equivalent		0.84*** (0.80 - 0.89)	0.83*** (0.79 - 0.87)
Other professional qualifications		0.87*** (0.82 - 0.93)	0.86*** (0.80 - 0.92)
No qualifications		0.79*** (0.75 - 0.84)	0.78*** (0.74 - 0.83)
Occupation class (ref: Higher man / prof)			
Lower managerial / professional		1.03 (1.00 - 1.07)	1.02 (0.99 - 1.06)
Intermediate occupations		1.06* (1.01 - 1.12)	1.05 (1.00 - 1.11)
Small employers & own accounts		1.08 (1.00 - 1.16)	0.99 (0.92 - 1.07)
Lower supervisory & technical		1.13*** (1.06 - 1.21)	1.02 (0.95 - 1.09)
Semi-routine occupations		0.99 (0.92 - 1.06)	0.92* (0.86 - 0.98)
Routine occupations		1.04 (0.97 - 1.13)	0.94 (0.87 - 1.01)
Not classified		0.96* (0.92 - 1.00)	0.92*** (0.88 - 0.95)
Household income (ref: £<18 000)			
£18,000 to 30,999		1.09*** (1.04 - 1.14)	1.08** (1.03 - 1.13)
£31,000 to 51,999		1.20*** (1.15 - 1.26)	1.20*** (1.14 - 1.25)
£52,000 to 100,000		1.28*** (1.21 - 1.35)	1.29*** (1.23 - 1.37)
£Greater than 100,000		1.35*** (1.26 - 1.45)	1.35*** (1.27 - 1.45)

For peer review only

Household size (ref: One)			
2		1.13***<br>(1.08 - 1.18)	1.12***<br>(1.07 - 1.17)
3		1.02<br>(0.97 - 1.07)	1.02<br>(0.97 - 1.07)
4		1.02<br>(0.96 - 1.07)	1.01<br>(0.96 - 1.06)
5+		0.99<br>(0.93 - 1.06)	0.97<br>(0.91 - 1.04)
Region (ref: London)			
North East England		1.05<br>(1.00 - 1.11)	1.06*<br>(1.01 - 1.12)
Yorkshire and the Humber		1.03<br>(0.99 - 1.08)	1.03<br>(0.99 - 1.08)
West Midlands		0.98<br>(0.93 - 1.03)	0.98<br>(0.93 - 1.04)
East Midlands		1.11**<br>(1.04 - 1.18)	1.11**<br>(1.04 - 1.18)
South East England		1.04<br>(0.98 - 1.10)	1.06*<br>(1.01 - 1.12)
South West England		1.07*<br>(1.02 - 1.13)	1.08**<br>(1.02 - 1.13)
North West England		1.00<br>(0.95 - 1.05)	1.00<br>(0.96 - 1.05)
Wales		1.12**<br>(1.03 - 1.21)	1.14**<br>(1.05 - 1.23)
Scotland		0.91**<br>(0.85 - 0.97)	0.93*<br>(0.87 - 0.99)
Townsend deprivation		1.00<br>(0.99 - 1.00)	1.00<br>(0.99 - 1.00)
Urban (ref: Rural)		0.95**<br>(0.92 - 0.99)	0.96*<br>(0.93 - 1.00)
Cars per household (ref: None)			
One		1.01<br>(0.95 - 1.07)	1.00<br>(0.94 - 1.06)
Two		0.92**<br>(0.86 - 0.98)	0.89***<br>(0.83 - 0.95)
Three		0.84***<br>(0.78 - 0.91)	0.81***<br>(0.75 - 0.87)
Four or more		0.81***<br>(0.73 - 0.90)	0.78***<br>(0.71 - 0.87)
Meets physical activity guideline (ref: No)			1.69***<br>(1.64 - 1.73)
Total energy intake (kcal)			1.00***<br>(1.00 - 1.00)
Observations	83,213	83,213	83,213

*** p<0.001, ** p<0.01, * p<0.05

For peer review only

Table 9 – Sensitivity analysis: results of ordinal logistic models between any active travel and RPM consumption among females in UKB (n=95,475)

VARIABLES	Unadjusted	Model 1	Model 2
Any active travel (ref: None)	0.86*** (0.83 - 0.88)	0.89*** (0.87 - 0.91)	0.90*** (0.88 - 0.92)
Age at baseline		1.01*** (1.01 - 1.01)	1.01*** (1.01 - 1.01)
Ethnic group (ref: White British)			
Other white		1.02 (0.97 - 1.07)	1.02 (0.97 - 1.07)
South Asian		0.32*** (0.28 - 0.36)	0.33*** (0.29 - 0.37)
Black		1.09 (0.98 - 1.23)	1.09 (0.97 - 1.22)
Chinese		1.83*** (1.46 - 2.30)	1.87*** (1.49 - 2.35)
Mixed		1.00 (0.86 - 1.17)	1.01 (0.86 - 1.18)
Other		0.75*** (0.65 - 0.88)	0.75*** (0.65 - 0.87)
Highest qualification (ref: Degree level)			
A levels/AS levels or equivalent		1.21*** (1.16 - 1.26)	1.22*** (1.18 - 1.27)
O levels/GCSEs or equivalent		1.28*** (1.23 - 1.32)	1.30*** (1.26 - 1.35)
CSEs or equivalent		1.29*** (1.20 - 1.38)	1.33*** (1.24 - 1.42)
NVQ or HND or HNC or equivalent		1.20*** (1.11 - 1.29)	1.24*** (1.15 - 1.33)
Other professional qualifications		1.15*** (1.08 - 1.21)	1.16*** (1.10 - 1.23)
No qualifications		1.29*** (1.22 - 1.37)	1.34*** (1.27 - 1.42)
Occupation class (ref: Higher man / prof)			
Lower managerial / professional		0.96 (0.92 - 1.00)	0.96* (0.92 - 1.00)
Intermediate occupations		1.06* (1.01 - 1.11)	1.06* (1.01 - 1.11)
Small employers & own accounts		1.00 (0.92 - 1.09)	1.01 (0.93 - 1.10)
Lower supervisory & technical		1.04 (0.86 - 1.26)	1.07 (0.89 - 1.29)
Semi-routine occupations		1.07* (1.01 - 1.14)	1.07* (1.01 - 1.14)
Routine occupations		1.19** (1.06 - 1.33)	1.19** (1.07 - 1.34)
Not classified		1.16*** (1.11 - 1.21)	1.16*** (1.11 - 1.22)
Household income (ref: £<18 000)			
£18,000 to 30,999		1.04 (1.00 - 1.08)	1.05* (1.01 - 1.09)
£31,000 to 51,999		0.99 (0.95 - 1.03)	1.00 (0.96 - 1.05)
£52,000 to 100,000		0.99 (0.94 - 1.04)	1.01 (0.96 - 1.06)
£Greater than 100,000		0.99 (0.93 - 1.06)	1.04 (0.97 - 1.11)

For peer review only

Household size (ref: One)			
2		1.41***<br>(1.36 - 1.47)	1.41***<br>(1.35 - 1.46)
3		1.52***<br>(1.45 - 1.60)	1.50***<br>(1.43 - 1.58)
4		1.77***<br>(1.68 - 1.87)	1.74***<br>(1.65 - 1.83)
5+		1.91***<br>(1.78 - 2.05)	1.86***<br>(1.74 - 2.00)
Region (ref: London)			
North East England		0.95*<br>(0.90 - 1.00)	0.94*<br>(0.89 - 0.99)
Yorkshire and the Humber		0.99<br>(0.95 - 1.04)	0.98<br>(0.94 - 1.03)
West Midlands		0.93**<br>(0.88 - 0.98)	0.92**<br>(0.87 - 0.97)
East Midlands		0.97<br>(0.91 - 1.03)	0.95<br>(0.90 - 1.02)
South East England		1.03<br>(0.98 - 1.09)	1.02<br>(0.96 - 1.08)
South West England		0.91***<br>(0.87 - 0.96)	0.90***<br>(0.86 - 0.95)
North West England		1.07**<br>(1.02 - 1.12)	1.06*<br>(1.01 - 1.11)
Wales		0.85***<br>(0.78 - 0.92)	0.84***<br>(0.77 - 0.91)
Scotland		1.08*<br>(1.02 - 1.15)	1.06<br>(1.00 - 1.13)
Townsend deprivation		0.99***<br>(0.98 - 0.99)	0.99***<br>(0.98 - 0.99)
Urban (ref: Rural)		0.97<br>(0.93 - 1.01)	0.96*<br>(0.93 - 1.00)
Cars per household (ref: None)			
One		1.19***<br>(1.12 - 1.26)	1.19***<br>(1.13 - 1.26)
Two		1.34***<br>(1.26 - 1.43)	1.36***<br>(1.27 - 1.45)
Three		1.42***<br>(1.32 - 1.54)	1.45***<br>(1.35 - 1.57)
Four or more		1.36***<br>(1.23 - 1.51)	1.40***<br>(1.26 - 1.55)
Meets physical activity guideline (ref: No)			0.81***<br>(0.79 - 0.83)
Total energy intake (kcal)			1.00***<br>(1.00 - 1.00)
Observations	95,475	95,475	95,475

*** p<0.001, ** p<0.01, * p<0.05

For peer review only

Table 10 – Sensitivity analysis: results of ordinal logistic models between any active travel and RPM consumption among males in UKB (n=83,213)

VARIABLES	Unadjusted	Model 1	Model 2
Any active travel (ref: None)	0.86***	0.89***	0.89***
	(0.84 - 0.88)	(0.86 - 0.91)	(0.87 - 0.92)
Age at baseline		1.00*	1.00***
		(1.00 - 1.00)	(1.00 - 1.01)
Ethnic group (ref: White British)			
Other white		0.99	0.99
		(0.93 - 1.05)	(0.94 - 1.05)
South Asian		0.27***	0.28***
		(0.24 - 0.30)	(0.25 - 0.31)
Black		0.95	0.99
		(0.82 - 1.09)	(0.86 - 1.15)
Chinese		1.36	1.42*
		(1.00 - 1.86)	(1.03 - 1.94)
Mixed		1.17	1.19
		(0.96 - 1.44)	(0.97 - 1.46)
Other		0.59***	0.60***
		(0.49 - 0.70)	(0.50 - 0.71)
Highest qualification (ref: Degree level)			
A levels/AS levels or equivalent		1.16***	1.17***
		(1.11 - 1.22)	(1.11 - 1.22)
O levels/GCSEs or equivalent		1.20***	1.22***
		(1.15 - 1.25)	(1.17 - 1.27)
CSEs or equivalent		1.19***	1.22***
		(1.11 - 1.28)	(1.14 - 1.32)
NVQ or HND or HNC or equivalent		1.20***	1.23***
		(1.13 - 1.27)	(1.16 - 1.30)
Other professional qualifications		1.01	1.03
		(0.94 - 1.09)	(0.96 - 1.11)
No qualifications		1.10**	1.13***
		(1.04 - 1.17)	(1.07 - 1.20)
Occupation class (ref: Higher man / prof)			
Lower managerial / professional		0.97	0.96*
		(0.93 - 1.01)	(0.92 - 1.00)
Intermediate occupations		1.02	1.02
		(0.97 - 1.08)	(0.97 - 1.08)
Small employers & own accounts		1.07	1.06
		(0.99 - 1.16)	(0.98 - 1.15)
Lower supervisory & technical		1.13**	1.14***
		(1.05 - 1.22)	(1.05 - 1.22)
Semi-routine occupations		1.19***	1.18***
		(1.10 - 1.28)	(1.10 - 1.27)
Routine occupations		1.22***	1.23***
		(1.12 - 1.33)	(1.13 - 1.33)
Not classified		1.13***	1.13***
		(1.08 - 1.18)	(1.08 - 1.18)
Household income (ref: £<18 000)			
£18,000 to 30,999		0.91***	0.92***
		(0.87 - 0.96)	(0.87 - 0.96)
£31,000 to 51,999		0.96	0.97
		(0.91 - 1.01)	(0.92 - 1.02)
£52,000 to 100,000		0.90***	0.92**
		(0.85 - 0.96)	(0.87 - 0.98)
£Greater than 100,000		0.85***	0.88***
		(0.79 - 0.91)	(0.82 - 0.95)

For peer review only

Household size (ref: One)			
2		1.04	1.05*
		(1.00 - 1.09)	(1.00 - 1.09)
3		1.16***	1.15***
		(1.10 - 1.22)	(1.09 - 1.22)
4		1.27***	1.26***
		(1.21 - 1.35)	(1.19 - 1.33)
5+		1.31***	1.28***
		(1.22 - 1.41)	(1.19 - 1.38)
Region (ref: London)			
North East England		0.94*	0.94*
		(0.89 - 1.00)	(0.89 - 1.00)
Yorkshire and the Humber		0.94**	0.94*
		(0.89 - 0.98)	(0.90 - 0.99)
West Midlands		0.91**	0.91**
		(0.86 - 0.97)	(0.86 - 0.97)
East Midlands		0.86***	0.86***
		(0.80 - 0.92)	(0.80 - 0.92)
South East England		1.00	1.00
		(0.94 - 1.06)	(0.94 - 1.06)
South West England		0.91**	0.91**
		(0.86 - 0.97)	(0.86 - 0.96)
North West England		1.06*	1.07*
		(1.01 - 1.12)	(1.01 - 1.12)
Wales		0.80***	0.80***
		(0.73 - 0.87)	(0.73 - 0.87)
Scotland		1.05	1.04
		(0.98 - 1.12)	(0.97 - 1.12)
Townsend deprivation		1.00	1.00
		(0.99 - 1.00)	(0.99 - 1.00)
Urban (ref: Rural)		0.97	0.97
		(0.94 - 1.01)	(0.93 - 1.01)
Cars per household (ref: None)			
One		1.11**	1.11**
		(1.04 - 1.18)	(1.04 - 1.19)
Two		1.27***	1.28***
		(1.18 - 1.36)	(1.19 - 1.37)
Three		1.44***	1.46***
		(1.33 - 1.57)	(1.34 - 1.59)
Four or more		1.40***	1.43***
		(1.26 - 1.57)	(1.28 - 1.60)
Meets physical activity guideline (ref: No)			0.84***
			(0.82 - 0.87)
Total energy intake (kcal)			1.00***
			(1.00 - 1.00)
Observations	83,213	83,213	83,213

*** p<0.001, ** p<0.01, * p<0.05

For peer review only

STROBE 2007 (v4) checklist of items to be included in reports of observational studies in epidemiology*
Checklist for cohort, case-control, and cross-sectional studies (combined)

Section/Topic	Item #	Recommendation	Reported on page #
**Title and abstract**	1	(*a*) Indicate the study's design with a commonly used term in the title or the abstract	1, 2
		(*b*) Provide in the abstract an informative and balanced summary of what was done and what was found	2
**Introduction**			
Background/rationale	2	Explain the scientific background and rationale for the investigation being reported	4, 5
Objectives	3	State specific objectives, including any pre-specified hypotheses	5
**Methods**			
Study design	4	Present key elements of study design early in the paper	5, 6
Setting	5	Describe the setting, locations, and relevant dates, including periods of recruitment, exposure, follow-up, and data collection	5, 6
Participants	6	(*a*) *Cohort study*—Give the eligibility criteria, and the sources and methods of selection of participants. Describe methods of follow-up<br>*Case-control study*—Give the eligibility criteria, and the sources and methods of case ascertainment and control selection. Give the rationale for the choice of cases and controls<br>*Cross-sectional study*—Give the eligibility criteria, and the sources and methods of selection of participants	5, 6
		(*b*) *Cohort study*—For matched studies, give matching criteria and number of exposed and unexposed<br>*Case-control study*—For matched studies, give matching criteria and the number of controls per case	
Variables	7	Clearly define all outcomes, exposures, predictors, potential confounders, and effect modifiers. Give diagnostic criteria, if applicable	6, 7, 8
Data sources/ measurement	8*	For each variable of interest, give sources of data and details of methods of assessment (measurement). Describe comparability of assessment methods if there is more than one group	6, 7, 8
Bias	9	Describe any efforts to address potential sources of bias	9
Study size	10	Explain how the study size was arrived at	6
Quantitative variables	11	Explain how quantitative variables were handled in the analyses. If applicable, describe which groupings were chosen and why	6, 7, 8
Statistical methods	12	(*a*) Describe all statistical methods, including those used to control for confounding	9
		(*b*) Describe any methods used to examine subgroups and interactions	9, 10
		(*c*) Explain how missing data were addressed	6
		(*d*) *Cohort study*—If applicable, explain how loss to follow-up was addressed<br>*Case-control study*—If applicable, explain how matching of cases and controls was addressed	

For peer review only

		Cross-sectional study—If applicable, describe analytical methods taking account of sampling strategy	
		(*e*) Describe any sensitivity analyses	8
**Results**			
Participants	13*	(a) Report numbers of individuals at each stage of study—eg numbers potentially eligible, examined for eligibility, confirmed eligible, included in the study, completing follow-up, and analysed	6
		(b) Give reasons for non-participation at each stage	NA
		(c) Consider use of a flow diagram	
Descriptive data	14*	(a) Give characteristics of study participants (eg demographic, clinical, social) and information on exposures and potential confounders	10, 17, 18, 19, 20
		(b) Indicate number of participants with missing data for each variable of interest	6, 19, 20
		(c) *Cohort study*—Summarise follow-up time (eg, average and total amount)	
Outcome data	15*	*Cohort study*—Report numbers of outcome events or summary measures over time	
		Case-control study—Report numbers in each exposure category, or summary measures of exposure	
		Cross-sectional study—Report numbers of outcome events or summary measures	10
Main results	16	(*a*) Give unadjusted estimates and, if applicable, confounder-adjusted estimates and their precision (eg, 95% confidence interval). Make clear which confounders were adjusted for and why they were included	11, 12, 21, 22
		(*b*) Report category boundaries when continuous variables were categorized	7, 8, 20
		(*c*) If relevant, consider translating estimates of relative risk into absolute risk for a meaningful time period	
Other analyses	17	Report other analyses done—eg analyses of subgroups and interactions, and sensitivity analyses	9
**Discussion**			
Key results	18	Summarise key results with reference to study objectives	13
Limitations	19	Discuss limitations of the study, taking into account sources of potential bias or imprecision. Discuss both direction and magnitude of any potential bias	13, 14
Interpretation	20	Give a cautious overall interpretation of results considering objectives, limitations, multiplicity of analyses, results from similar studies, and other relevant evidence	14, 15
Generalisability	21	Discuss the generalisability (external validity) of the study results	13
**Other information**			
Funding	22	Give the source of funding and the role of the funders for the present study and, if applicable, for the original study on which the present article is based	15

*Give information separately for cases and controls in case-control studies and, if applicable, for exposed and unexposed groups in cohort and cross-sectional studies.

Note: An Explanation and Elaboration article discusses each checklist item and gives methodological background and published examples of transparent reporting. The STROBE checklist is best used in conjunction with this article (freely available on the Web sites of PLoS Medicine at http://www.plosmedicine.org/, Annals of Internal Medicine at http://www.annals.org/, and Epidemiology at http://www.epidem.com/). Information on the STROBE Initiative is available at www.strobe-statement.org.

For peer review only

Associations between active travel and diet: cross-sectional evidence on healthy, low-carbon behaviours from UK Biobank

Journal:	*BMJ Open*
Manuscript ID	bmjopen-2019-030741.R1
Article Type:	Research
Date Submitted by the Author:	23-Jul-2019
Complete List of Authors:	Smith, Michaela; University of York<br>Boehnke, Jan Rasmus; University of Dundee, School of Nursing and Health Sciences<br>Graham, Hilary; University of York<br>White, Piran; University of York<br>Prady, Stephanie; University of York
**Primary Subject Heading**:	Public health
Secondary Subject Heading:	Health policy
Keywords:	PUBLIC HEALTH, PREVENTIVE MEDICINE, EPIDEMIOLOGY, ACTIVE TRAVEL, DIET

Associations between active travel and diet: cross-sectional evidence on healthy, low-carbon behaviours from UK Biobank

Michaela A. Smith*[1], Jan R. Boehnke[2], Hilary Graham[1], Piran C. L. White[3], Stephanie L. Prady[1]

1. Department of Health Sciences, University of York, York, YO10 5DD, UK
2. School of Nursing and Health Sciences (SNHS), University of Dundee, Dundee DD1 4HJ, UK
3. Department of Environment and Geography, University of York, York, YO10 5NG, UK

Word Count: 4,221

Abstract: 293

Number of tables: 5

Number of figures: 0

* Corresponding author:

Michaela Smith

Department of Health Sciences

Seebohm Rowntree Building

University of York

Heslington, York

YO10 5DD, UK

michaela.smith@alumni.york.ac.uk

For peer review only - http://bmjopen.bmj.com/site/about/guidelines.xhtml

ABSTRACT

Objectives To examine whether there are associations between active travel and markers of a healthy, low-carbon diet (increased consumption of fruit and vegetables, reduced consumption of red and processed meat).

Design Cross-sectional analysis of a cohort study.

Settings Population cohort of over 500,000 people recruited from 22 centres across the United Kingdom. Participants aged between 40 and 69 years were recruited between 2006 and 2010.

Participants 412,299 adults with complete data on travel mode use, consumption of fruit and vegetables and red and processed meat, and socio-demographic covariates were included in the analysis.

Exposure measures Mutually exclusive mode or mode combinations of travel (car, public transport, walking, cycling) for non-work and commuting journeys.

Outcome measures Consumption of fruit and vegetables (FV) measured as portions per day and red and processed meat (RPM) measured as frequency per week.

Results Engaging in all types of active travel was positively associated with higher FV consumption and negatively associated with more frequent RPM consumption. Cycling exclusively or in combination with walking was most strongly associated with increased dietary consumption of FV and reduced consumption of RPM for both non-work and commuting journeys. Overall, the strongest associations were between non-work cycling and FV consumption (males: adjusted odds ratio=2.18, 95% confidence interval=2.06, 2.30; females: 2.50, 2.31, 2.71) and non-work cycling and RPM consumption (males: 0.57, 0.54, 0.60; females: 0.54, 0.50, 0.59). Associations were generally similar for both commuting and non-work travel, and were robust to adjustment with socio-demographic and behavioural factors.

Conclusions There are strong associations between engaging in active travel, particularly cycling, and healthy, low-carbon (HLC) dietary consumption, suggesting that these HLC behaviours are related. Further research is needed to better understand the drivers and dynamics between these behaviours within individuals, and whether they share common underlying causes.

For peer review only - http://bmjopen.bmj.com/site/about/guidelines.xhtml

Article summary

Strengths and limitations of this study

- This study uses the UK Biobank to examine associations between active travel and diet in order to better understand the patterning of healthy, low-carbon lifestyles.
- UK Biobank is a large cohort with very rich data, which enabled assessment of relationships using several measures of travel and dietary behaviour and adjustment for a wide range of socio-demographic, environmental, and behavioural covariates.
- The analysis used in this study is cross-sectional and therefore we cannot infer causality between these behaviours.
- This study is further limited by the use of self-reported measures of active travel and dietary consumption.

For peer review only

For peer review only - http://bmjopen.bmj.com/site/about/guidelines.xhtml

INTRODUCTION

Increasing concerns about global climate change combined with rising rates of chronic disease have led to greater policy attention on behaviours and lifestyles that are beneficial for both human health and the natural environment [1-3]. From this perspective, two priority behaviours that have been identified are engaging in healthy, low-carbon travel (e.g. walking and cycling for transport) and consuming healthy, low-carbon diets (e.g. reduced consumption of meat, increased consumption of fruit and vegetables) [4-8]. Modelling studies have shown that a population shift toward these behaviours would lead to a range of health and environmental co-benefits: increased life expectancies, decreased rates of Type 2 diabetes, cardiovascular diseases and cancer, as well as large reductions in transport and food-related greenhouse gas emissions [3 6 7 9-12]. These shifts are also in line with national health guidelines. In the UK, for example, walking and cycling for transport is widely recommended as a key strategy to increase population physical activity [13 14], and adults are advised to base two-thirds of their diet on plant-sourced foods, specifically consuming at least 5 portions of fruit and vegetables (FV) and less than 70 grams (g) of red and processed meat (RPM) per day to prevent chronic disease outcomes [15 16]. These dietary principles are also in alignment with the recently published 'planetary health diet' which argues that huge changes in consumption of fruits, vegetables, and red meat are needed on a global scale if we are to stay within safe planetary boundaries [17].

In the UK, studies that have examined these travel and dietary behaviours at the population level have found that they are strongly patterned by socio-demographic factors [18-22], which suggests that healthy, low-carbon (HLC) behaviours may overlap among certain population groups and/or within specific environments. Nevertheless, it remains unclear whether these behaviours actually co-occur within the same individuals, as there are very few studies that have examined HLC travel and dietary behaviours in relation to each other. For example, evidence from surveys and psychological research has shown that people who are willing to drive less (or drive more efficiently) are also more willing to reduce their meat consumption, but these associations have been limited to behavioural intentions

For peer review only

For peer review only - http://bmjopen.bmj.com/site/about/guidelines.xhtml

rather than actual travel behaviour and food consumption [23-25]. At the same time, there is considerable evidence of positive associations between physical activity and consuming more healthful diets [26-29], but it is not known whether this relationship also extends to forms of physically active *travel* or to diets that are both healthy and low-carbon. Based on this evidence, it has been proposed that strategies to promote active travel could also offer additional population health benefits through indirect dietary outcomes [30], but these relationships are poorly understood and have not yet been tested empirically. Determining whether behaviours co-occur is important because if behaviours are related, engaging in one behaviour may modify the likelihood of engaging in another [31-34]. This means that strategies which target multiple behaviours together may have additional benefits over the sum of individual interventions [35], and therefore have the potential to produce synergistic outcomes. Indeed, this potential for positive interactions makes it particularly important to tease out relationships between active travel and dietary consumption, as it is possible that the observed associations between walking/cycling and better health outcomes in the literature may be partially attributable to the dietary patterns of active travellers (and/or vice versa).

In light of these gaps, the objective of this study was to explore relationships between HLC behaviours in the travel and dietary domains, by examining associations between engaging in active travel and consumption of two food groups (FV, RPM) that have contrasting implications for human health and carbon emissions. Our choice of measures was based on the behaviours for which there are UK government recommendations and for which there is the greatest evidence of combined public health and environment benefits. As far as we are aware, there has been no prior research that has explicitly examined the relationships between these combinations of diet and travel behaviour.

For peer review only - http://bmjopen.bmj.com/site/about/guidelines.xhtml

METHODS

Study design and sample

We used baseline data from UK Biobank (project 14840) to assess cross-sectional relationships between use of different travel modes and dietary consumption. The scientific rationale, study design, and survey methods for UK Biobank (UKB) have been described elsewhere [36]. Briefly, data were collected from 502,616 individuals aged 40–69 years recruited between 2006 and 2010. Participants were identified from National Health Service patient registers and invited to attend one of 22 assessment centres located throughout the UK. At each assessment centre, participants completed a touchscreen questionnaire that collected information on socio-demographic characteristics and diet, lifestyle, and environmental factors. UKB received ethics approval from the National Information Governance Board for Health and Social Care and the National Health Service North West Centre for Research Ethics Committee (Ref: 11/NW/0382).

In this study, participants who did not provide any information on travel mode use (n=7,272) or dietary consumption (FV or RPM, n=1,820) were excluded, yielding an initial sample size of 493,524. This number was then further restricted to participants who had complete data on all analytical covariates (n=412,299 for all journeys, n=234,148 for commuting journeys). Sensitivity analyses were conducted with a further subsample that had complete data on weekly physical activity (PA) and total energy intake (95,475 females and 83,213 males).

Measures

Travel mode use

Data on travel behaviour were collected on the touchscreen questionnaire. Participants were asked to report which travel mode(s) they used for non-work journeys (*In the last 4 weeks, which forms of transport have you used most often to get about?*) and for their travel to work (commuting journeys), if they were currently employed and did not always work from home (*What types of transport do you use*

For peer review only

For peer review only - http://bmjopen.bmj.com/site/about/guidelines.xhtml

to get to and from work?). Both questions had the same response options (car/motor vehicle, public transport, walking, cycling), and allowed participants to select multiple modes for each type of journey.

Using these two questions, we categorised travel behaviour in several ways. First, to create an overall measure of active travel for each participant, we combined the responses from the two travel questions into one binary variable which included those who reported any walking or any cycling for either non-work or commuting journeys. Similar binary variables were also created for any walking and any cycling across the two types of journeys. Second, to account for all possible combinations of travel, a 15-category travel mode variable was derived for each type of journey (non-work, commuting) in order to organize the modal combinations from those producing the most carbon emissions and requiring the least physical exertion (car use only), to those producing the least emissions and requiring the most physical exertion (cycling only or cycling + walking). This was then collapsed into an eight-category variable for each type of journey: (1) car only, (2) car + public transport only, (3) car + public and active transport, (4) car + active transport only, (5) public transport only, (6) public + active transport, (7) walking only, (8) cycling only or cycling + walking. This approach is similar to that used previously by Flint and Cummins [37].

Dietary consumption

Data on FV and RPM consumption also came from the touchscreen questionnaire. Participants were asked to report their FV consumption via four open-ended questions that asked about the average number of tablespoons of vegetables and pieces of fruit consumed each day. These responses were then recoded into standard ‘5-a-day’ portions [38] that resulted in an overall measure of average portions of FV consumed for each participant. To assess whether each participant’s consumption was in line with the recommended guideline, this variable was also recoded into a three-level ordinal measure: <3, 3 to <5, and 5+ portions/day.

For peer review only - http://bmjopen.bmj.com/site/about/guidelines.xhtml

Participants were asked five questions about their average weekly intake of different types of meat. To create an overall measure of RPM consumption, we combined the four questions involving RPM (beef, lamb, pork, processed meat) into a composite index, based on the number of times each type of meat was consumed on a weekly basis [39]. For each meat type, the responses were coded as follows: Never = 0, Less than once a week = 0.5, Once a week = 1, 2-4 times a week = 3, 5-6 times a week = 5.5, once or more daily = 7. This index variable ranged from 0 to 28, where 0 indicated that participants never consumed any RPM and 28 indicated that participants consumed all four types of RPM on a daily basis. Based on the distribution of the resulting index variable, RPM consumption was then grouped into three categories: (1) non-consumers, and consumers split at the median frequency: (2) up to 3 times per week, (3) >3 times per week. This approach was used by Bradbury, et al. [39], who showed that those who consume RPM most frequently (>3 times per week) in the UKB sample also consume the largest quantities per day.

Covariates

Various demographic, socioeconomic, and environmental factors were hypothesised as possible confounders to relationships between travel behaviour and dietary consumption. Demographic covariates were age at baseline, sex, ethnic origin, and household size. Socioeconomic covariates were gross annual household income, number of cars per household, highest educational qualification, and occupational class. We used the National Statistics Socioeconomic Classification for occupation class by converting codes from Standard Occupational Classification (SOC) 2000. Environmental covariates were residential area classification, Townsend deprivation score, and region of UK.

Weekly PA (meeting or not meeting PA guideline) and total energy intake (kcal) were used in sensitivity analyses, due to the complex interrelationships between active travel, physical activity, dietary consumption and energy intake (further details and diagram of putative relationships in Supplementary Appendix, Figure S1). Those who reported 150 minutes of moderate PA or 75 minutes of vigorous PA per week were considered to meet the current PA guideline [40]. Data on total energy intake came from

For peer review only - http://bmjopen.bmj.com/site/about/guidelines.xhtml

a 24-h dietary recall questionnaire which was completed at the assessment centre by the last 70,000 participants and up to four times by email in the rest of the cohort [41]. For respondents who completed multiple dietary recall questionnaires, we used the average value.

Covariates were mostly self-reported on the touchscreen questionnaire, with the exception of occupational class (verbal interview), residential area classification (census), Townsend deprivation score (census), region of UK (assessment centre location), and average energy intake (24-h dietary assessment).

Statistical Analysis

Associations between each measure of travel behaviour and each dietary outcome were examined using multivariate ordinal regression models in Stata/SE 14.0 [42]. We used ordinal logistic regression in order to model the trends in dietary consumption while keeping the 'extremes' as useful categories (e.g. non-consumers of RPM, and those who met or exceeded consumption guidelines). This enabled meaningful interpretation of the relationships with a view to national dietary recommendations and potentially discontinuous changes in the associations between travel and dietary behaviour. Though these relationships could plausibly go in either direction, we modelled them in this way based on previous hypotheses [30] as well as neurocognitive research which suggests that physical activity may be more likely to lead to dietary changes than vice versa [43 44].

In Model 1 we examined the bivariate association between each travel variable and each dietary outcome and in Model 2 we adjusted for socio-demographic and environmental covariates. As a sensitivity analysis we further adjusted for physical activity and energy intake (Model 3) in the subsample with complete data on these factors (for comparison purposes Models 1 and 2 were re-run in this subsample as well). This sensitivity analysis was only conducted for the any active travel variable since this contained all of the other active travel combinations.

When interpreting the ordinal logistic model, the model assumes that the relationship between each

For peer review only - http://bmjopen.bmj.com/site/about/guidelines.xhtml

For peer review only

pair of outcome groups is the same, or in other words, that the coefficients describing the relationship between the lowest outcome category and all higher categories are the same as those describing the relationship between the next lowest category and all higher categories, etc. This is called the proportional odds or parallel lines assumption [45], and in this case, the models assume that the odds of being in the lowest dietary consumption category compared to the two highest, are the same as the odds of being in the highest consumption category compared to the two lowest. In each regression model, we tested the proportional odds assumption using the Stata *oparallel* post-estimation command [46]. Where this assumption was not met (p<0.05), we re-ran each model as a generalised ordered logit model (Stata extension *gologit2*) which relaxes the proportional odds assumption for some predictor variables while maintaining it for others [47]. This approach has the advantages of being more parsimonious and interpretable than those estimated by a non-ordinal method and may also give added insights (e.g. discontinuous changes) into the data that would be lost by ignoring the differences and continuing to use the fully ordinal model [45]. We present odds ratios (OR) or adjusted odds ratios (aOR) with 95% confidence intervals (CI) and set a threshold of alpha=0.05 for statistical significance. All analyses were stratified by sex due to established gender differences in the patterning of travel behaviour and dietary consumption in the UK population [19 21 48 49].

Patient and Public Involvement

This study was conducted using the UK Biobank resource. Details of patient and public involvement in the UK Biobank are available online (https://www.ukbiobank.ac.uk/public-consultation/). No patients were specifically involved in setting the research question or the outcome measures, nor were they involved in developing plans for recruitment, design or implementation of this study. No patients were asked to advise on interpretation or writing up of results. There are no specific plans to disseminate the results of the research to study participants, but the UK Biobank disseminates key findings from projects on its website (https://www.ukbiobank.ac.uk/participant-events/).

For peer review only - http://bmjopen.bmj.com/site/about/guidelines.xhtml

RESULTS

Descriptive characteristics of the sample are presented in Table 1. As well as being older, UK Biobank participants are more socioeconomically advantaged, and more health-conscious in comparison with the UK general population [50]. In this study 54.5% of the sample reported walking or cycling for either type of journey (any active travel), and walking was much more common than cycling (51.6% vs. 9.4%) (Table 2). Car only travel was much higher for commuting journeys (63.1%) than for non-work journeys (39.5%), indicating that people were more likely to use multiple modes and more active modes for non-work travel. For example, 22.0% of the sample mixed car use with active modes, and 14.0% mixed car, active modes and public transport use for non-work journeys. For diet, 58.3% of males and 36.7% of females reported consuming RPM more than three times per week and only 5.3% of the sample reported never consuming any RPM (3.4% among males, 7.0% among females) (Table 3). Nearly 38% reported consuming 5+ portions of FV per day on average (31.4% among males, 43.3% among females).

Associations between travel modes and FV consumption

Across all models, there were positive associations between all types of HLC travel and FV consumption among both males and females, with very little change even after adjustment for demographic, socioeconomic, and environmental factors (Table 4 and Supplementary Appendix, Figure S2). Associations were generally much stronger for cycling than for other travel modes. For example, in the fully adjusted models (Model 2), men and women who engaged in any cycling travel were nearly twice as likely to consume higher amounts of FV than those who did not cycle for transport (males: aOR=1.65, 95%CI 1.61, 1.69; females: aOR=1.67, 95%CI 1.62, 1.73). Across the more detailed travel classifications of non-work and commuting journeys, associations were generally weaker or non-significant for travel that did not involve any walking or cycling (e.g. car + public transport, public transport only). Comparing across the two types of journeys, the associations were fairly similar in magnitude, though they were slightly stronger for non-work travel, and particularly for non-work cycling.

For peer review only - http://bmjopen.bmj.com/site/about/guidelines.xhtml

Based on the confidence intervals, women who engaged in any active travel (aOR=1.43, 95%CI 1.40, 1.45) or any walking travel (aOR=1.38, 95%CI 1.36, 1.41) were more likely to consume higher amounts of FV compared with males (aOR=1.35, 95%CI 1.33, 1.37 and aOR=1.25, 95%CI 1.23, 1.27, respectively). For non-work journeys, the same was also true for women who used car + active travel, walking only, and cycling / cycling + walking, compared with their male counterparts. Full models are shown in Supplementary Appendix, Tables S1 and S2.

Associations between travel modes and RPM consumption

Overall, the associations between HLC travel and RPM consumption were nearly all negative; the only exception was for car + public transport (versus car only travel) among females for non-work journeys (Table 5 and Supplementary Appendix, Figure S3). Among both males and females, associations were only slightly attenuated with adjustment for demographic, socioeconomic, and environmental factors. As with FV consumption, these associations were strongest for cycling, overall and across both types of journeys. Moreover, there was a clear gradient of effect for non-work travel, such that the more active the travel mode(s), the more negative the association with RPM consumption frequency. For example, in the fully adjusted models (Model 2), men and women who cycled for non-work journeys were nearly half as likely to consume RPM more frequently than those who travelled by car (males: aOR=0.57; 95%CI 0.54, 0.60; females: aOR=0.54, 95%CI 0.50, 0.59). Full models are shown in Supplementary Appendix, Tables S3 and S4.

Proportional odds assumption

Due to the very large sample size in UKB, we were able to detect very minor variations in the data, and this meant that all of the models in Tables 4 and 5 had violations of the proportional odds assumption. To assess whether these differences were meaningful for the key variables of interest (travel variables), all of the models were re-run using a generalised ordered logit model (Supplementary Appendix, Tables S5 and S6). Here the associations were generally of similar magnitude and in the same direction to the

For peer review only - http://bmjopen.bmj.com/site/about/guidelines.xhtml

fully ordinal models, but where differences were present, the associations tended to be slightly stronger for the two highest categories versus the lowest category of the outcome variables, for example, 3+ portions of FV versus <3, and RPM consumers versus never consumers. This relatively trivial difference does not alter the directions of the associations (positive and negative) in our main findings.

Sensitivity analyses

In the subset of the sample with full data on energy intake and physical activity (n=95,475 females, n=83,213 males), adjusting for these variables in addition to the other socio-demographic and environmental factors slightly attenuated the associations between any active travel and FV consumption, but the relationship was still independent and highly significant among both males and females (males: aOR=1.28; 95%CI 1.24, 1.31 and females: aOR=1.35, 95%CI 1.32, 1.39) (Supplementary Appendix, Tables S7 and S8). Similarly, the associations between any active travel and RPM consumption were also very slightly attenuated, but even less so than for FV consumption (males: aOR= 0.89; 95%CI 0.87, 0.92 and females: aOR=0.90, 95%CI 0.88, 0.92) (Supplementary Appendix, Tables S9 and S10).

DISCUSSION

To our knowledge, this is the first analysis to explicitly examine relationships between engaging in active travel and HLC dietary consumption, thus beginning to clarify the patterning of HLC lifestyles. We have shown that engaging in active travel, and in particular cycling, is associated with increased consumption of FV and with reduced consumption of RPM in the UKB sample. These associations were robust to adjustment by both socio-demographic and behavioural factors, suggesting that these factors do not explain the observed relationships. Using multiple measures of travel and dietary behaviour, we have assessed these relationships comprehensively across different travel modes, types of journeys, and relevant food groups, and also adjusted for a wide range of important covariates. This level of detail has

For peer review only - http://bmjopen.bmj.com/site/about/guidelines.xhtml

For peer review only

allowed us to isolate and elucidate where the relationships between these HLC behaviours are strongest and weakest, which is an important contribution to understanding which elements of travel and dietary behaviour may share common underlying factors.

The major strength of this study is the large sample size and flexible measures of travel behaviour in the UKB dataset, both of which enabled the observation of relatively fine-grained differences in the data. Nevertheless, UKB is limited by its lack of representativeness, as it is based on a sample of 'healthy volunteers' [50] and excludes large segments of the population (e.g. those under age 40). The data were also collected between 2006 and 2010, and there have been some population changes in meat consumption since then, though less so among those in the UKB age range [51]. As a result, it is unclear whether these results are generalizable to the UK general population, however similar relationships were also found when this analysis was replicated in a nationally representative UK sample [52 53], which supports the external validity of these associations.

Other limitations include that the measures used were all self-reported and that the analyses are cross-sectional. Due to the health-conscious nature of the cohort, it is possible that consumption of some food groups may be over- or under-reported, however, an in-depth study of the reliability of the UKB touchscreen dietary questionnaire has shown that participant responses for FV and meat consumption are very consistent over time (70-90%) and correlate well with other independent dietary assessments (e.g. 24 hour dietary recall) conducted as part of the larger UKB study [54]. Nonetheless, if participants were more likely to report that they walked, cycled, ate more FV and ate less RPM, then this might partially explain the observed associations between these behaviours. The cross-sectional nature of the data means we cannot establish causality between these behaviours in terms of whether active travel precedes higher FV and lower RPM consumption, vice versa, or whether change occurs in tandem, or when in the life course such patterns emerge or change. Future research with longitudinal data will help to confirm the direction of these relationships, as well as improve our understanding of behaviour dynamics over time.

For peer review only - http://bmjopen.bmj.com/site/about/guidelines.xhtml

Importantly, the findings of this study confirm much of the wider evidence on links between health and environmental behaviours, and represent some of the strongest evidence to date on this topic. Several studies have reported clustering between increased physical activity and more nutritious diets [26-29 55], but this study is the first to show that comparable associations exist for *physically active travel* and healthy diets, independent of overall physical activity. Our findings also build upon studies of environmental behaviours which have linked reduced car driving with reduced meat consumption, but which have only measured behavioural intentions [24 25]. More indirectly, there are also interesting parallels between this study and the growing body of evidence relating active travel, and particularly active commuting, to positive health outcomes like lower obesity and reduced mortality [19 37 56-59]. Two of these studies, also conducted using UKB but only examining active commuting, have found particularly strong effects for cycling to work and lower obesity [37] and reduced mortality [58], far over and above the effects found for walking. Combining these findings with our results on the dietary patterns of cyclists suggests that positive interactions between cycling travel and HLC diets could be one factor contributing to the enhanced health effects observed among individuals who cycle.

This study has several important implications. Firstly, the results suggest that active travel and HLC diets may be related and share similar determinants within individuals. Theoretical understandings of behavioural co-occurrence suggest that behaviours which cluster together share common causal pathways [31 32], and that the stronger the relationship between two behaviours, the more determinants they are likely to share [60]. In this study, strong relationships were seen most clearly between cycling and FV consumption, even after adjusting for socio-demographic characteristics and behavioural factors like overall physical activity and energy intake. This suggests that these behaviours may be driven by common underlying factors, and supports the interpretation of both behaviours being related to health motivations, though there may also be other factors at play. Since cycling is still a relatively rare form of travel in the UK, these patterns may reflect the fact that people who cycle for transport are somewhat unique, and may also deviate from social norms in other ways (e.g. diet). Future research could explore this area further by examining whether relationships between cycling and dietary

For peer review only - http://bmjopen.bmj.com/site/about/guidelines.xhtml

consumption are consistent in parts of the UK where people cycle at higher frequencies (e.g. Cambridge) [61] or among those who cycle at higher intensities, such as for sport.

Identifying whether two behaviours are related is important because strongly associated behaviours may influence each other in different ways [31-34]. In the case of positive relationships, this could mean that related behaviours have the potential to produce synergistic outcomes, if strategies that target multiple HLC behaviours together have greater benefits than the sum of individual interventions [35]. Urgent changes in lifestyles are needed if we are to avoid catastrophic climate change [62 63]. Putting these changes into action requires that we have a complete understanding of people's behaviour patterns, including how different behaviours influence, interact and intersect with one another across the life course. Though relationships between active travel and diet still need to be examined longitudinally, this study suggests that these HLC behaviours may have the potential to positively influence one another, and that promotion of these behaviours could help foster enhanced benefits for both human health and the natural environment.

For peer review only

For peer review only - http://bmjopen.bmj.com/site/about/guidelines.xhtml

Contributors

MAS, JRB, HG, PCLW and SLP made substantial contributions to the conception and design of the study and interpretation of data. MAS undertook the statistical analysis with input from JRB and SLP. MAS drafted the article and JRB, HG, PCLW and SLP revised it critically for important intellectual content. MAS, JRB, HG, PCLW and SLP approved the final version of the manuscript to be published.

Funding

MAS was supported by a PhD studentship from the University of York as part of the Health of Populations and Ecosystems (HOPE) project, funded by the Economic and Social Research Council (grant number ES/L003015/1), awarded to HG and PCLW.

Acknowledgement

This research has been conducted using the UK Biobank Resource under Application Number 14840.

Competing interests

None declared.

Data sharing statement

This study used data from the UK Biobank (www.ukbiobank.ac.uk) which does not permit public sharing of the data. The data are, however, open to all qualified researchers anywhere in the world and can be accessed by applying through the UK Biobank Access Management System (www.ukbiobank.ac.uk/register-apply).

For peer review only

Licence statement

I, the Submitting Author has the right to grant and does grant on behalf of all authors of the Work (as defined in the below author licence), an exclusive licence and/or a non-exclusive licence for contributions from authors who are: i) UK Crown employees; ii) where BMJ has agreed a CC-BY licence shall apply, and/or iii) in accordance with the terms applicable for US Federal Government officers or employees acting as part of their official duties; on a worldwide, perpetual, irrevocable, royalty-free basis to BMJ Publishing Group Ltd ("BMJ") its licensees and where the relevant Journal is co-owned by BMJ to the co-owners of the Journal, to publish the Work in BMJ Open and any other BMJ products and to exploit all rights, as set out in our licence.

The Submitting Author accepts and understands that any supply made under these terms is made by BMJ to the Submitting Author unless you are acting as an employee on behalf of your employer or a postgraduate student of an affiliated institution which is paying any applicable article publishing charge ("APC") for Open Access articles. Where the Submitting Author wishes to make the Work available on an Open Access basis (and intends to pay the relevant APC), the terms of reuse of such Open Access shall be governed by a Creative Commons licence – details of these licences and which Creative Commons licence will apply to this Work are set out in our licence referred to above.

For peer review only - http://bmjopen.bmj.com/site/about/guidelines.xhtml

For peer review only

1 **Table 1 – Descriptive characteristics of sample (n=412,299)**

2

	Males		Females		All	
	n	%	n	%	n	%
Total	195,131	47.3	217,168	52.7	412,299	100.0
Age at baseline (years) [a]						
38-44	20,476	10.5	23,892	11.0	44,368	10.8
45-49	25,246	12.9	31,543	14.5	56,789	13.8
50-54	28,821	14.8	36,394	16.8	65,215	15.8
55-59	34,774	17.8	40,910	18.8	75,684	18.4
60-64	46,955	24.1	50,174	23.1	97,129	23.6
65-73	38,859	19.9	34,255	15.8	73,114	17.7
Ethnic group						
White British	175,294	89.8	193,220	89.0	368,514	89.4
Other White	10,855	5.6	13,903	6.4	24,758	6.0
South Asian	3,835	2.0	2,870	1.3	6,705	1.6
Black	2,403	1.2	3,306	1.5	5,709	1.4
Chinese	450	0.2	720	0.3	1,170	0.3
Mixed	891	0.5	1,448	0.7	2,339	0.6
Other	1,403	0.7	1,701	0.8	3,104	0.8
Highest qualification [b]						
College or University degree	70,136	35.9	74,613	34.4	144,749	35.1
A levels or equivalent	20,898	10.7	27,183	12.5	48,081	11.7
GCSEs or equivalent	36,862	18.9	51,055	23.5	87,917	21.3
CSEs or equivalent	10,560	5.4	11,730	5.4	22,290	5.4
NVQ or HND or HNC or equivalent	17,732	9.1	9,607	4.4	27,339	6.6
Other professional qualifications	8,560	4.4	12,375	5.7	20,935	5.1
No qualifications	30,383	15.6	30,605	14.1	60,988	14.8
Occupational Class [c]						
Higher managerial & professional	48,981	25.1	25,058	11.5	74,039	18.0
Lower managerial & professional	34,686	17.8	54,458	25.1	89,144	21.6
Intermediate occupations	14,933	7.7	36,723	16.9	51,656	12.5
Small employers & own accounts	9,345	4.8	4,958	2.3	14,303	3.5
Lower supervisory & technical	10,702	5.5	1,019	0.5	11,721	2.8
Semi-routine occupations	10,986	5.6	20,181	9.3	31,167	7.6
Routine occupations	10,162	5.2	5,365	2.5	15,527	3.8
Not classified	55,336	28.4	69,406	32.0	124,742	30.3
Household income (before tax)						
Less than £18,000	39,184	20.1	52,863	24.3	92,047	22.3
£18,000 to 30,999	47,701	24.5	57,347	26.4	105,048	25.5
£31,000 to 51,999	52,674	27.0	55,578	25.6	108,252	26.3
£52,000 to 100,000	43,674	22.4	40,867	18.8	84,541	20.5
Greater than £100,000	11,898	6.1	10,513	4.8	22,411	5.4

For peer review only

For peer review only - http://bmjopen.bmj.com/site/about/guidelines.xhtml

Table 1 (continued)

	Males		Females		All	
	n	%	n	%	n	%
Household size						
1	33,345	17.1	45,334	20.9	78,679	19.1
2	90,130	46.2	98,297	45.3	188,427	45.7
3	30,803	15.8	33,989	15.7	64,792	15.7
4	29,408	15.1	28,809	13.3	58,217	14.1
5+	11,445	5.9	10,739	4.9	22,184	5.4
Number of cars per household						
0	14,877	7.6	19,055	8.8	33,932	8.2
1	77,536	39.7	95,160	43.8	172,696	41.9
2	79,161	40.6	79,599	36.7	158,760	38.5
3	17,829	9.1	17,994	8.3	35,823	8.7
4+	5,728	2.9	5,360	2.5	11,088	2.7
Region [d]						
London	25,333	13.0	30,273	13.9	55,606	13.5
South East England	17,007	8.7	19,402	8.9	36,409	8.8
South West England	16,764	8.6	19,613	9.0	36,377	8.8
East Midlands	13,120	6.7	14,559	6.7	27,679	6.7
West Midlands	18,383	9.4	18,020	8.3	36,403	8.8
Yorkshire and the Humber	29,615	15.2	32,479	15.0	62,094	15.1
North East England	23,110	11.8	25,606	11.8	48,716	11.8
North West England	29,599	15.2	31,717	14.6	61,316	14.9
Wales	8,265	4.2	9,048	4.2	17,313	4.2
Scotland	13,935	7.1	16,451	7.6	30,386	7.4
Urban residence	167,547	85.9	186,617	85.9	354,164	85.9
Townsend score (mean, sd) [e]	-1.37	3.1	-1.37	3.0	-1.37	3.0

[a] Continuous variable in models

[b] A levels: academic advanced-levels, post compulsory education; GCSEs: academic General Certificate of Secondary Education, formerly Ordinary Levels, taken at age 15–16 years and the end of compulsory education ; CSEs: vocational Certificate of Secondary Education, formerly taken at age 15–16 years; NVQ, HND, HNC: National Vocational Qualifications, Higher National Diploma, Higher National Certificate, all intermediate semi-vocational qualifications

[c] Based on National Statistics Socio-economic Classification (NS-SEC), where Not classified = those who were retired, unemployed, looking after home/family, unable to work because of sickness/disability, doing unpaid/voluntary work, or full-time students

[d] Grouped based on assessment centre: London = St Barts, Croydon, Hounslow; South East England = Oxford, Reading; South West England = Bristol; East Midlands = Nottingham; West Midlands = Birmingham; Yorkshire and the Humber = Leeds, Sheffield; North East England = Middlesbrough, Newcastle; North West England = Liverpool, Manchester, Bury; Wales = Cardiff, Swansea, Wrexham; Scotland = Glasgow, Edinburgh.

[e] Lower score = less deprived (min: -6.3; max: 11.0)

For peer review only

Table 2 – Descriptive overview of travel mode use (n=412,299)

	Males (n=195,131)		Females (n=217,168)		All (n=412,299)	
	n	%	n	%	n	%
Any active travel [a]	105,287	54.0	119,244	54.9	224,531	54.5
Any walking travel	96,976	49.7	115,573	53.2	212,549	51.6
Any cycling travel	24,806	12.7	13,877	6.4	38,683	9.4
Non-work journeys [b]						
Car only	79,582	40.9	82,980	38.3	162,562	39.5
Car + PT	6,058	3.1	8,822	4.1	14,880	3.6
Car + mixed (PT and AT)	25,683	13.2	32,024	14.8	57,707	14.0
Car + AT	44,488	22.8	46,117	21.3	90,605	22.0
PT only	9,957	5.1	13,277	6.1	23,234	5.6
PT + AT	11,793	6.1	16,020	7.4	27,813	6.8
Walking only	12,553	6.5	14,939	6.9	27,492	6.7
Cycling / cycling + walking	4,660	2.4	2,648	1.2	7,308	1.8
Missing	357		341		698	
Commuting journeys [c]						
Car only	74,043	65.5	73,736	60.9	147,779	63.1
Car + PT	6,735	6.0	7,519	6.2	14,254	6.1
Car + mixed (PT and AT)	3,649	3.2	3,578	3.0	7,227	3.1
Car + AT	7,573	6.7	8,727	7.2	16,300	7.0
PT only	8,383	7.4	12,042	10.0	20,425	8.7
PT + AT	4,861	4.3	5,081	4.2	9,942	4.3
Walking only	3,878	3.4	8,183	6.8	12,061	5.2
Cycling / cycling + walking	3,964	3.5	2,196	1.8	6,160	2.6
Missing / not applicable	82,045		96,106		178,151	

PT: public transport; AT: active travel

[a] Includes walking or cycling for non-work or commuting travel

[b] n=411,601 for non-work travel (0.2% missing), % are calculated from non-missing

[c] n=234,148 for commuting travel (43.2% missing/not applicable), % are calculated from non-missing

For peer review only - http://bmjopen.bmj.com/site/about/guidelines.xhtml

Table 3 – Descriptive overview of dietary consumption and physical activity (n=412,299)

	Males (n=195,131)		Females (n=217,168)		All (n=412,299)	
	n	%	n	%	n	%
FV consumption (portions/day)						
< 3	66,672	34.2	45,669	21.0	112,341	27.3
3 to < 5	67,263	34.5	77,583	35.7	144,846	35.1
5+	61,196	31.4	93,917	43.3	155,112	37.6
RPM consumption (frequency/week)						
Never	6,615	3.4	15,250	7.0	21,865	5.3
≤ 3 times	74,766	38.3	122,148	56.3	196,914	47.8
> 3 times	113,750	58.3	79,770	36.7	193,520	46.9
Meets physical activity guideline [a]						
Yes	101,323	54.1	103,804	50.0	205,127	52.0
No	86,112	45.9	103,996	50.0	189,108	48.0
Missing	7,696		10,368		18,064	
Total energy intake, kcal/day (mean, sd) [b]	2,299	685	1,971	575	2,123	649

[a] n=394,235 for physical activity guideline (4.4% missing), % are calculated from non-missing

[b] Based on n=98,853 females, n=85,392 males

For peer review only

Table 4 – Ordinal logistic models between HLC travel and FV consumption, stratified by gender (n=412,299)

	Males (n=195,131)		Females (n=217,168)	
TRAVEL VARIABLES	Model 1 [a]	Model 2 [b]	Model 1 [a]	Model 2 [b]
	OR (95% CI)			
Any active travel (ref: None)	1.37*** (1.34 - 1.39)	1.35*** (1.33 - 1.37)	1.42*** (1.40 - 1.44)	1.43*** (1.40 - 1.45)
Any walking (ref: None)	1.28*** (1.26 - 1.31)	1.25*** (1.23 - 1.27)	1.38*** (1.36 - 1.40)	1.38*** (1.36 - 1.41)
Any cycling (ref: None)	1.57*** (1.54 - 1.61)	1.65*** (1.61 - 1.69)	1.58*** (1.53 - 1.63)	1.67*** (1.62 - 1.73)
Non-work travel [c] (ref: Car only)				
Car + public transport	1.06* (1.01 - 1.11)	1.00 (0.95 - 1.05)	1.05* (1.01 - 1.09)	0.98 (0.94 - 1.02)
Car + mixed (public and active)	1.49*** (1.46 - 1.53)	1.37*** (1.33 - 1.40)	1.57*** (1.53 - 1.61)	1.41*** (1.38 - 1.45)
Car + active travel	1.27*** (1.24 - 1.29)	1.26*** (1.24 - 1.29)	1.37*** (1.34 - 1.40)	1.39*** (1.36 - 1.42)
Public transport only	1.03 (0.99 - 1.07)	1.13*** (1.08 - 1.18)	1.03 (0.99 - 1.06)	1.11*** (1.06 - 1.15)
Public transport + active travel	1.31*** (1.27 - 1.36)	1.43*** (1.37 - 1.49)	1.45*** (1.40 - 1.50)	1.52*** (1.47 - 1.58)
Walking only	1.34*** (1.29 - 1.38)	1.39*** (1.34 - 1.44)	1.47*** (1.42 - 1.52)	1.57*** (1.51 - 1.62)
Cycling / cycling + walking	2.06*** (1.95 - 2.17)	2.18*** (2.06 - 2.30)	2.34*** (2.17 - 2.52)	2.50*** (2.31 - 2.71)
Commuting travel [d] (ref: Car only)				
Car + public transport	1.03 (0.98 - 1.08)	0.97 (0.92 - 1.01)	1.02 (0.97 - 1.06)	0.99 (0.95 - 1.03)
Car + mixed (public and active)	1.44*** (1.36 - 1.53)	1.37*** (1.29 - 1.46)	1.45*** (1.36 - 1.54)	1.41*** (1.32 - 1.50)
Car + active travel	1.41*** (1.35 - 1.47)	1.47*** (1.41 - 1.54)	1.23*** (1.18 - 1.28)	1.30*** (1.24 - 1.35)
Public transport only	1.08*** (1.03 - 1.12)	1.03 (0.98 - 1.08)	0.91*** (0.88 - 0.95)	0.95* (0.91 - 0.99)
Public transport + active travel	1.41*** (1.33 - 1.48)	1.37*** (1.29 - 1.45)	1.26*** (1.19 - 1.32)	1.28*** (1.20 - 1.35)
Walking only	1.20*** (1.13 - 1.28)	1.24*** (1.16 - 1.32)	1.07*** (1.03 - 1.12)	1.20*** (1.14 - 1.25)
Cycling / cycling + walking	1.78*** (1.68 - 1.89)	1.82*** (1.71 - 1.93)	1.93*** (1.77 - 2.09)	2.00*** (1.84 - 2.18)

*** p<0.001, ** p<0.01, * p<0.05

[a] Model 1: unadjusted

[b] Model 2: adjusted for age, ethnic group, education, occupational class, household income, household size, number of cars, assessment centre location, population density, Townsend score (Full models in Supplementary Appendix)

[c] n=194,775 males, n=216,828 females

[d] n=113,087 males, n=121,063 females

For peer review only - http://bmjopen.bmj.com/site/about/guidelines.xhtml

Table 5 – Ordinal logistic models between HLC travel and RPM consumption, stratified by gender (n=412,299)

TRAVEL VARIABLES	Males (n=195,131)		Females (n=217,168)	
	Model 1 [a]	Model 2 [b]	Model 1 [a]	Model 2 [b]
	OR (95% CI)			
Any active travel (ref: None)	0.87*** (0.85 - 0.88)	0.89*** (0.87 - 0.91)	0.85*** (0.84 - 0.87)	0.88*** (0.87 - 0.90)
Any walking (ref: None)	0.91*** (0.89 - 0.93)	0.94*** (0.92 - 0.95)	0.88*** (0.86 - 0.89)	0.91*** (0.89 - 0.92)
Any cycling (ref: None)	0.75*** (0.73 - 0.77)	0.76*** (0.74 - 0.78)	0.67*** (0.65 - 0.69)	0.72*** (0.69 - 0.74)
Non-work travel [c] (ref: Car only)				
Car + public transport	0.99 (0.94 - 1.04)	1.01 (0.95 - 1.06)	1.12*** (1.07 - 1.17)	1.09*** (1.04 - 1.14)
Car + mixed (public and active)	0.92*** (0.89 - 0.94)	0.96* (0.94 - 0.99)	0.93*** (0.91 - 0.96)	0.95*** (0.93 - 0.98)
Car + active travel	0.95*** (0.93 - 0.98)	0.96*** (0.94 - 0.98)	0.94*** (0.92 - 0.96)	0.94*** (0.92 - 0.97)
Public transport only	0.91*** (0.87 - 0.95)	0.89*** (0.85 - 0.94)	0.87*** (0.84 - 0.90)	0.88*** (0.84 - 0.91)
Public transport + active travel	0.78*** (0.75 - 0.81)	0.77*** (0.74 - 0.81)	0.71*** (0.69 - 0.74)	0.76*** (0.73 - 0.79)
Walking only	0.76*** (0.73 - 0.78)	0.75*** (0.72 - 0.78)	0.70*** (0.68 - 0.72)	0.71*** (0.69 - 0.74)
Cycling / cycling + walking	0.56*** (0.53 - 0.59)	0.57*** (0.54 - 0.60)	0.50*** (0.46 - 0.54)	0.54*** (0.50 - 0.59)
Commuting travel [d] (ref: Car only)				
Car + public transport	0.94** (0.89 - 0.98)	1.00 (0.95 - 1.06)	1.00 (0.96 - 1.05)	1.04 (0.99 - 1.09)
Car + mixed (public and active)	0.82*** (0.76 - 0.87)	0.89** (0.84 - 0.96)	0.83*** (0.78 - 0.89)	0.93* (0.86 - 0.99)
Car + active travel	0.82*** (0.78 - 0.86)	0.83*** (0.79 - 0.87)	0.92*** (0.88 - 0.96)	0.89*** (0.85 - 0.93)
Public transport only	0.86*** (0.82 - 0.90)	0.95 (0.91 - 1.01)	0.89*** (0.85 - 0.92)	0.97 (0.93 - 1.01)
Public transport + active travel	0.70*** (0.66 - 0.74)	0.79*** (0.74 - 0.84)	0.71*** (0.67 - 0.75)	0.84*** (0.79 - 0.89)
Walking only	0.76*** (0.71 - 0.81)	0.80*** (0.75 - 0.86)	0.89*** (0.85 - 0.93)	0.86*** (0.82 - 0.91)
Cycling / cycling + walking	0.58*** (0.54 - 0.62)	0.60*** (0.56 - 0.64)	0.51*** (0.46 - 0.55)	0.55*** (0.50 - 0.60)

*** p<0.001, ** p<0.01, * p<0.05

[a] Model 1: unadjusted

[b] Model 2: adjusted for age, ethnic group, education, occupational class, household income, household size, number of cars, assessment centre location, population density, Townsend score (Full models in Supplementary Appendix)

[c] n=194,775 males, n=216,828 females

[d] n=113,087 males, n=121,063 females

For peer review only - http://bmjopen.bmj.com/site/about/guidelines.xhtml

References

1. Watts N, Adger WN, Agnolucci P, et al. Health and climate change: policy responses to protect public health. The Lancet 2015;**386** (10006):1861 - 914 doi: 10.1016/s0140-6736(15)60854-6[published Online First: Epub Date]|.
2. Whitmee S, Haines A, Beyrer C, et al. Safeguarding human health in the Anthropocene epoch: report of The Rockefeller Foundation–Lancet Commission on planetary health. The Lancet 2015;**386**:1973–2028 doi: 10.1016/s0140-6736(15)60901-1[published Online First: Epub Date]|.
3. Haines A, McMichael AJ, Smith KR, et al. Public health benefits of strategies to reduce greenhouse-gas emissions: overview and implications for policy makers. The Lancet 2009;**374**(9707):2104-14
4. Woodcock J, Edwards P, Tonne C, et al. Public health benefits of strategies to reduce greenhouse-gas emissions: urban land transport. The Lancet 2009;**374**(9705):1930-43
5. Capon AG, Synnott ES, Holliday S. Urbanism, climate change and health: systems approaches to governance. New South Wales public health bulletin 2009;**20**(1-2):24-8
6. Friel S, Dangour AD, Garnett T, et al. Public health benefits of strategies to reduce greenhouse-gas emissions: food and agriculture. The Lancet 2009;**374**(9706):2016-25 doi: 10.1016/s0140-6736(09)61753-0[published Online First: Epub Date]|.
7. Aston LM, Smith JN, Powles JW. Impact of a reduced red and processed meat dietary pattern on disease risks and greenhouse gas emissions in the UK: a modelling study. BMJ Open 2012;**2**(5) doi: 10.1136/bmjopen-2012-001072[published Online First: Epub Date]|.
8. Lindsay G, Macmillan A, Woodward A. Moving urban trips from cars to bicycles: impact on health and emissions. Aust N Z J Public Health 2011;**35**(1):54-60 doi: 10.1111/j.1753-6405.2010.00621.x[published Online First: Epub Date]|.
9. Jarrett J, Woodcock J, Griffiths UK, et al. Effect of increasing active travel in urban England and Wales on costs to the National Health Service. The Lancet 2012;**379**(9832):2198-205 doi: 10.1016/s0140-6736(12)60766-1[published Online First: Epub Date]|.
10. Green R, Milner J, Dangour AD, et al. The potential to reduce greenhouse gas emissions in the UK through healthy and realistic dietary change. Climatic Change 2015 doi: 10.1007/s10584-015-1329-y[published Online First: Epub Date]|.
11. Milner J, Green R, Dangour AD, et al. Health effects of adopting low greenhouse gas emission diets in the UK. BMJ Open 2015;**5**(4):e007364 doi: 10.1136/bmjopen-2014-007364[published Online First: Epub Date]|.
12. Tainio M, Monsivais P, Jones NRV, et al. Mortality, greenhouse gas emissions and consumer cost impacts of combined diet and physical activity scenarios: a health impact assessment study. BMJ Open 2017;**7**(2):e014199 doi: 10.1136/bmjopen-2016-014199[published Online First: Epub Date]|.
13. DfT/DH. Active Travel Strategy. London: Department for Transport/Department of Health, 2010.
14. NICE. Walking and cycling: local measures to promote walking and cycling as forms of travel or recreation: National Institute for Health and Clinical Excellence, 2012.
15. NHS. Meat in your diet. Secondary Meat in your diet 2018. https://http://www.nhs.uk/live-well/eat-well/meat-nutrition/.
16. NHS. The Eatwell Guide. Secondary The Eatwell Guide 2019. https://http://www.nhs.uk/live-well/eat-well/the-eatwell-guide/.
17. Willett W, Rockström J, Loken B, et al. Food in the Anthropocene: the EAT–Lancet Commission on healthy diets from sustainable food systems. The Lancet 2019;**393**(10170):447-92
18. Hutchinson J, White PL, Graham H. Differences in the social patterning of active travel between urban and rural populations: findings from a large UK household survey. International journal of public health 2014;**59**(6):993-98 doi: 10.1007/s00038-014-0578-2[published Online First: Epub Date]|.

For peer review only - http://bmjopen.bmj.com/site/about/guidelines.xhtml

19. Laverty AA, Mindell JS, Webb EA, et al. Active travel to work and cardiovascular risk factors in the United Kingdom. Am J Prev Med 2013;**45**(3):282-8 doi: 10.1016/j.amepre.2013.04.012[published Online First: Epub Date]|.
20. Maguire ER, Monsivais P. Socio-economic dietary inequalities in UK adults: an updated picture of key food groups and nutrients from national surveillance data. The British journal of nutrition 2014:1-9 doi: 10.1017/S0007114514002621[published Online First: Epub Date]|.
21. Aston LM, Smith JN, Powles JW. Meat intake in Britain in relation to other dietary components and to demographic and risk factor variables: analyses based on the National Diet and Nutrition Survey of 2000/2001. Journal of Human Nutrition and Dietetics 2013;**26**(1):96-106 doi: 10.1111/j.1365-277X.2012.01278.x[published Online First: Epub Date]|.
22. Leahy E, Lyons S, Tol RSJ. Determinants of vegetarianism and partial vegetarianism in the United Kingdom. Dublin: Economic and Social Research Institute (ESRI) working paper, 2010.
23. Van der Werff E, Steg L, Keizer K. I Am What I Am, by Looking Past the Present: The Influence of Biospheric Values and Past Behavior on Environmental Self-Identity. Environ Behav 2013;**46**(5):626-57 doi: 10.1177/0013916512475209[published Online First: Epub Date]|.
24. de Boer J, de Witt A, Aiking H. Help the climate, change your diet: A cross-sectional study on how to involve consumers in a transition to a low-carbon society. Appetite 2016;**98**:19-27 doi: 10.1016/j.appet.2015.12.001[published Online First: Epub Date]|.
25. Lee L, Simpson I. Are we eating less meat? A British Social Attitudes report. London: NatCen, 2016.
26. Noble N, Paul C, Turon H, et al. Which modifiable health risk behaviours are related? A systematic review of the clustering of Smoking, Nutrition, Alcohol and Physical activity ('SNAP') health risk factors. Prev Med 2015;**81**:16-41 doi: 10.1016/j.ypmed.2015.07.003[published Online First: Epub Date]|.
27. Poortinga W. The prevalence and clustering of four major lifestyle risk factors in an English adult population. Prev Med 2007;**44**(2):124-8 doi: 10.1016/j.ypmed.2006.10.006[published Online First: Epub Date]|.
28. Tormo MJ, Navarro C, Chirlaque M-D, et al. Physical sports activity during leisure time and dietary intake of foods and nutrients in a large Spanish cohort. Int J Sport Nutr Exerc Metab 2003;**13**:47-64
29. Parsons TJ, Power C, Manor O. Longitudinal physical activity and diet patterns in the 1958 British Birth Cohort. Med Sci Sports Exerc 2006;**38**(3):547-54 doi: 10.1249/01.mss.0000188446.65651.67[published Online First: Epub Date]|.
30. de Nazelle A, Nieuwenhuijsen MJ, Anto JM, et al. Improving health through policies that promote active travel: a review of evidence to support integrated health impact assessment. Environ Int 2011;**37**(4):766-77 doi: 10.1016/j.envint.2011.02.003[published Online First: Epub Date]|.
31. McAloney K, Graham H, Law C, et al. A scoping review of statistical approaches to the analysis of multiple health-related behaviours. Prev Med 2013;**56**(6):365-71 doi: 10.1016/j.ypmed.2013.03.002[published Online First: Epub Date]|.
32. Spring B, Moller AC, Coons MJ. Multiple health behaviours: overview and implications. J Public Health (Oxf) 2012;**34 Suppl 1**:i3-10 doi: 10.1093/pubmed/fdr111[published Online First: Epub Date]|.
33. Truelove HB, Carrico AR, Weber EU, et al. Positive and negative spillover of pro-environmental behavior: An integrative review and theoretical framework. Global Environmental Change 2014;**29**:127-38 doi: 10.1016/j.gloenvcha.2014.09.004[published Online First: Epub Date]|.
34. Dolan P, Galizzi MM. Like ripples on a pond: Behavioral spillovers and their implications for research and policy. J Econ Psychol 2015;**47**:1-16 doi: 10.1016/j.joep.2014.12.003[published Online First: Epub Date]|.
35. Spring B, Schneider K, McFadden HG, et al. Multiple behavior changes in diet and activity: a randomized controlled trial using mobile technology. Arch Intern Med 2012;**172**(10):789-96 doi: 10.1001/archinternmed.2012.1044[published Online First: Epub Date]|.

For peer review only - http://bmjopen.bmj.com/site/about/guidelines.xhtml

36. Sudlow C, Gallacher J, Allen N, et al. UK biobank: an open access resource for identifying the causes of a wide range of complex diseases of middle and old age. PLoS Med 2015;**12**(3):e1001779 doi: 10.1371/journal.pmed.1001779[published Online First: Epub Date]|.
37. Flint E, Cummins S. Active commuting and obesity in mid-life: cross-sectional, observational evidence from UK Biobank. The Lancet Diabetes & Endocrinology 2016;**4**(5):420-35 doi: 10.1016/s2213-8587(16)00053-x[published Online First: Epub Date]|.
38. NHS. 5 A Day portion sizes. Secondary 5 A Day portion sizes 2018. https://http://www.nhs.uk/live-well/eat-well/5-a-day-portion-sizes/.
39. Bradbury KE, Tong TYN, Key TJ. Dietary Intake of High-Protein Foods and Other Major Foods in Meat-Eaters, Poultry-Eaters, Fish-Eaters, Vegetarians, and Vegans in UK Biobank. Nutrients 2017;**9**(12):1317
40. NHS. Physical activity guidelines for adults. Secondary Physical activity guidelines for adults 2018. https://http://www.nhs.uk/live-well/exercise//.
41. Galante J, Adamska L, Young A, et al. The acceptability of repeat Internet-based hybrid diet assessment of previous 24-h dietary intake: administration of the Oxford WebQ in UK Biobank. The British journal of nutrition 2016;**115**(4):681-6 doi: 10.1017/s0007114515004821[published Online First: Epub Date]|.
42. Stata Statistical Software: Release 14 [program]. College Station, TX: StataCorp LP, 2015.
43. Joseph RJ, Alonso-Alonso M, Bond DS, et al. The neurocognitive connection between physical activity and eating behaviour. Obes Rev 2011;**12**(10):800-12 doi: 10.1111/j.1467-789X.2011.00893.x[published Online First: Epub Date]|.
44. Loprinzi PD. Physical activity is the best buy in medicine, but perhaps for less obvious reasons. Prev Med 2015;**75**:23-24 doi: 10.1016/j.ypmed.2015.01.033[published Online First: Epub Date]|.
45. Williams R. Understanding and interpreting generalized ordered logit models. The Journal of Mathematical Sociology 2016;**40**(1):7-20
46. oparallel: Stata module providing post-estimation command for testing the parallel regression assumption [program], 2013.
47. Williams R. Generalized ordered logit/partial proportional odds models for ordinal dependent variables. Stata Journal 2006;**6**(1):58
48. Bates B, Lennox A, Prentice A, et al. National Diet and Nutrition Survey: Results from Years 1–4 (combined) of the Rolling Programme (2008/2009–2011/12). Public Health England, and Food Standards Agency: London 2014
49. DfT. National Travel Survey: England 2016. London: Department for Transport, 2017.
50. Fry A, Littlejohns TJ, Sudlow C, et al. Comparison of Sociodemographic and Health-Related Characteristics of UK Biobank Participants with the General Population. Am J Epidemiol 2017 doi: 10.1093/aje/kwx246[published Online First: Epub Date]|.
51. Juliano L. The Grocer: Plant based food, Research on behalf of The Grocer – April 2018: Harris Interactive, 2018.
52. Smith MA, Böhnke JR, Graham H, et al. OP24 Associations between active travel and diet: An exploration of pro-health, low carbon behaviours in the National Diet and Nutrition Survey: BMJ Publishing Group Ltd, 2016.
53. Smith M. Prevalence, patterning, and predictors of health-and climate-relevant lifestyles in the UK: A cross-sectional study of travel and dietary behaviour in two national datasets. University of York, 2018.
54. Bradbury KE, Young HJ, Guo W, et al. Dietary assessment in UK Biobank: an evaluation of the performance of the touchscreen dietary questionnaire. Journal of nutritional science 2018;**7**:e6 doi: 10.1017/jns.2017.66[published Online First: Epub Date]|.
55. Gillman MW, Pinto BM, Tennstedt S, et al. Relationships of physical activity with dietary behaviors among adults. Prev Med 2001;**32**(3):295-301 doi: 10.1006/pmed.2000.0812[published Online First: Epub Date]|.

For peer review only - http://bmjopen.bmj.com/site/about/guidelines.xhtml

56. Martin A, Panter J, Suhrcke M, et al. Impact of changes in mode of travel to work on changes in body mass index: evidence from the British Household Panel Survey. J Epidemiol Community Health 2015;**69**(8):753-61 doi: 10.1136/jech-2014-205211[published Online First: Epub Date]|.
57. Flint E, Cummins S, Sacker A. Associations between active commuting, body fat, and body mass index: population based, cross sectional study in the United Kingdom. BMJ 2014;**349** doi: 10.1136/bmj.g4887[published Online First: Epub Date]|.
58. Celis-Morales CA, Lyall DM, Welsh P, et al. Association between active commuting and incident cardiovascular disease, cancer, and mortality: prospective cohort study. BMJ 2017;**357**:j1456 doi: 10.1136/bmj.j1456[published Online First: Epub Date]|.
59. Panter J, Mytton O, Sharp S, et al. Using alternatives to the car and risk of all-cause, cardiovascular and cancer mortality. Heart 2018;**104**(21):1749-55 doi: 10.1136/heartjnl-2017-312699[published Online First: Epub Date]|.
60. Flay B, Petraitis J. The theory of triadic influence. Adv Med Sociol 1994;**4**:19-44
61. DfT. Walking and Cycling Statistics, England: 2017. London Department for Transport, 2018.
62. IPCC. *Global Warming of 1.5° C: An IPCC Special Report on the Impacts of Global Warming of 1.5° C Above Pre-industrial Levels and Related Global Greenhouse Gas Emission Pathways, in the Context of Strengthening the Global Response to the Threat of Climate Change, Sustainable Development, and Efforts to Eradicate Poverty*: Intergovernmental Panel on Climate Change, 2018.
63. Akenji L, Lettenmeier M, Koide R, et al. 1.5-Degree Lifestyles: Targets and options for reducing lifestyle carbon footprints: Institute for Global Environmental Strategies, Aalto University, and D-mat ltd. (Hayama, Japan) 2019.

For peer review only

For peer review only - http://bmjopen.bmj.com/site/about/guidelines.xhtml

Figure S1 – Putative relationships between active travel, dietary consumption, physical activity, energy intake and other covariates

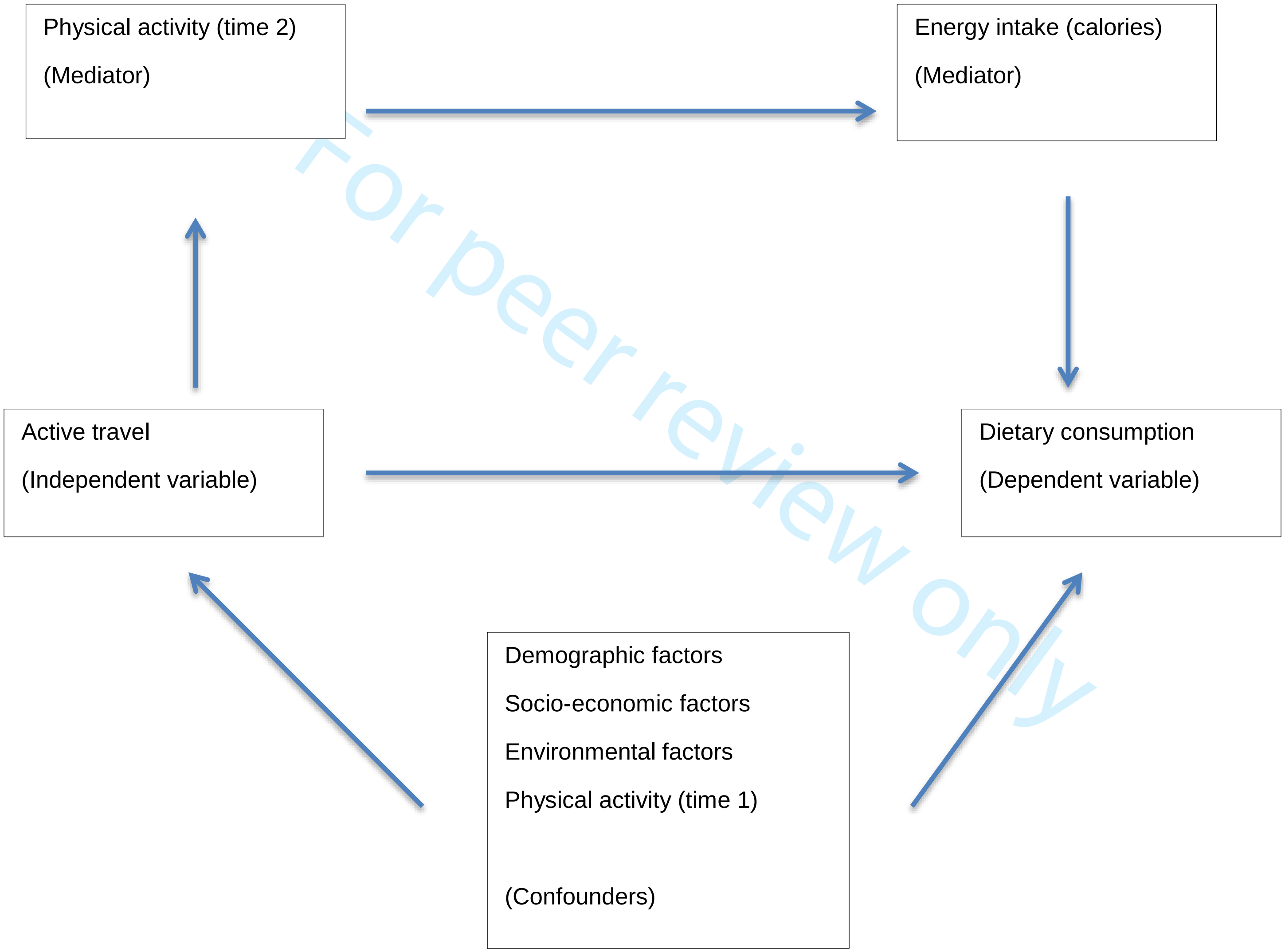

For peer review only

Figure S1 provides an overview of the putative relationships between active travel, dietary consumption, physical activity, energy intake and other covariates in this study.

People who are more physically active tend to have healthier diets [1] and may be more likely to engage in active travel [2] – this means that one's initial physical activity level (time 1) may be a confounder of the relationship between active travel and dietary consumption, in the same way that demographic, socio-economic, and environmental factors may also act as confounders.

At the same time, we also know that those who engage in active travel may accumulate additional physical activity [3], which may ultimately lead them to consume more food and have a higher energy intake (time 2). In this way, it is possible for physical activity and energy intake to act as mediators of the relationship between active travel and dietary consumption.

It is not possible to tease out these distinctions with cross-sectional data, where all of these variables have been measured at the same point in time. To account for this limitation, we have presented three models with different levels of covariate adjustment: Model 1 is unadjusted, Model 2 is adjusted for demographic, socio-economic and environmental factors, and Model 3 (sensitivity analysis) is adjusted for physical activity level and energy intake.

References

1. Noble N, Paul C, Turon H, et al. Which modifiable health risk behaviours are related? A systematic review of the clustering of Smoking, Nutrition, Alcohol and Physical activity ('SNAP') health risk factors. Prev Med 2015;**81**:16-41 doi: 10.1016/j.ypmed.2015.07.003[published Online First: Epub Date]|.

2. Hutchinson J, Prady SL, Smith MA, et al. A Scoping Review of Observational Studies Examining Relationships between Environmental Behaviors and Health Behaviors. Int J Environ Res Public Health 2015;**12**(5):4833-58

3. Sahlqvist S, Goodman A, Cooper AR, et al. Change in active travel and changes in recreational and total physical activity in adults: longitudinal findings from the iConnect study. International Journal of Behavioral Nutrition and Physical Activity 2013;**10**(1):28 doi: 10.1186/1479-5868-10-28[published Online First: Epub Date]|.

For peer review only

Table S1 – Results of ordinal logistic models between any active travel and fruit and vegetable (FV) consumption among females in UKB (n=217,168)

VARIABLES	Model 1	Model 2
Any active travel (ref: None)	1.42***<br>(1.40 - 1.44)	1.43***<br>(1.40 - 1.45)
Age at baseline		1.04***<br>(1.04 - 1.05)
Ethnic group (ref: White British)		
Other white		1.38***<br>(1.33 - 1.42)
South Asian		2.19***<br>(2.04 - 2.37)
Black		1.63***<br>(1.52 - 1.74)
Chinese		1.67***<br>(1.45 - 1.93)
Mixed		1.16**<br>(1.05 - 1.28)
Other		2.29***<br>(2.08 - 2.53)
Highest qualification (ref: Degree level)		
A levels/AS levels or equivalent		0.86***<br>(0.83 - 0.88)
O levels/GCSEs or equivalent		0.77***<br>(0.75 - 0.79)
CSEs or equivalent		0.72***<br>(0.70 - 0.75)
NVQ or HND or HNC or equivalent		0.79***<br>(0.76 - 0.82)
Other professional qualifications		0.89***<br>(0.86 - 0.92)
No qualifications		0.63***<br>(0.62 - 0.65)
Occupation class (ref: Higher man / prof)		
Lower managerial / professional		1.08***<br>(1.05 - 1.11)
Intermediate occupations		1.02<br>(0.99 - 1.06)
Small employers & own accounts		1.11***<br>(1.04 - 1.17)
Lower supervisory & technical		1.16*<br>(1.03 - 1.31)
Semi-routine occupations		1.03<br>(0.99 - 1.07)
Routine occupations		0.98<br>(0.93 - 1.04)
Not classified		1.03<br>(1.00 - 1.06)
Household income (ref: £<18 000)		
£18,000 to 30,999		1.10***<br>(1.07 - 1.13)
£31,000 to 51,999		1.21***<br>(1.17 - 1.24)
£52,000 to 100,000		1.29***<br>(1.25 - 1.33)
£Greater than 100,000		1.32***<br>(1.26 - 1.38)

For peer review only

Household size (ref: One)		
2		0.95***
		(0.93 - 0.97)
3		0.88***
		(0.86 - 0.91)
4		0.85***
		(0.82 - 0.87)
5+		0.87***
		(0.83 - 0.91)
Region (ref: London)		
North East England		1.07***
		(1.03 - 1.11)
Yorkshire and the Humber		0.99
		(0.96 - 1.02)
West Midlands		1.06***
		(1.03 - 1.10)
East Midlands		1.08***
		(1.04 - 1.12)
South East England		1.12***
		(1.08 - 1.16)
South West England		1.14***
		(1.10 - 1.18)
North West England		0.99
		(0.96 - 1.02)
Wales		1.12***
		(1.07 - 1.17)
Scotland		0.95**
		(0.91 - 0.98)
Townsend deprivation		0.98***
		(0.98 - 0.99)
Urban (ref: Rural)		0.94***
		(0.92 - 0.96)
Cars per household (ref: None)		
One		1.12***
		(1.08 - 1.16)
Two		1.10***
		(1.06 - 1.14)
Three		1.07**
		(1.02 - 1.12)
Four or more		1.09**
		(1.03 - 1.17)
Observations	217,168	217,168

*** p<0.001, ** p<0.01, * p<0.05

For peer review only

Table S2 – Results of ordinal logistic models between any active travel and fruit and vegetable (FV) consumption among males in UKB (n=195,131)

VARIABLES	Model 1	Model 2
Any active travel (ref: None)	1.37***	1.35***
	(1.34 - 1.39)	(1.33 - 1.37)
Age at baseline		1.03***
		(1.03 - 1.03)
Ethnic group (ref: White British)		
Other white		1.32***
		(1.27 - 1.36)
South Asian		2.24***
		(2.10 - 2.38)
Black		1.50***
		(1.39 - 1.63)
Chinese		1.68***
		(1.41 - 2.00)
Mixed		1.11
		(0.98 - 1.26)
Other		2.23***
		(2.02 - 2.47)
Highest qualification (ref: Degree level)		
A levels/AS levels or equivalent		0.79***
		(0.76 - 0.81)
O levels/GCSEs or equivalent		0.75***
		(0.73 - 0.77)
CSEs or equivalent		0.75***
		(0.72 - 0.78)
NVQ or HND or HNC or equivalent		0.83***
		(0.80 - 0.85)
Other professional qualifications		0.82***
		(0.79 - 0.86)
No qualifications		0.75***
		(0.73 - 0.77)
Occupation class (ref: Higher man / prof)		
Lower managerial / professional		1.03*
		(1.00 - 1.05)
Intermediate occupations		1.05**
		(1.01 - 1.09)
Small employers & own accounts		1.04
		(1.00 - 1.09)
Lower supervisory & technical		1.08***
		(1.03 - 1.12)
Semi-routine occupations		1.00
		(0.96 - 1.04)
Routine occupations		1.04
		(0.99 - 1.08)
Not classified		0.99
		(0.96 - 1.02)
Household income (ref: £<18 000)		
£18,000 to 30,999		1.11***
		(1.08 - 1.14)
£31,000 to 51,999		1.18***
		(1.15 - 1.22)
£52,000 to 100,000		1.26***
		(1.22 - 1.31)
£Greater than 100,000		1.33***
		(1.27 - 1.39)

For peer review only

Household size (ref: One)			
	2		1.12***
			(1.09 - 1.15)
	3		1.04*
			(1.01 - 1.08)
	4		1.03
			(1.00 - 1.07)
	5+		1.01
			(0.96 - 1.05)
Region (ref: London)			
	North East England		1.02
			(0.98 - 1.05)
	Yorkshire and the Humber		0.97
			(0.94 - 1.01)
	West Midlands		1.00
			(0.96 - 1.04)
	East Midlands		1.03
			(0.99 - 1.07)
	South East England		1.00
			(0.96 - 1.04)
	South West England		1.02
			(0.98 - 1.06)
	North West England		0.94***
			(0.91 - 0.97)
	Wales		1.05*
			(1.00 - 1.10)
	Scotland		0.83***
			(0.80 - 0.86)
Townsend deprivation			0.99***
			(0.98 - 0.99)
Urban (ref: Rural)			0.96**
			(0.94 - 0.98)
Cars per household (ref: None)			
	One		1.11***
			(1.07 - 1.15)
	Two		1.02
			(0.98 - 1.06)
	Three		0.94**
			(0.89 - 0.98)
	Four or more		0.92**
			(0.86 - 0.98)
Observations		195,131	195,131

*** p<0.001, ** p<0.01, * p<0.05

For peer review only

Table S3 – Results of ordinal logistic models between any active travel and red and processed meat (RPM) consumption among females in UKB (n=217,168)

VARIABLES	Model 1	Model 2
Any active travel (ref: None)	0.85***	0.88***
	(0.84 - 0.87)	(0.87 - 0.90)
Age at baseline		1.01***
		(1.01 - 1.01)
Ethnic group (ref: White British)		
Other white		0.99
		(0.95 - 1.02)
South Asian		0.27***
		(0.25 - 0.29)
Black		1.06
		(0.99 - 1.14)
Chinese		2.12***
		(1.83 - 2.45)
Mixed		0.97
		(0.88 - 1.08)
Other		0.91*
		(0.82 - 1.00)
Highest qualification (ref: Degree level)		
A levels/AS levels or equivalent		1.20***
		(1.17 - 1.24)
O levels/GCSEs or equivalent		1.27***
		(1.24 - 1.30)
CSEs or equivalent		1.30***
		(1.24 - 1.35)
NVQ or HND or HNC or equivalent		1.22***
		(1.17 - 1.28)
Other professional qualifications		1.16***
		(1.12 - 1.21)
No qualifications		1.35***
		(1.31 - 1.39)
Occupation class (ref: Higher man / prof)		
Lower managerial / professional		0.96**
		(0.93 - 0.99)
Intermediate occupations		1.06**
		(1.02 - 1.09)
Small employers & own accounts		1.00
		(0.94 - 1.07)
Lower supervisory & technical		1.07
		(0.94 - 1.21)
Semi-routine occupations		1.08***
		(1.04 - 1.13)
Routine occupations		1.20***
		(1.13 - 1.27)
Not classified		1.21***
		(1.17 - 1.25)
Household income (ref: £<18 000)		
£18,000 to 30,999		1.00
		(0.98 - 1.03)
£31,000 to 51,999		0.94***
		(0.92 - 0.97)
£52,000 to 100,000		0.93***
		(0.90 - 0.96)
£Greater than 100,000		0.93**
		(0.88 - 0.97)

For peer review only

Household size (ref: One)		
2		1.45***
		(1.41 - 1.48)
3		1.57***
		(1.52 - 1.63)
4		1.79***
		(1.73 - 1.86)
5+		1.92***
		(1.83 - 2.01)
Region (ref: London)		
North East England		0.97
		(0.94 - 1.01)
Yorkshire and the Humber		1.01
		(0.98 - 1.05)
West Midlands		0.97
		(0.93 - 1.00)
East Midlands		1.00
		(0.96 - 1.04)
South East England		1.03
		(0.99 - 1.07)
South West England		0.94***
		(0.90 - 0.97)
North West England		1.15***
		(1.11 - 1.19)
Wales		0.89***
		(0.85 - 0.94)
Scotland		1.20***
		(1.15 - 1.24)
Townsend deprivation		1.00
		(1.00 - 1.00)
Urban (ref: Rural)		0.96**
		(0.94 - 0.98)
Cars per household (ref: None)		
One		1.04*
		(1.01 - 1.08)
Two		1.17***
		(1.12 - 1.21)
Three		1.24***
		(1.18 - 1.30)
Four or more		1.29***
		(1.20 - 1.38)
Observations	217,168	217,168

*** p<0.001, ** p<0.01, * p<0.05

For peer review only

Table S4 – Results of ordinal logistic models between any active travel and red and processed meat (RPM) consumption among males in UKB (n=195,131)

VARIABLES	Model 1	Model 2
Any active travel (ref: None)	0.87*** (0.85 - 0.88)	0.89*** (0.87 - 0.91)
Age at baseline		1.00 (1.00 - 1.00)
Ethnic group (ref: White British)		
Other white		1.01 (0.97 - 1.05)
South Asian		0.26*** (0.25 - 0.28)
Black		0.82*** (0.76 - 0.90)
Chinese		1.33** (1.09 - 1.61)
Mixed		1.08 (0.94 - 1.23)
Other		0.74*** (0.66 - 0.82)
Highest qualification (ref: Degree level)		
A levels/AS levels or equivalent		1.20*** (1.16 - 1.23)
O levels/GCSEs or equivalent		1.22*** (1.19 - 1.26)
CSEs or equivalent		1.22*** (1.17 - 1.27)
NVQ or HND or HNC or equivalent		1.20*** (1.16 - 1.24)
Other professional qualifications		1.09*** (1.04 - 1.14)
No qualifications		1.16*** (1.13 - 1.20)
Occupation class (ref: Higher man / prof)		
Lower managerial / professional		0.95*** (0.92 - 0.98)
Intermediate occupations		1.01 (0.97 - 1.05)
Small employers & own accounts		1.19*** (1.14 - 1.25)
Lower supervisory & technical		1.18*** (1.13 - 1.23)
Semi-routine occupations		1.20*** (1.15 - 1.26)
Routine occupations		1.28*** (1.22 - 1.34)
Not classified		1.14*** (1.11 - 1.18)
Household income (ref: £<18 000)		
£18,000 to 30,999		0.96* (0.94 - 0.99)
£31,000 to 51,999		0.97 (0.94 - 1.01)
£52,000 to 100,000		0.91*** (0.88 - 0.95)
£Greater than 100,000		0.89*** (0.85 - 0.94)

For peer review only

Household size (ref: One)			
	2		1.06***
			(1.03 - 1.09)
	3		1.17***
			(1.13 - 1.21)
	4		1.25***
			(1.20 - 1.29)
	5+		1.35***
			(1.29 - 1.42)
Region (ref: London)			
	North East England		0.99
			(0.95 - 1.03)
	Yorkshire and the Humber		1.02
			(0.98 - 1.06)
	West Midlands		1.02
			(0.98 - 1.07)
	East Midlands		0.97
			(0.93 - 1.01)
	South East England		1.03
			(0.99 - 1.07)
	South West England		0.96*
			(0.92 - 1.00)
	North West England		1.15***
			(1.11 - 1.19)
	Wales		0.91***
			(0.87 - 0.96)
	Scotland		1.16***
			(1.11 - 1.21)
Townsend deprivation			1.01***
			(1.01 - 1.01)
Urban (ref: Rural)			0.99
			(0.97 - 1.02)
Cars per household (ref: None)			
	One		0.98
			(0.94 - 1.02)
	Two		1.08***
			(1.04 - 1.13)
	Three		1.23***
			(1.17 - 1.30)
	Four or more		1.30***
			(1.21 - 1.39)
Observations		195,131	195,131

*** p<0.001, ** p<0.01, * p<0.05

For peer review only

Figure S2 – Associations between travel and FV consumption (Model 2, Table 4)

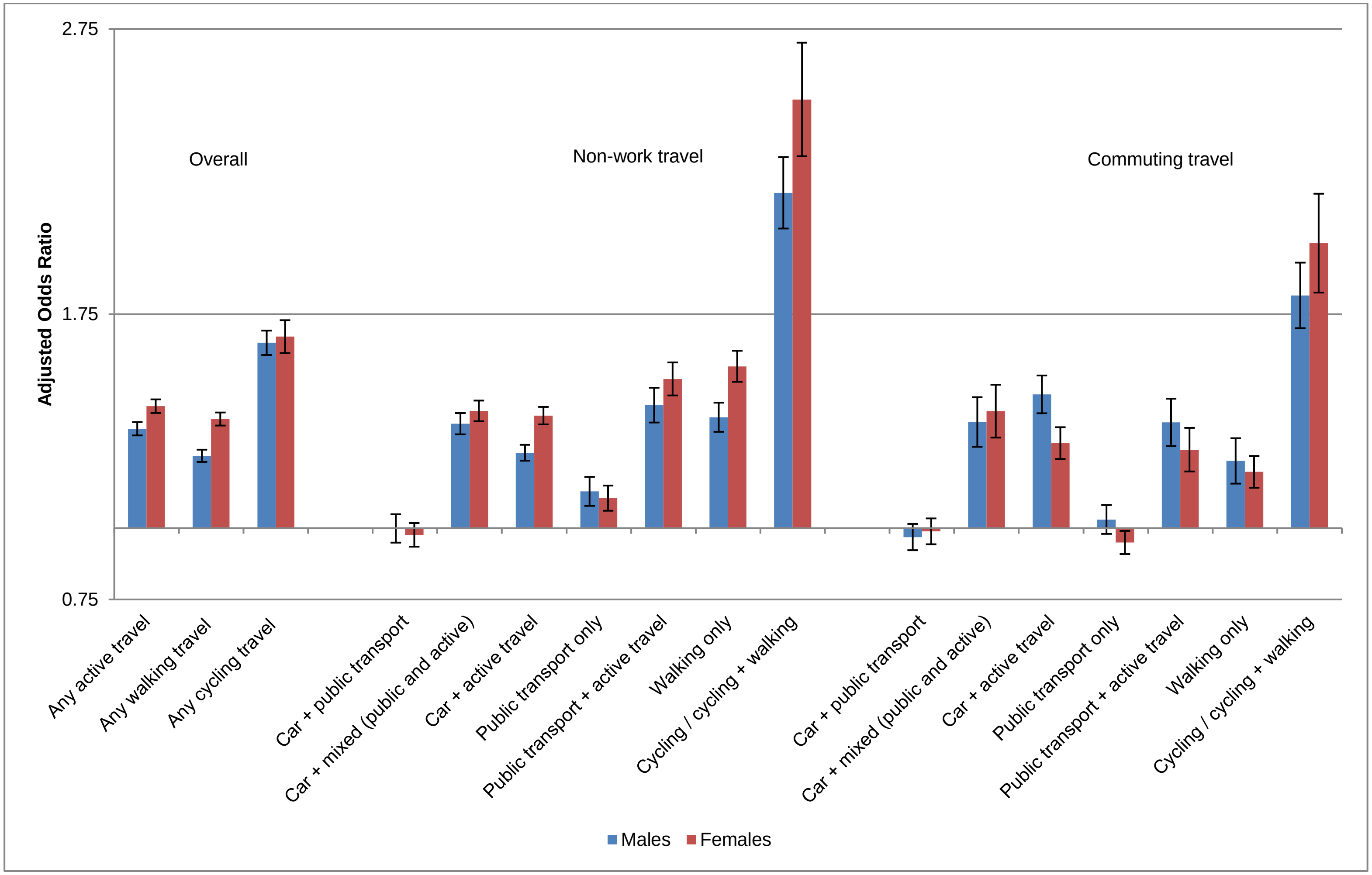

Whiskers = 95% confidence interval

Figure S3 – Associations between travel and RPM consumption (Model 2, Table 5)

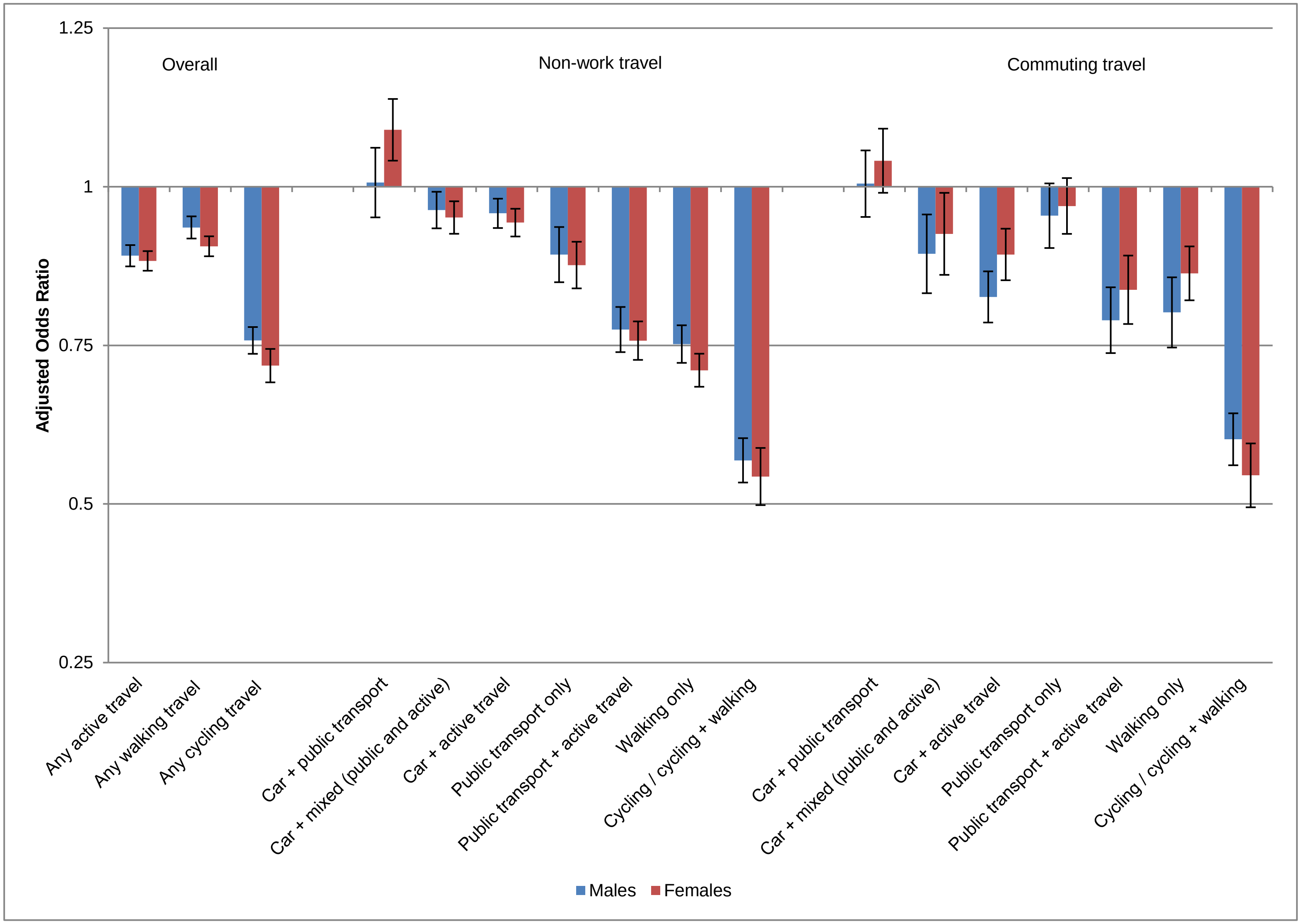

Whiskers = 95% confidence interval

Table S5 – Results of generalized ordered logit models between measures of active travel and FV consumption, stratified by gender in UKB (n=412,299)

TRAVEL VARIABLES	Males (n=195,131)		Females (n=217,168)	
	Model 2 [a]		Model 2 [a]	
	1 v. 2 + 3 [b]	1 + 2 v. 3	1 v. 2 + 3 [b]	1 + 2 v. 3
Any active travel (ref: No)	1.37*** (1.35 - 1.40)	1.32*** (1.30 - 1.35)	1.53*** (1.49 - 1.56)	1.38*** (1.35 - 1.40)
Any walking (ref: No)	1.28*** (1.26 - 1.31)	1.23*** (1.20 - 1.25)	1.47*** (1.44 - 1.51)	1.34*** (1.32 - 1.36)
Any cycling (ref: No)	1.71*** (1.66 - 1.76)	1.61*** (1.57 - 1.66)	1.93*** (1.84 - 2.04)	1.60*** (1.55 - 1.66)
Non-work travel [b] (ref: Car only)				
Car + public transport	1.00 (0.95 - 1.05)	1.00 (0.95 - 1.05)	0.98 (0.94 - 1.02)	0.98 (0.94 - 1.02)
Car + mixed (public and active)	1.42*** (1.38 - 1.47)	1.32*** (1.28 - 1.36)	1.55*** (1.50 - 1.61)	1.36*** (1.32 - 1.39)
Car + active travel	1.29*** (1.26 - 1.32)	1.24*** (1.21 - 1.27)	1.49*** (1.45 - 1.54)	1.35*** (1.32 - 1.38)
Public transport only	1.14*** (1.09 - 1.19)	1.14*** (1.09 - 1.19)	1.11*** (1.07 - 1.16)	1.11*** (1.07 - 1.16)
Public transport + active travel	1.47*** (1.40 - 1.54)	1.39*** (1.33 - 1.46)	1.66*** (1.58 - 1.74)	1.45*** (1.40 - 1.51)
Walking only	1.39*** (1.34 - 1.44)	1.39*** (1.34 - 1.44)	1.62*** (1.55 - 1.69)	1.53*** (1.48 - 1.59)
Cycling / cycling + walking	2.27*** (2.11 - 2.44)	2.10*** (1.97 - 2.23)	2.84*** (2.50 - 3.21)	2.39*** (2.21 - 2.60)
Commuting travel [c] (ref: Car only)				
Car + public transport	0.97 (0.92 - 1.02)	0.97 (0.92 - 1.02)	0.99 (0.95 - 1.03)	0.99 (0.95 - 1.03)
Car + mixed (public and active)	1.38*** (1.29 - 1.47)	1.38*** (1.29 - 1.47)	1.53*** (1.39 - 1.67)	1.37*** (1.28 - 1.47)
Car + active travel	1.47*** (1.41 - 1.54)	1.47*** (1.41 - 1.54)	1.35*** (1.28 - 1.43)	1.28*** (1.22 - 1.33)
Public transport only	1.03 (0.99 - 1.09)	1.03 (0.99 - 1.09)	0.95* (0.91 - 0.99)	0.95* (0.91 - 0.99)
Public transport + active travel	1.37*** (1.29 - 1.46)	1.37*** (1.29 - 1.46)	1.27*** (1.20 - 1.35)	1.27*** (1.20 - 1.35)
Walking only	1.19*** (1.11 - 1.28)	1.29*** (1.20 - 1.38)	1.20*** (1.14 - 1.25)	1.20*** (1.14 - 1.25)
Cycling / cycling + walking	1.82*** (1.71 - 1.94)	1.82*** (1.71 - 1.94)	2.00*** (1.84 - 2.18)	2.00*** (1.84 - 2.18)

*** p<0.001, ** p<0.01, * p<0.05

a) Adjusted for: age, ethnic group, education, occupational class, household income, household size, number of cars, assessment centre location, population density, Townsend score

b) Shading and boxes indicate variables with different relationships across the levels of the outcome variable: 1 = <3 portions FV, 2 = 3-<5 portions FV, 3 = 5+ portions FV

For peer review only

Table S6 – Results of generalized ordered logit models between measures of active travel and RPM consumption, stratified by gender in UKB (n=412,299)

TRAVEL VARIABLES	Males (n=195,131)		Females (n=217,168)	
	Model 2 [a]		Model 2 [a]	
	1 v. 2 + 3 [b]	1 + 2 v. 3	1 v. 2 + 3 [b]	1 + 2 v. 3
Any active travel (ref: No)	0.72*** (0.68 - 0.76)	0.90*** (0.89 - 0.92)	0.79*** (0.76 - 0.81)	0.90*** (0.89 - 0.92)
Any walking (ref: No)	0.86*** (0.81 - 0.90)	0.94*** (0.92 - 0.96)	0.83*** (0.80 - 0.86)	0.92*** (0.91 - 0.94)
Any cycling (ref: No)	0.56*** (0.52 - 0.59)	0.78*** (0.76 - 0.81)	0.63*** (0.60 - 0.67)	0.77*** (0.75 - 0.80)
Non-work travel [b] (ref: Car only)				
Car + public transport	1.01 (0.95 - 1.06)	1.01 (0.95 - 1.06)	1.10*** (1.05 - 1.15)	1.10*** (1.05 - 1.15)
Car + mixed (public and active)	0.78*** (0.72 - 0.84)	0.97 (0.94 - 1.00)	0.87*** (0.83 - 0.92)	0.97* (0.94 - 0.99)
Car + active travel	0.83*** (0.77 - 0.89)	0.96** (0.94 - 0.99)	0.86*** (0.82 - 0.90)	0.96*** (0.93 - 0.98)
Public transport only	0.75*** (0.67 - 0.84)	0.89*** (0.85 - 0.94)	0.87*** (0.83 - 0.90)	0.87*** (0.83 - 0.90)
Public transport + active travel	0.60*** (0.54 - 0.66)	0.79*** (0.75 - 0.82)	0.67*** (0.62 - 0.71)	0.79*** (0.75 - 0.82)
Walking only	0.62*** (0.56 - 0.68)	0.76*** (0.73 - 0.79)	0.64*** (0.60 - 0.68)	0.73*** (0.70 - 0.76)
Cycling / cycling + walking	0.37*** (0.33 - 0.41)	0.61*** (0.58 - 0.65)	0.49*** (0.44 - 0.55)	0.60*** (0.55 - 0.66)
Commuting travel [c] (ref: Car only)				
Car + public transport	1.00 (0.95 - 1.05)	1.00 (0.95 - 1.05)	0.95 (0.87 - 1.04)	1.06* (1.01 - 1.11)
Car + mixed (public and active)	0.58*** (0.51 - 0.67)	0.93* (0.87 - 0.99)	0.77*** (0.69 - 0.85)	0.98 (0.91 - 1.06)
Car + active travel	0.65*** (0.58 - 0.73)	0.84*** (0.80 - 0.88)	0.81*** (0.75 - 0.87)	0.92*** (0.88 - 0.96)
Public transport only	0.82*** (0.73 - 0.92)	0.96 (0.91 - 1.01)	0.96 (0.92 - 1.01)	0.96 (0.92 - 1.01)
Public transport + active travel	0.57*** (0.50 - 0.65)	0.82*** (0.77 - 0.88)	0.73*** (0.66 - 0.80)	0.89*** (0.83 - 0.95)
Walking only	0.65*** (0.56 - 0.75)	0.82*** (0.76 - 0.87)	0.79*** (0.73 - 0.86)	0.88*** (0.84 - 0.93)
Cycling / cycling + walking	0.39*** (0.35 - 0.44)	0.65*** (0.61 - 0.70)	0.46*** (0.41 - 0.52)	0.65*** (0.59 - 0.71)

*** p<0.001, ** p<0.01, * p<0.05

a) Adjusted for: age, ethnic group, education, occupational class, household income, household size, number of cars, assessment centre location, population density, Townsend score

b) Shading and boxes indicate variables with different relationships across the levels of the outcome variable: 1 = 0 g RPM per day; 2 = >0-70 g RPM per day; 3 = >70 g RPM per day

For peer review only

Table S7 – Sensitivity analysis: results of ordinal logistic models between any active travel and FV consumption among females in UKB (n=95,475)

VARIABLES	Model 1	Model 2	Model 3
Any active travel (ref: None)	1.42***	1.42***	1.35***
	(1.38 - 1.45)	(1.38 - 1.45)	(1.32 - 1.39)
Age at baseline		1.05***	1.05***
		(1.04 - 1.05)	(1.04 - 1.05)
Ethnic group (ref: White British)			
Other white		1.39***	1.38***
		(1.32 - 1.46)	(1.31 - 1.45)
South Asian		2.05***	2.09***
		(1.81 - 2.33)	(1.84 - 2.37)
Black		1.60***	1.58***
		(1.43 - 1.80)	(1.41 - 1.76)
Chinese		1.54***	1.55***
		(1.23 - 1.94)	(1.23 - 1.95)
Mixed		1.06	1.05
		(0.91 - 1.22)	(0.90 - 1.21)
Other		2.13***	2.16***
		(1.82 - 2.48)	(1.85 - 2.53)
Highest qualification (ref: Degree level)			
A levels/AS levels or equivalent		0.87***	0.87***
		(0.84 - 0.90)	(0.84 - 0.91)
O levels/GCSEs or equivalent		0.78***	0.79***
		(0.75 - 0.81)	(0.76 - 0.81)
CSEs or equivalent		0.71***	0.71***
		(0.66 - 0.75)	(0.67 - 0.76)
NVQ or HND or HNC or equivalent		0.82***	0.82***
		(0.77 - 0.88)	(0.76 - 0.88)
Other professional qualifications		0.92**	0.91**
		(0.87 - 0.97)	(0.86 - 0.96)
No qualifications		0.68***	0.68***
		(0.64 - 0.71)	(0.64 - 0.72)
Occupation class (ref: Higher man / prof)			
Lower managerial / professional		1.07***	1.05**
		(1.03 - 1.11)	(1.01 - 1.10)
Intermediate occupations		1.01	1.00
		(0.97 - 1.06)	(0.96 - 1.05)
Small employers & own accounts		1.12**	1.08
		(1.04 - 1.22)	(0.99 - 1.17)
Lower supervisory & technical		1.14	1.04
		(0.96 - 1.37)	(0.87 - 1.25)
Semi-routine occupations		1.10**	1.06
		(1.04 - 1.16)	(1.00 - 1.12)
Routine occupations		0.96	0.89*
		(0.86 - 1.07)	(0.80 - 0.99)
Not classified		1.04	0.99
		(0.99 - 1.09)	(0.95 - 1.04)
Household income (ref: £<18 000)			
£18,000 to 30,999		1.11***	1.11***
		(1.06 - 1.15)	(1.07 - 1.16)
£31,000 to 51,999		1.23***	1.25***
		(1.18 - 1.28)	(1.19 - 1.30)
£52,000 to 100,000		1.31***	1.32***
		(1.25 - 1.37)	(1.26 - 1.39)
£Greater than 100,000		1.34***	1.33***
		(1.25 - 1.43)	(1.24 - 1.42)

For peer review only

Household size (ref: One)			
2		0.94***	0.93***
		(0.90 - 0.97)	(0.90 - 0.97)
3		0.90***	0.90***
		(0.86 - 0.94)	(0.86 - 0.94)
4		0.84***	0.84***
		(0.80 - 0.89)	(0.80 - 0.88)
5+		0.84***	0.82***
		(0.79 - 0.90)	(0.77 - 0.88)
Region (ref: London)			
North East England		1.11***	1.14***
		(1.05 - 1.16)	(1.08 - 1.20)
Yorkshire and the Humber		1.06**	1.07**
		(1.01 - 1.11)	(1.02 - 1.12)
West Midlands		1.05	1.07**
		(1.00 - 1.11)	(1.02 - 1.13)
East Midlands		1.15***	1.17***
		(1.09 - 1.22)	(1.11 - 1.25)
South East England		1.18***	1.21***
		(1.12 - 1.24)	(1.15 - 1.27)
South West England		1.19***	1.21***
		(1.14 - 1.26)	(1.16 - 1.28)
North West England		1.07**	1.09***
		(1.02 - 1.12)	(1.04 - 1.14)
Wales		1.16***	1.20***
		(1.08 - 1.26)	(1.11 - 1.29)
Scotland		1.09**	1.12***
		(1.03 - 1.16)	(1.05 - 1.19)
Townsend deprivation		0.99***	0.99***
		(0.99 - 0.99)	(0.99 - 1.00)
Urban (ref: Rural)		0.93***	0.95**
		(0.90 - 0.97)	(0.91 - 0.98)
Cars per household (ref: None)			
One		1.00	1.01
		(0.95 - 1.06)	(0.95 - 1.06)
Two		0.96	0.95
		(0.90 - 1.02)	(0.90 - 1.01)
Three		0.92*	0.91*
		(0.86 - 0.99)	(0.85 - 0.98)
Four or more		0.96	0.95
		(0.87 - 1.06)	(0.86 - 1.05)
Meets physical activity guideline (ref: No)			1.65***
			(1.61 - 1.69)
Total energy intake (kcal)			1.00***
			(1.00 - 1.00)
Observations	95,475	95,475	95,475

*** p<0.001, ** p<0.01, * p<0.05

For peer review only

Table S8 – Sensitivity analysis: results of ordinal logistic models between any active travel and FV consumption among males in UKB (n=83,213)

VARIABLES	Model 1	Model 2	Model 3
Any active travel (ref: None)	1.38***	1.35***	1.28***
	(1.34 - 1.41)	(1.32 - 1.39)	(1.24 - 1.31)
Age at baseline		1.03***	1.03***
		(1.03 - 1.03)	(1.03 - 1.03)
Ethnic group (ref: White British)			
Other white		1.28***	1.28***
		(1.21 - 1.35)	(1.21 - 1.35)
South Asian		2.02***	2.13***
		(1.81 - 2.25)	(1.91 - 2.38)
Black		1.42***	1.44***
		(1.25 - 1.62)	(1.26 - 1.64)
Chinese		1.59**	1.65***
		(1.20 - 2.12)	(1.24 - 2.20)
Mixed		1.14	1.12
		(0.94 - 1.37)	(0.93 - 1.35)
Other		2.04***	2.10***
		(1.72 - 2.43)	(1.76 - 2.49)
Highest qualification (ref: Degree level)			
A levels/AS levels or equivalent		0.78***	0.79***
		(0.75 - 0.82)	(0.76 - 0.82)
O levels/GCSEs or equivalent		0.76***	0.76***
		(0.73 - 0.79)	(0.73 - 0.79)
CSEs or equivalent		0.74***	0.73***
		(0.69 - 0.79)	(0.68 - 0.78)
NVQ or HND or HNC or equivalent		0.84***	0.83***
		(0.80 - 0.89)	(0.79 - 0.87)
Other professional qualifications		0.87***	0.86***
		(0.82 - 0.93)	(0.80 - 0.92)
No qualifications		0.79***	0.78***
		(0.75 - 0.84)	(0.74 - 0.83)
Occupation class (ref: Higher man / prof)			
Lower managerial / professional		1.03	1.02
		(1.00 - 1.07)	(0.99 - 1.06)
Intermediate occupations		1.06*	1.05
		(1.01 - 1.12)	(1.00 - 1.11)
Small employers & own accounts		1.08	0.99
		(1.00 - 1.16)	(0.92 - 1.07)
Lower supervisory & technical		1.13***	1.02
		(1.06 - 1.21)	(0.95 - 1.09)
Semi-routine occupations		0.99	0.92*
		(0.92 - 1.06)	(0.86 - 0.98)
Routine occupations		1.04	0.94
		(0.97 - 1.13)	(0.87 - 1.01)
Not classified		0.96*	0.92***
		(0.92 - 1.00)	(0.88 - 0.95)
Household income (ref: £<18 000)			
£18,000 to 30,999		1.09***	1.08**
		(1.04 - 1.14)	(1.03 - 1.13)
£31,000 to 51,999		1.20***	1.20***
		(1.15 - 1.26)	(1.14 - 1.25)
£52,000 to 100,000		1.28***	1.29***
		(1.21 - 1.35)	(1.23 - 1.37)
£Greater than 100,000		1.35***	1.35***
		(1.26 - 1.45)	(1.27 - 1.45)

For peer review only

Household size (ref: One)			
2		1.13***	1.12***
		(1.08 - 1.18)	(1.07 - 1.17)
3		1.02	1.02
		(0.97 - 1.07)	(0.97 - 1.07)
4		1.02	1.01
		(0.96 - 1.07)	(0.96 - 1.06)
5+		0.99	0.97
		(0.93 - 1.06)	(0.91 - 1.04)
Region (ref: London)			
North East England		1.05	1.06*
		(1.00 - 1.11)	(1.01 - 1.12)
Yorkshire and the Humber		1.03	1.03
		(0.99 - 1.08)	(0.99 - 1.08)
West Midlands		0.98	0.98
		(0.93 - 1.03)	(0.93 - 1.04)
East Midlands		1.11**	1.11**
		(1.04 - 1.18)	(1.04 - 1.18)
South East England		1.04	1.06*
		(0.98 - 1.10)	(1.01 - 1.12)
South West England		1.07*	1.08**
		(1.02 - 1.13)	(1.02 - 1.13)
North West England		1.00	1.00
		(0.95 - 1.05)	(0.96 - 1.05)
Wales		1.12**	1.14**
		(1.03 - 1.21)	(1.05 - 1.23)
Scotland		0.91**	0.93*
		(0.85 - 0.97)	(0.87 - 0.99)
Townsend deprivation		1.00	1.00
		(0.99 - 1.00)	(0.99 - 1.00)
Urban (ref: Rural)		0.95**	0.96*
		(0.92 - 0.99)	(0.93 - 1.00)
Cars per household (ref: None)			
One		1.01	1.00
		(0.95 - 1.07)	(0.94 - 1.06)
Two		0.92**	0.89***
		(0.86 - 0.98)	(0.83 - 0.95)
Three		0.84***	0.81***
		(0.78 - 0.91)	(0.75 - 0.87)
Four or more		0.81***	0.78***
		(0.73 - 0.90)	(0.71 - 0.87)
Meets physical activity guideline (ref: No)			1.69***
			(1.64 - 1.73)
Total energy intake (kcal)			1.00***
			(1.00 - 1.00)
Observations	83,213	83,213	83,213

*** p<0.001, ** p<0.01, * p<0.05

For peer review only

Table S9 – Sensitivity analysis: results of ordinal logistic models between any active travel and RPM consumption among females in UKB (n=95,475)

VARIABLES	Model 1	Model 2	Model 3
Any active travel (ref: None)	0.86*** (0.83 - 0.88)	0.89*** (0.87 - 0.91)	0.90*** (0.88 - 0.92)
Age at baseline		1.01*** (1.01 - 1.01)	1.01*** (1.01 - 1.01)
Ethnic group (ref: White British)			
Other white		1.02 (0.97 - 1.07)	1.02 (0.97 - 1.07)
South Asian		0.32*** (0.28 - 0.36)	0.33*** (0.29 - 0.37)
Black		1.09 (0.98 - 1.23)	1.09 (0.97 - 1.22)
Chinese		1.83*** (1.46 - 2.30)	1.87*** (1.49 - 2.35)
Mixed		1.00 (0.86 - 1.17)	1.01 (0.86 - 1.18)
Other		0.75*** (0.65 - 0.88)	0.75*** (0.65 - 0.87)
Highest qualification (ref: Degree level)			
A levels/AS levels or equivalent		1.21*** (1.16 - 1.26)	1.22*** (1.18 - 1.27)
O levels/GCSEs or equivalent		1.28*** (1.23 - 1.32)	1.30*** (1.26 - 1.35)
CSEs or equivalent		1.29*** (1.20 - 1.38)	1.33*** (1.24 - 1.42)
NVQ or HND or HNC or equivalent		1.20*** (1.11 - 1.29)	1.24*** (1.15 - 1.33)
Other professional qualifications		1.15*** (1.08 - 1.21)	1.16*** (1.10 - 1.23)
No qualifications		1.29*** (1.22 - 1.37)	1.34*** (1.27 - 1.42)
Occupation class (ref: Higher man / prof)			
Lower managerial / professional		0.96 (0.92 - 1.00)	0.96* (0.92 - 1.00)
Intermediate occupations		1.06* (1.01 - 1.11)	1.06* (1.01 - 1.11)
Small employers & own accounts		1.00 (0.92 - 1.09)	1.01 (0.93 - 1.10)
Lower supervisory & technical		1.04 (0.86 - 1.26)	1.07 (0.89 - 1.29)
Semi-routine occupations		1.07* (1.01 - 1.14)	1.07* (1.01 - 1.14)
Routine occupations		1.19** (1.06 - 1.33)	1.19** (1.07 - 1.34)
Not classified		1.16*** (1.11 - 1.21)	1.16*** (1.11 - 1.22)
Household income (ref: £<18 000)			
£18,000 to 30,999		1.04 (1.00 - 1.08)	1.05* (1.01 - 1.09)
£31,000 to 51,999		0.99 (0.95 - 1.03)	1.00 (0.96 - 1.05)
£52,000 to 100,000		0.99 (0.94 - 1.04)	1.01 (0.96 - 1.06)
£Greater than 100,000		0.99 (0.93 - 1.06)	1.04 (0.97 - 1.11)

For peer review only

Household size (ref: One)			
2		1.41***	1.41***
		(1.36 - 1.47)	(1.35 - 1.46)
3		1.52***	1.50***
		(1.45 - 1.60)	(1.43 - 1.58)
4		1.77***	1.74***
		(1.68 - 1.87)	(1.65 - 1.83)
5+		1.91***	1.86***
		(1.78 - 2.05)	(1.74 - 2.00)
Region (ref: London)			
North East England		0.95*	0.94*
		(0.90 - 1.00)	(0.89 - 0.99)
Yorkshire and the Humber		0.99	0.98
		(0.95 - 1.04)	(0.94 - 1.03)
West Midlands		0.93**	0.92**
		(0.88 - 0.98)	(0.87 - 0.97)
East Midlands		0.97	0.95
		(0.91 - 1.03)	(0.90 - 1.02)
South East England		1.03	1.02
		(0.98 - 1.09)	(0.96 - 1.08)
South West England		0.91***	0.90***
		(0.87 - 0.96)	(0.86 - 0.95)
North West England		1.07**	1.06*
		(1.02 - 1.12)	(1.01 - 1.11)
Wales		0.85***	0.84***
		(0.78 - 0.92)	(0.77 - 0.91)
Scotland		1.08*	1.06
		(1.02 - 1.15)	(1.00 - 1.13)
Townsend deprivation		0.99***	0.99***
		(0.98 - 0.99)	(0.98 - 0.99)
Urban (ref: Rural)		0.97	0.96*
		(0.93 - 1.01)	(0.93 - 1.00)
Cars per household (ref: None)			
One		1.19***	1.19***
		(1.12 - 1.26)	(1.13 - 1.26)
Two		1.34***	1.36***
		(1.26 - 1.43)	(1.27 - 1.45)
Three		1.42***	1.45***
		(1.32 - 1.54)	(1.35 - 1.57)
Four or more		1.36***	1.40***
		(1.23 - 1.51)	(1.26 - 1.55)
Meets physical activity guideline (ref: No)			0.81***
			(0.79 - 0.83)
Total energy intake (kcal)			1.00***
			(1.00 - 1.00)
Observations	95,475	95,475	95,475

*** p<0.001, ** p<0.01, * p<0.05

For peer review only

Table S10 – Sensitivity analysis: results of ordinal logistic models between any active travel and RPM consumption among males in UKB (n=83,213)

VARIABLES	Model 1	Model 2	Model 3
Any active travel (ref: None)	0.86***	0.89***	0.89***
	(0.84 - 0.88)	(0.86 - 0.91)	(0.87 - 0.92)
Age at baseline		1.00*	1.00***
		(1.00 - 1.00)	(1.00 - 1.01)
Ethnic group (ref: White British)			
Other white		0.99	0.99
		(0.93 - 1.05)	(0.94 - 1.05)
South Asian		0.27***	0.28***
		(0.24 - 0.30)	(0.25 - 0.31)
Black		0.95	0.99
		(0.82 - 1.09)	(0.86 - 1.15)
Chinese		1.36	1.42*
		(1.00 - 1.86)	(1.03 - 1.94)
Mixed		1.17	1.19
		(0.96 - 1.44)	(0.97 - 1.46)
Other		0.59***	0.60***
		(0.49 - 0.70)	(0.50 - 0.71)
Highest qualification (ref: Degree level)			
A levels/AS levels or equivalent		1.16***	1.17***
		(1.11 - 1.22)	(1.11 - 1.22)
O levels/GCSEs or equivalent		1.20***	1.22***
		(1.15 - 1.25)	(1.17 - 1.27)
CSEs or equivalent		1.19***	1.22***
		(1.11 - 1.28)	(1.14 - 1.32)
NVQ or HND or HNC or equivalent		1.20***	1.23***
		(1.13 - 1.27)	(1.16 - 1.30)
Other professional qualifications		1.01	1.03
		(0.94 - 1.09)	(0.96 - 1.11)
No qualifications		1.10**	1.13***
		(1.04 - 1.17)	(1.07 - 1.20)
Occupation class (ref: Higher man / prof)			
Lower managerial / professional		0.97	0.96*
		(0.93 - 1.01)	(0.92 - 1.00)
Intermediate occupations		1.02	1.02
		(0.97 - 1.08)	(0.97 - 1.08)
Small employers & own accounts		1.07	1.06
		(0.99 - 1.16)	(0.98 - 1.15)
Lower supervisory & technical		1.13**	1.14***
		(1.05 - 1.22)	(1.05 - 1.22)
Semi-routine occupations		1.19***	1.18***
		(1.10 - 1.28)	(1.10 - 1.27)
Routine occupations		1.22***	1.23***
		(1.12 - 1.33)	(1.13 - 1.33)
Not classified		1.13***	1.13***
		(1.08 - 1.18)	(1.08 - 1.18)
Household income (ref: £<18 000)			
£18,000 to 30,999		0.91***	0.92***
		(0.87 - 0.96)	(0.87 - 0.96)
£31,000 to 51,999		0.96	0.97
		(0.91 - 1.01)	(0.92 - 1.02)
£52,000 to 100,000		0.90***	0.92**
		(0.85 - 0.96)	(0.87 - 0.98)
£Greater than 100,000		0.85***	0.88***
		(0.79 - 0.91)	(0.82 - 0.95)

For peer review only

Household size (ref: One)			
2		1.04	1.05*
		(1.00 - 1.09)	(1.00 - 1.09)
3		1.16***	1.15***
		(1.10 - 1.22)	(1.09 - 1.22)
4		1.27***	1.26***
		(1.21 - 1.35)	(1.19 - 1.33)
5+		1.31***	1.28***
		(1.22 - 1.41)	(1.19 - 1.38)
Region (ref: London)			
North East England		0.94*	0.94*
		(0.89 - 1.00)	(0.89 - 1.00)
Yorkshire and the Humber		0.94**	0.94*
		(0.89 - 0.98)	(0.90 - 0.99)
West Midlands		0.91**	0.91**
		(0.86 - 0.97)	(0.86 - 0.97)
East Midlands		0.86***	0.86***
		(0.80 - 0.92)	(0.80 - 0.92)
South East England		1.00	1.00
		(0.94 - 1.06)	(0.94 - 1.06)
South West England		0.91**	0.91**
		(0.86 - 0.97)	(0.86 - 0.96)
North West England		1.06*	1.07*
		(1.01 - 1.12)	(1.01 - 1.12)
Wales		0.80***	0.80***
		(0.73 - 0.87)	(0.73 - 0.87)
Scotland		1.05	1.04
		(0.98 - 1.12)	(0.97 - 1.12)
Townsend deprivation		1.00	1.00
		(0.99 - 1.00)	(0.99 - 1.00)
Urban (ref: Rural)		0.97	0.97
		(0.94 - 1.01)	(0.93 - 1.01)
Cars per household (ref: None)			
One		1.11**	1.11**
		(1.04 - 1.18)	(1.04 - 1.19)
Two		1.27***	1.28***
		(1.18 - 1.36)	(1.19 - 1.37)
Three		1.44***	1.46***
		(1.33 - 1.57)	(1.34 - 1.59)
Four or more		1.40***	1.43***
		(1.26 - 1.57)	(1.28 - 1.60)
Meets physical activity guideline (ref: No)			0.84***
			(0.82 - 0.87)
Total energy intake (kcal)			1.00***
			(1.00 - 1.00)
Observations	83,213	83,213	83,213

*** p<0.001, ** p<0.01, * p<0.05

For peer review only

STROBE 2007 (v4) checklist of items to be included in reports of observational studies in epidemiology*
Checklist for cohort, case-control, and cross-sectional studies (combined)

Section/Topic	Item #	Recommendation	Reported on page #
**Title and abstract**	1	(*a*) Indicate the study's design with a commonly used term in the title or the abstract	1, 2
		(*b*) Provide in the abstract an informative and balanced summary of what was done and what was found	2
**Introduction**			
Background/rationale	2	Explain the scientific background and rationale for the investigation being reported	4, 5
Objectives	3	State specific objectives, including any pre-specified hypotheses	5
**Methods**			
Study design	4	Present key elements of study design early in the paper	5, 6
Setting	5	Describe the setting, locations, and relevant dates, including periods of recruitment, exposure, follow-up, and data collection	5, 6
Participants	6	(*a*) *Cohort study*—Give the eligibility criteria, and the sources and methods of selection of participants. Describe methods of follow-up<br>*Case-control study*—Give the eligibility criteria, and the sources and methods of case ascertainment and control selection. Give the rationale for the choice of cases and controls<br>*Cross-sectional study*—Give the eligibility criteria, and the sources and methods of selection of participants	5, 6
		(*b*) *Cohort study*—For matched studies, give matching criteria and number of exposed and unexposed<br>*Case-control study*—For matched studies, give matching criteria and the number of controls per case	
Variables	7	Clearly define all outcomes, exposures, predictors, potential confounders, and effect modifiers. Give diagnostic criteria, if applicable	6, 7, 8
Data sources/ measurement	8*	For each variable of interest, give sources of data and details of methods of assessment (measurement). Describe comparability of assessment methods if there is more than one group	6, 7, 8
Bias	9	Describe any efforts to address potential sources of bias	9
Study size	10	Explain how the study size was arrived at	6
Quantitative variables	11	Explain how quantitative variables were handled in the analyses. If applicable, describe which groupings were chosen and why	6, 7, 8
Statistical methods	12	(*a*) Describe all statistical methods, including those used to control for confounding	9
		(*b*) Describe any methods used to examine subgroups and interactions	9, 10
		(*c*) Explain how missing data were addressed	6
		(*d*) *Cohort study*—If applicable, explain how loss to follow-up was addressed<br>*Case-control study*—If applicable, explain how matching of cases and controls was addressed	

For peer review only

		Cross-sectional study—If applicable, describe analytical methods taking account of sampling strategy	
		(*e*) Describe any sensitivity analyses	8
**Results**			
Participants	13*	(a) Report numbers of individuals at each stage of study—eg numbers potentially eligible, examined for eligibility, confirmed eligible, included in the study, completing follow-up, and analysed	6
		(b) Give reasons for non-participation at each stage	NA
		(c) Consider use of a flow diagram	
Descriptive data	14*	(a) Give characteristics of study participants (eg demographic, clinical, social) and information on exposures and potential confounders	10, 17, 18, 19, 20
		(b) Indicate number of participants with missing data for each variable of interest	6, 19, 20
		(c) *Cohort study*—Summarise follow-up time (eg, average and total amount)	
Outcome data	15*	*Cohort study*—Report numbers of outcome events or summary measures over time	
		Case-control study—Report numbers in each exposure category, or summary measures of exposure	
		Cross-sectional study—Report numbers of outcome events or summary measures	10
Main results	16	(*a*) Give unadjusted estimates and, if applicable, confounder-adjusted estimates and their precision (eg, 95% confidence interval). Make clear which confounders were adjusted for and why they were included	11, 12, 21, 22
		(*b*) Report category boundaries when continuous variables were categorized	7, 8, 20
		(*c*) If relevant, consider translating estimates of relative risk into absolute risk for a meaningful time period	
Other analyses	17	Report other analyses done—eg analyses of subgroups and interactions, and sensitivity analyses	9
**Discussion**			
Key results	18	Summarise key results with reference to study objectives	13
Limitations	19	Discuss limitations of the study, taking into account sources of potential bias or imprecision. Discuss both direction and magnitude of any potential bias	13, 14
Interpretation	20	Give a cautious overall interpretation of results considering objectives, limitations, multiplicity of analyses, results from similar studies, and other relevant evidence	14, 15
Generalisability	21	Discuss the generalisability (external validity) of the study results	13
**Other information**			
Funding	22	Give the source of funding and the role of the funders for the present study and, if applicable, for the original study on which the present article is based	15

*Give information separately for cases and controls in case-control studies and, if applicable, for exposed and unexposed groups in cohort and cross-sectional studies.

Note: An Explanation and Elaboration article discusses each checklist item and gives methodological background and published examples of transparent reporting. The STROBE checklist is best used in conjunction with this article (freely available on the Web sites of PLoS Medicine at http://www.plosmedicine.org/, Annals of Internal Medicine at http://www.annals.org/, and Epidemiology at http://www.epidem.com/). Information on the STROBE Initiative is available at www.strobe-statement.org.

For peer review only
